# A subtracted cDNA library identifies genes up-regulated during PHOT1-mediated early step of de-etiolation in tomato (*Solanum lycopersicum* L.)

**DOI:** 10.1186/s12864-016-2613-6

**Published:** 2016-04-18

**Authors:** Petra Hloušková, Véronique Bergougnoux

**Affiliations:** Department of Molecular Biology, Centre of the Region Haná for Biotechnological and Agricultural Research and Faculty of Science, Palacký University in Olomouc, Šlechtitelů 11, CZ-783 71 Olomouc, Czech Republic

**Keywords:** Blue light, De-etiolation, Suppression subtractive hybridization, Tomato (*Solanum lycopersicum* L.)

## Abstract

**Background:**

De-etiolation is the switch from skoto- to photomorphogenesis, enabling the heterotrophic etiolated seedling to develop into an autotrophic plant. Upon exposure to blue light (BL), reduction of hypocotyl growth rate occurs in two phases: a rapid inhibition mediated by phototropin 1 (PHOT1) within the first 30–40 min of illumination, followed by the cryptochrome 1 (CRY1)-controlled establishment of the steady-state growth rate. Although some information is available for CRY1-mediated de-etiolation, less attention has been given to the PHOT1 phase of de-etiolation.

**Results:**

We generated a subtracted cDNA library using the suppression subtractive hybridization method to investigate the molecular mechanisms of BL-induced de-etiolation in tomato (*Solanum lycopersicum* L.), an economically important crop. We focused our interest on the first 30 min following the exposure to BL when PHOT1 is required to induce the process. Our library generated 152 expressed sequence tags that were found to be rapidly accumulated upon exposure to BL and consequently potentially regulated by PHOT1. Annotation revealed that biological functions such as modification of chromatin structure, cell wall modification, and transcription/translation comprise an important part of events contributing to the establishment of photomorphogenesis in young tomato seedlings. Our conclusions based on bioinformatics data were supported by qRT-PCR analyses the specific investigation of V-H^+^-ATPase during de-etiolation in tomato.

**Conclusions:**

Our study provides the first report dealing with understanding the PHOT1-mediated phase of de-etiolation. Using subtractive cDNA library, we were able to identify important regulatory mechanisms. The profound induction of transcription/translation, as well as modification of chromatin structure, is relevant in regard to the fact that the entry into photomorphogenesis is based on a deep reprograming of the cell. Also, we postulated that BL restrains the cell expansion by the rapid modification of the cell wall.

**Electronic supplementary material:**

The online version of this article (doi:10.1186/s12864-016-2613-6) contains supplementary material, which is available to authorized users.

## Background

Light is one of the most important environmental factors influencing plants throughout their life spans. Blue and red/far-red portions of light can be considered as the most active rays within the light spectrum for regulating plant growth and development. As sessile organisms, plants have evolved highly sophisticated unique photoreceptors to sense light. They possess three main classes of photoreceptors: phytochromes (PHY), cryptochromes (CRY), and phototropins (PHOT), capable of absorbing red/far-red, blue, and blue light, respectively [1]. Not only is light the primary source of energy for photosynthesis, but it also regulates numerous physiological responses, such as shade avoidance, flowering, germination, tropisms, and de-etiolation [2]. De-etiolation occurs during early seedling development. In dicotyledonous plants, the hypocotyl (embryonic stem) connects the two cotyledons (embryonic leaves) to the root. When germinated in darkness in the soil, the hypocotyl expands toward the surface in order to place the shoot apical meristem in an environment suitable to ensure photoautotrophic growth. When the seedling emerges from the soil, it perceives light; the hypocotyl stops growing, the cotyledons unfold and green, the chloroplasts differentiate, and finally photosynthetic growth is initiated [3]. As almost all of the hypocotyl’s cells are formed during embryogenesis; only a few cell divisions occur in the hypocotyl during etiolation, being limited to the development of stomata [3]. For example, in *Arabidopsis*, the hypocotyl consisting of only 20 epidermal cells elongates more than 100-fold its embryonic length [3].

Hypocotyl de-etiolation is regulated by the three mentioned photoreceptor families. Nevertheless, at equal irradiances, blue light (BL) is more effective than red light as it inhibits growth more quickly and to a greater extent [4]. Using *cry1 Arabidopsis* mutant defective in BL-induced de-etiolation, studies have demonstrated that CRY1 is the BL receptor involved in the control of hypocotyl elongation [5, 6]. Using computer-assisted electronic image capture, however, Parks and co-authors [7] demonstrated that in *cry1* seedlings hypocotyl growth inhibition begins to develop within approximately 30 sec of BL irradiation and reaches the same maximum level displayed by wild-type seedlings after approximately 30 min of BL treatment. At this point, *cry1* seedling growth accelerates, soon attaining the growth rate observed for darkness-grown seedlings. This experiment demonstrated that BL-mediated hypocotyl inhibition in *Arabidopsis* occurs in two genetically independent phases [7]. A few years later, while applying the same method to different *Arabidopsis* photoreceptor mutants, Folta and Spalding [8] identified PHOT1 as being involved in the rapid phase of BL-mediated hypocotyl growth inhibition.

The PHOT1 signaling pathway has been studied extensively in the phase of stomata opening. In response to BL, plasma membrane H^+^-ATPases in the guard cells are activated. This induces a negative electrical potential across the plasma membrane and drives K^+^ uptake. Ions and metabolites enter the cell concomitantly with water uptake, thereby increasing turgor pressure and resulting in the opening of the stomata. The plasma membrane H^+^-ATPase is activated by phosphorylation of its C-terminus with a concomitant binding of the 14-3-3 proteins [9]. By comparison, the mechanisms involved in PHOT1-mediated de-etiolation are still poorly understood. Nevertheless, genetic, biochemical, and physiological studies have begun to delineate the signaling pathway initiated after the onset of BL excitation. Evidence has accumulated to prove that excitation of PHOT1 induces a rapid activation of Ca^2+^ channels at the plasma membrane, leading to an increased concentration of cytosolic Ca^2+^ [10], [11]. To our knowledge, few events acting downstream of PHOT1 have been identified during de-etiolation [8], [12]. Therefore, it remains challenging to identify the PHOT1-signaling pathway during de-etiolation. All analyses to date have been performed on plant models, most notably in *Arabidopsis*. Little or no information is available from important crop species. For several years, we have focused on understanding the role of BL in the growth and development of tomato (*Solanum lycopersicum* L.), an economically important crop [13]. We previously demonstrated that in etiolated tomato seedlings exposed to BL the reduction of the hypocotyl growth rate is a two-step process [14]. Based on the knowledge coming from studies on Arabidopsis, we hypothesized that the first rapid inhibition might be triggered by PHOT1, and that the steady-state rate of growth might be established by CRY1.

Suppression subtractive hybridization (SSH) is a powerful approach which allows the comparison of two samples (tester and driver) and the identification of differentially regulated genes [15]. Indeed, SSH is a combination of normalization which equalizes the abundance of cDNA within the target population and subtraction which excludes sequences common to both the tester and the driver [15]. Therefore, SSH identifies not only abundant differentially expressed genes but also rare transcripts which were enriched during the process. This latest category is of high interest as it can represent a pool of unknown genes. This method does not require an in-depth knowledge of the genome under study and can thus be applied easily to non-model species [16], [17], [18], [19]. In the present study, we used the SSH approach to identify the molecular mechanisms of the PHOT1-mediated rapid inhibition of hypocotyl elongation in tomato. Our current results provide evidence that a complex network is quickly activated by exposure to BL in order to induce de-etiolation.

## Methods

### Plant materials and light treatment

The tomato cultivar Rutgers was used in this study. Sterile cultures were obtained as described in [20]. After germination in darkness at 23 °C, germinated seeds were transferred in the dark for 3 additional days to a culture chamber maintained at 23 °C. For BL-induced de-etiolation, dishes containing 3-day-old etiolated seedlings were transferred for 30 min under BL provided by fluorescent lamps (BL; TL-D 36 W/Blue, Phillips; total photon fluence rate 10 μmol.m^−2^.s^−1^).

### RNA extraction and subtractive library construction

**Fig. 1:**
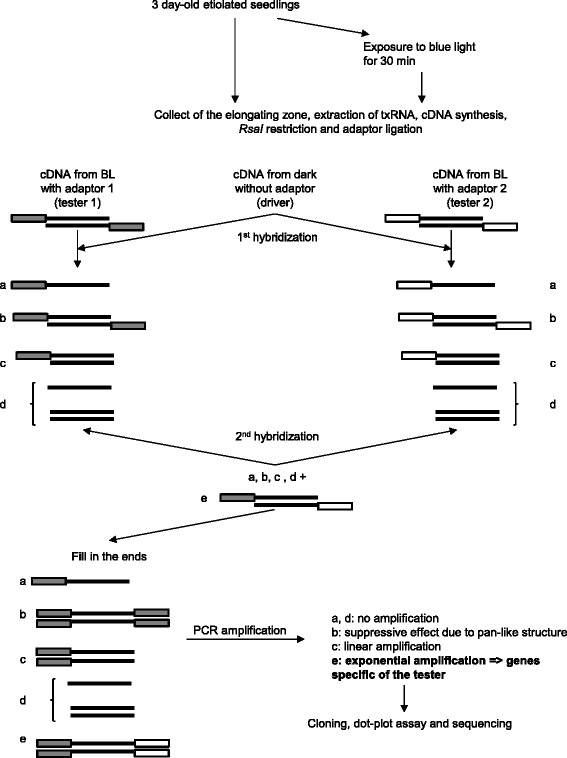
Schematic representation of the suppression subtractive hybridization adapted to the present study from [15]

For all experiments, the elongating zone of the hypocotyl of 3-day-old etiolated seedlings was excised from the rest of the seedling. either under green safety light (dark control) or under BL after 30 min of exposure to BL. The elongating zone corresponding to the portion of hypocotyl situated beneath the hook and cotyledons was limited to the upper third of the hypocotyl as described in [14]. Samples were immediately frozen in liquid nitrogen and stored at −80 °C before RNA isolation. Frozen tissues were ground in liquid nitrogen using a mortar and pestle. Total RNA was extracted using an RNeasy Plant Mini Kit (Qiagen). Remaining traces of DNA were removed with a recombinant DNAseI (Takara) and RNA were subsequently purified by a phenol:chloroform:isoamyl alcohol (25:24:1) step. PolyA^+^ mRNA were purified using the Straight A’s mRNA isolation system (Novagen). Quantity and quality of mRNA were checked by spectrophotometer and electrophoresis. The suppression subtractive hybridization (SSH) library was constructed according to the instructions of the PCR-Select cDNA Subtraction Kit (Clontech). The principle of SSH library is illustrated by the Fig. 1. In order to identify genes up-regulated by the exposure to BL, subtractive hybridization was performed using cDNA from hypocotyls exposed for 30 min to BL as tester against cDNA from control hypocotyls (not exposed to BL) as driver. The subtraction efficiency was evaluated by a PCR reaction amplifying a region of the tomato *EF1α* gene and the PCR product was analyzed after 15, 20, 25, 30, and 35 cycles.

### Cloning, screening for differential expression, sequencing and analysis

Secondary SSH-PCR products were inserted into pGEM-T Easy Vector (Promega) and cloned into *Escherichia coli* DH5α strain. A blue–white screening was performed in order to obtain a bank of subtracted ESTs. White colonies were picked and grown in 96-well microtiter plates in a lysogeny broth medium containing ampicillin (100 mg.L^−1^). Screen for differentially expressed ESTs was performed by dot blot hybridization as described in the PCR-select cDNA subtraction kit (Clontech). For this purpose, plasmids were isolated, quantified and transferred to Hybond-N+ nylon membranes. Membranes were prepared in duplicates with equal amounts of plasmids and were hybridized either with the BL-specific tester probe or the dark-specific driver probe, both DIG-labelled. Detection was performed with an anti-digoxigenin antibody coupled with a horse radish peroxidase. Cold detection was performed by enhanced chemiluminescent detection and exposure to X-ray films. Autoradiographies were scanned and the intensity of the dots was determined using ImageJ software. The dot intensity of a specific clone obtained with the BL-specific probe was compared to that obtained with the dark-specific probe. All clones showing higher intensity with BL-specific probe compared to dark-specific probe were selected and sequenced by Macrogen (Korea). A total of 168 ESTs were found to be differentially expressed. Their sizes ranged from 128 to 1387 bp, with an average size of 447 bp. Because during the process of library preparation the cDNA were restricted by *RsaI*, the first step of the analysis was to retrieve the full-length of the gene and the corresponding protein for downstream analyses by BLAST against the tomato database at SolGenomics Network. The gene ontology annotation was performed with Blast2GO according to plant-specific Gene Ontology terms [21]. Concurrently, the functional annotation was performed by Mercator/MapMan which allows attributing DEGs a functional pathway [22], [23].

### Quantitative real time-PCR

To confirm the differential expression of the selected ESTs, total RNA was extracted from the elongating zone of 3 day-old etiolated seedlings exposed or not to BL as previously described. Reverse transcription was performed from 1 μg of the total RNA according to the instructions of the PrimeScript kit (Takara), followed by subsequent RNaseH treatment (Takara) and purification on a Macherey-Nagel column to remove any compounds which could have an inhibitory activity during subsequent steps. For quantitative real-time PCR, cDNA samples were diluted by 5-fold and used in a reaction containing SYBR Premix ExTaq (Takara) PCR Master Mix and 200 nM of each primer. Three technical repeats were run for each sample on the Mx3000P thermocycler (Stratagene) in a two-step amplification program. The initial denaturation at 94 °C for 10 s was followed by 40 cycles of 94 °C for 5 s and 60 °C for 20 s. A dissociation curve was obtained for each sample. Three independent biological repeats were analyzed for each sample. Each independent biological replicate represents a pool of 20 to 25 explants. Cycle threshold values were normalized in respect to the *PP2Acs* gene [24]. The analysis of different usual housekeeping genes was performed and confirmed that *PP2Acs* gene is the most stable in the conditions of the present study (data not shown). Differences in cycle numbers during the linear amplification phase between the samples. After determination of primer efficiency, the Pfaffl method was used to determine the fold change in gene expression [25]. The relative quantification was made compared to the dark control sample. The results are expressed in term of fold change and represent the average ± standard error on mean (SEM) of 3 independent biological replicates. Primers were designed using default parameters of IDT qPCR assay design and checked with OligoAnalyzer tool (http://eu.idtdna.com/scitools/Applications/RealTimePCR/). Their specificity was checked by blast restricting to tomato database. Sequences of primers and their efficiency are given in Table 1.

**Table 1:** Primers used in quantitative real-time PCR

Identification	Description of the gene	Primers	Primer efficiency
A-E4	Mitogen-activated protein kinase	F: 5′- GAAGATGAGAAACCACAAGCG	90 %
		R: 5′- CATTCTGAGGAACTTGGAGAGG	
C-G1	Importin subunit alpha1a	F: 5′- GAACTCATTTTGTGCCCCATC	92 %
		R: 5′- GCTGAGGGATTGGAAAAGATTG	
E188	Intracellular Ras-group-related LRR protein 9	F: 5′- GAGAGGCAGGATTGGAGATTG	94 %
		R: 5′- TCCGCATCCTTCAACATCTTC	
E-E3	Polyadenylate-binding protein RBP47	F: 5′- TCCTAATGAGCCTAACAAACCTG	92 %
		R: 5′- TCCGTCTTATTGCCTTCCAC	
VHA-A1	V-ATPase catalytic subunit A1	F: 5’- CGAGAAGGAAAGCGAGTATGG	107 %
		R: 5’- TCATTCACCATCAGACCAGC	
B-D5	Vacuolar H + -ATPase V0 sector	F: 5′- GCAGTCATTATCAGTACCGGG	89 %
		R: 5′-TCTAACACCAGCATCACCAAC	
B-E2	Pectin acetylesterase	F: 5’-CACACCCACAAAGAGAAACAG	103 %
		R: 5’-TTCCAAGAATGCCCCTTCAG	
12.	XTH	F: 5’-AGAGGTGGGCTTGAGAAAAC	93 %
		R: 5’-GAACCCAACGAAGTCTCCTATAC	
B-D9	26S proteasome	F: 5’-TCTTGTCCTCTTTCTGTTCCTTATC	95 %
		R: 5’-AATCCTTGCCTCACTTCCAG	
C-A3	Histone H2B	F: 5′- TTGGTAACAGCCTTAGTTCCTC	89 %
		R: 5′- AAAGCCTACCATCACTTCTCG	
PP2ACS	PROTEIN PHOSPHATASE 2A catalytic subunit	F: 5’- CGATGTGTGATCTCCTATGGTC	98 %
		R: 5’- AAGCTGATGGGCTCTAGAAATC	

The non-parametric Mann-Whitney *U* test (Statistica 12) was used to determine the significance of the results.

### Bafilomycin A1 treatment

Sterile cultures were obtained as described in [19]. After germination in darkness, germinated seeds were transferred on a Murashige and Skoog medium containing varying concentrations of bafilomycin A1. For condition of darkness, dishes were wrapped in aluminum foil and placed in the culture chamber; for light conditions, dishes were cultivated in a culture chamber illuminated with BL (total photon fluence rate 10 μmol.m^−2^.s^−1^). After 5 days, the length of the hypocotyl was measured to the nearest millimeter with a ruler. The graph represents the mean ± SEM; an average of 45 seedlings were measured for each condition. The non-parametric Kruskal-Wallis ANOVA (Statistica 12) was performed in order to support the statistical significance of the data.

## Results and discussion

### Construction of the subtracted cDNA library and analysis

**Fig. 2:**
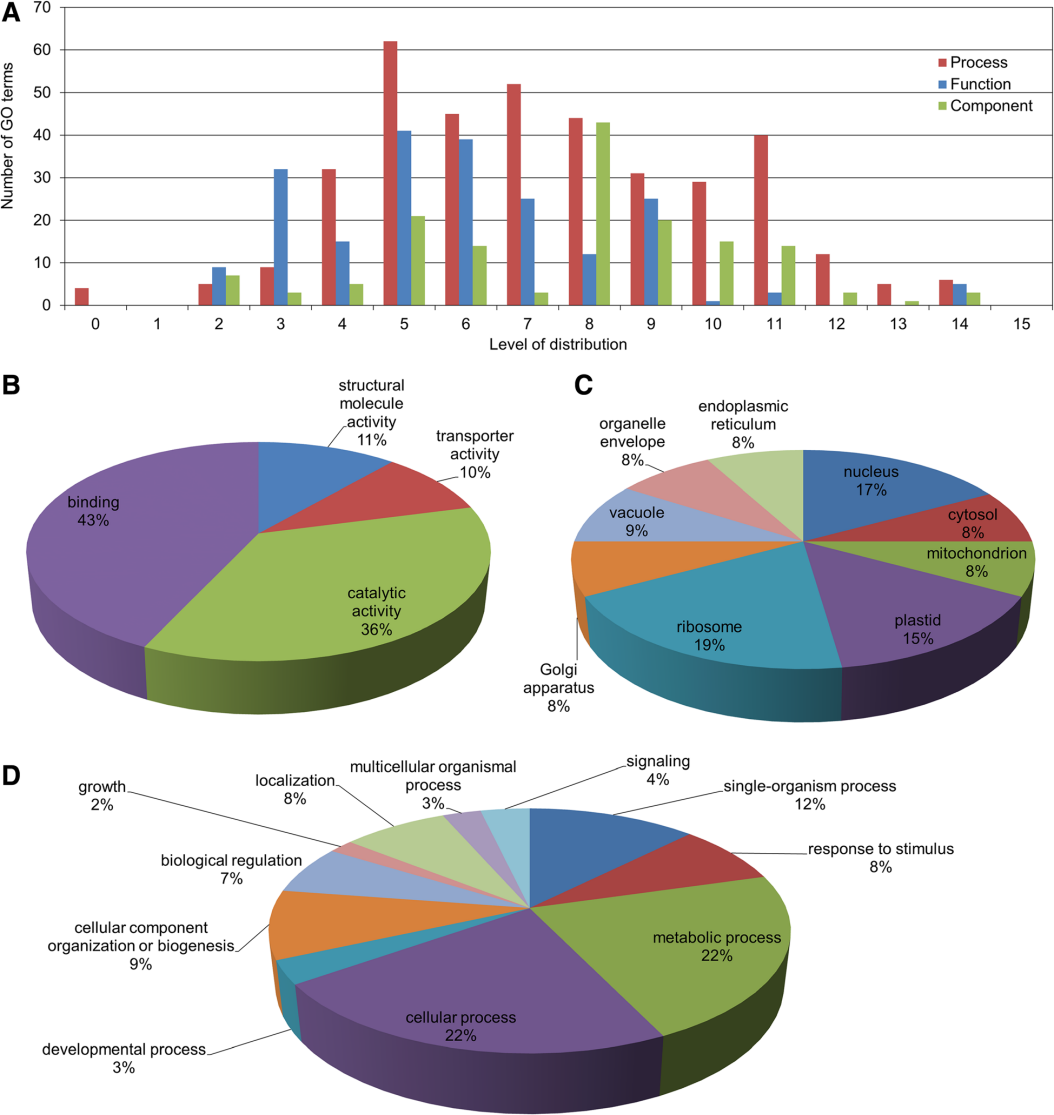
Gene Ontology terms distribution. Number of Gene Ontology terms per level of distribution (**a**), Gene Ontology terms distribution by molecular functions (**b**), cellular components (**c**), and biological processes (**d**) vocabularies. In **b** binding and catalytic activities were the most represented molecular functions. In **c** the most represented categories were ribosome, followed by nucleus and plastid. In **d** the most abundant categories were metabolic and cellular processes

**Fig. 3:**
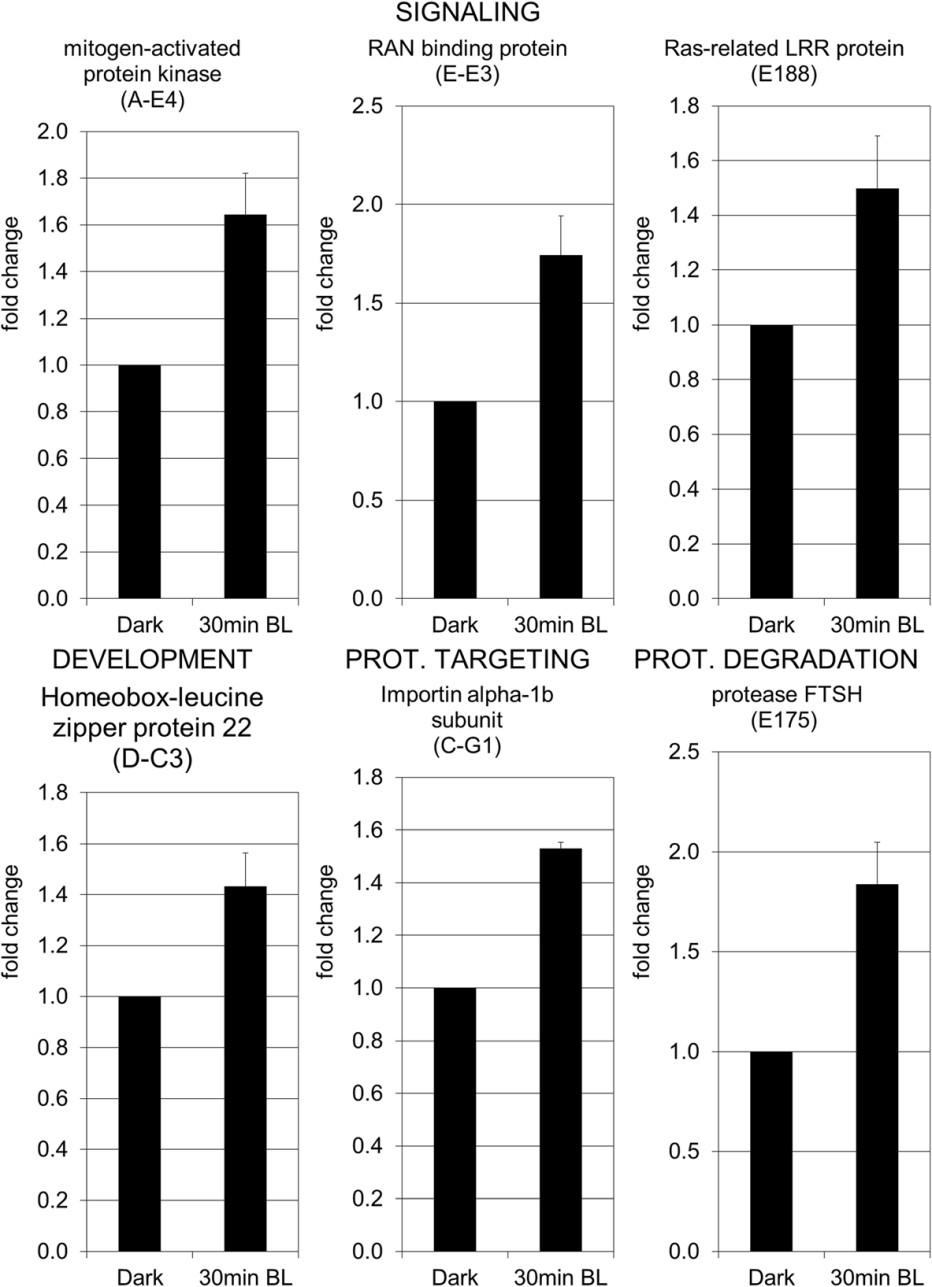
Analysis by quantitative real-time PCR of expression of selected genes belonging to different functional categories. The data represent the average fold change of 3 independent biological replicates ± SEM. Normalization was done using the *pp2ase* gene as housekeeping gene. Fold change was calculated compared to the value obtained for the dark control sample. The non-parametric Mann-Whitney *U* test (Statistica 12) was used to determine the significance of the results

In order to study the molecular events of the rapid inhibition of tomato hypocotyl growth observed within the first 30 min following exposure to BL, a SSH library was constructed and screened for genes whose expression is stimulated by BL. Two contrasting mRNA samples were extracted. One sample, extracted from the elongating zone of the hypocotyl of seedlings grown in darkness and exposed for 30 min to BL (10 μmol.m^−2^.s^−1^), potentially containing differentially expressed genes, was used as the tester. The second sample, isolated from the elongating zone of the hypocotyl of seedlings grown only in darkness, constituted the driver that should express transcripts common to both samples and eliminated during the process of subtraction. Due to technical limitation, 500 putative subtracted clones were randomly picked and used in cDNA dot-plot array for differential screening. Clones were considered for sequencing when they hybridized only to the BL-specific probes or showed higher intensity with the BL-specific probe than with dark-specific probe. In these conditions, we determined that 168 ESTs were potentially differentially expressed. After BLAST analysis, 17 sequences from 168 were found to be redundant, bringing to 151 the number of expressed sequence tag (ESTs) encoding proteins. The ontology annotation was performed using Blast2GO according to plant-specific Gene Ontology terms [21]. Computational analysis using the software Blast2GO enabled annotation of the expressed sequences according to the terms of the three main Gene Ontology vocabularies (i.e., cellular compartment, molecular function and biological process; Fig. 2). Concerning molecular function, the most represented categories were those of binding and catalytic activities (Fig. 2b). Regarding cellular compartments, the most represented were ribosome, plastid, and nucleus, together accounting for more than 50 % of total annotations (Fig. 2c). When taking into consideration the most relevant level of distribution for the biological process (i.e., level 8, as shown in Fig. 2a), more than 40 categories were found for the biological process vocabulary (data not shown). The number of categories was therefore simplified to level 2 of the distribution (Fig. 2d). The functional annotation was performed with Mercator, using the last updated version of the tomato annotation (ITAG2.4) [22]. Identical description of the ESTs was obtained when the annotation was performed by Blast2Go, KOG attribution or Blast against the specific annotated tomato genome ITAG2.4 (Additional file: 1 Table S1). The Table 2 shows the number of sequences which enter the different categories. Twenty seven sequences could not be annotated. The functional annotations “Protein: synthesis, targeting, postranslation modification, degradation” and “RNA: processing, transcription, regulation of transcription” were the most represented, including 33 and 12 sequences, respectively. More detailed information can be found in Additional file 1: Table S1. For all genes tested, qPCR confirmed the differential expression detected by the screening of the cDNA library, meaning the up-regulation of the expression of ESTs as soon as 30 min after exposure to BL (Fig. 3). Below, we discuss the potential involvement of various genes in the rapid inhibition of hypocotyl growth induced by 30 min of exposure to BL and mediated by PHOT1.

**Table 2:** Functional categories of up-regulated genes

BIN categories	Number of sequences
Photosynthesis	1
Minor carbohydrates	3
Glycolysis	1
Fermentation	1
Mitochondrial electron transport/ATP synthesis	4
Cell wall	7
Lipid metabolism	3
Amino acid metabolism	7
Stress	11
Redox	2
Miscellaneous enzyme families	4
RNA: processing, transcription, regulation of transcription	12
DNA: synthesis/chromatin structure, repair	4
Protein: synthesis, targeting, postranslation modification, degradation	33
Signalling	8
Cell organisation	3
Cell cycle	1
Cell, vesicle transport	2
Development	5
Transport	12
No ontology	8
Unknown	19

### Translation and transcription

Sixteen differentially expressed sequences were predicted to encode proteins involved in translation, RNA processing and modification (ribosomal proteins), transcription (eukaryotic initiation factors), and chromatin structure and dynamics (Histone 2B – H2B, Histone H2A).

**Fig. 4:**
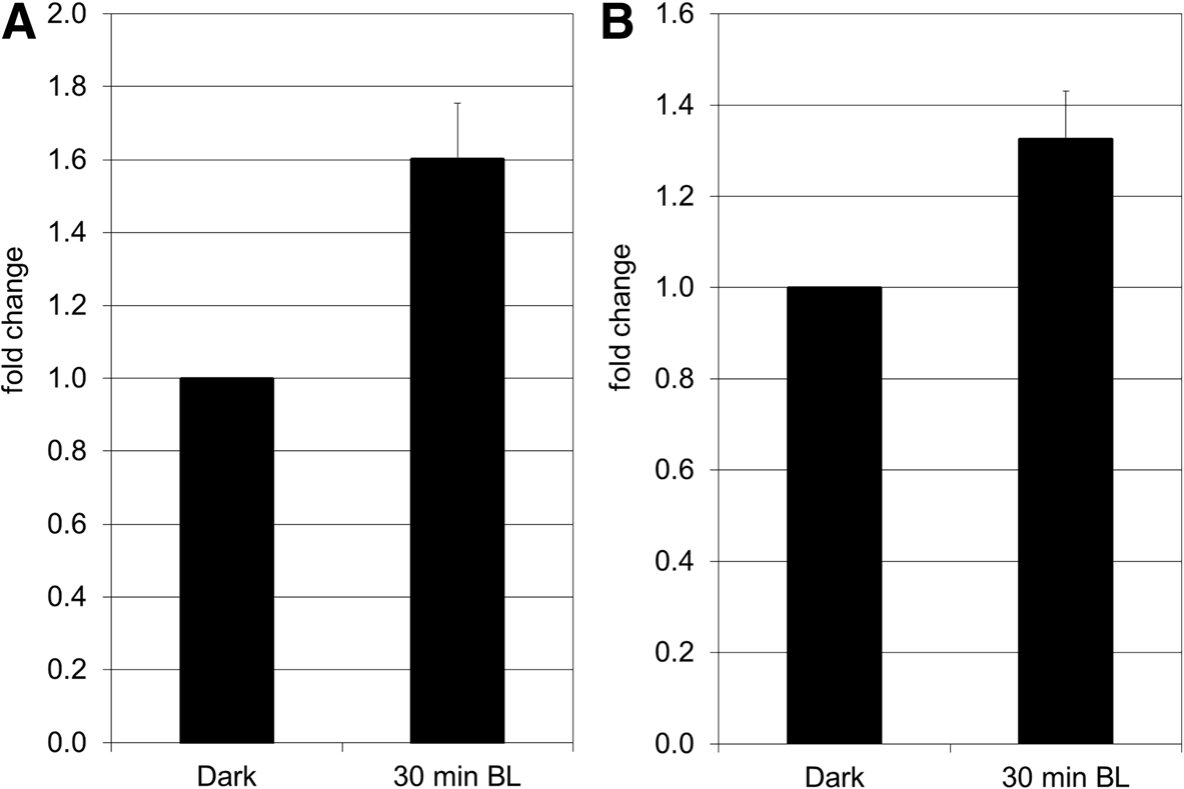
Analysis by qPCR of the expression of two genes encoding proteins involved in translation and transcription: Histone 2B (**a**) and 26S proteasome regulatory complex, subunit RPN10/PSMD4 (**b**). The data represent the average fold change of 3 independent biological replicates ± SEM. Normalization was done using the *pp2ase* gene as housekeeping gene. Fold change was calculated compared to the value obtained for the dark control sample. The non-parametric Mann-Whitney *U* test (Statistica 12) was used to determine the significance of the results

In our study, we found by SSH screening and confirmed by qPCR analysis that H2B is up-regulated during PHOT1-mediated de-etiolation in tomato, indicating that it could play a role during the establishment of photomorphogenesis in tomato (Fig. 4a). Histones form the protein core of the nucleosome around which the DNA helix is wrapped. In this compact state, histones block the association of transcription factors to their binding sites, thus repressing transcription. Several post-translational modifications (acetylation, methylation or phosphorylation) of histone “tails” can influence nucleosome compaction and access to DNA. Moreover, their spatio-temporal regulation as well as their ability for cross-talk renders the regulation of gene expression even more complex [26]. In plants, chromatin remodeling plays an important role during plant growth and development, especially in response to light. Indeed, a large-scale reorganization of chromatin can be observed during the floral transition in *Arabidopsis* [27]. During de-etiolation, the perception of light induces a remarkable reprogramming of gene expression that leads the heterotrophic seedling to become an autotrophic organism which will be able to complete its life cycle. In darkness, the photomorphogenic repressor DET1 binds to the H2B tails of the nucleosomes surrounding the genes which are repressed in this condition. When light is perceived, the H2B acetylation concomitant with the release of DET1 enables the activation of genes involved in photomorphogenesis [28]. It would be interesting in the near future to validate the potential involvement of H2B in the control of de-etiolation in tomato and thereby to follow the relationship between gene expression and H2B enrichment during this process. Finally, we could identify and confirm that the subunit RPN10/PSMD4 of the 26S proteasome regulatory complex is up-regulated during de-etiolation (Fig. 4b). For this reason, it is tempting to hypothesize that light-regulated histone expression/modification and ubiquitin-proteasome-mediated protein degradation might interact during tomato de-etiolation.

### Cell wall modification

**Fig. 5:**
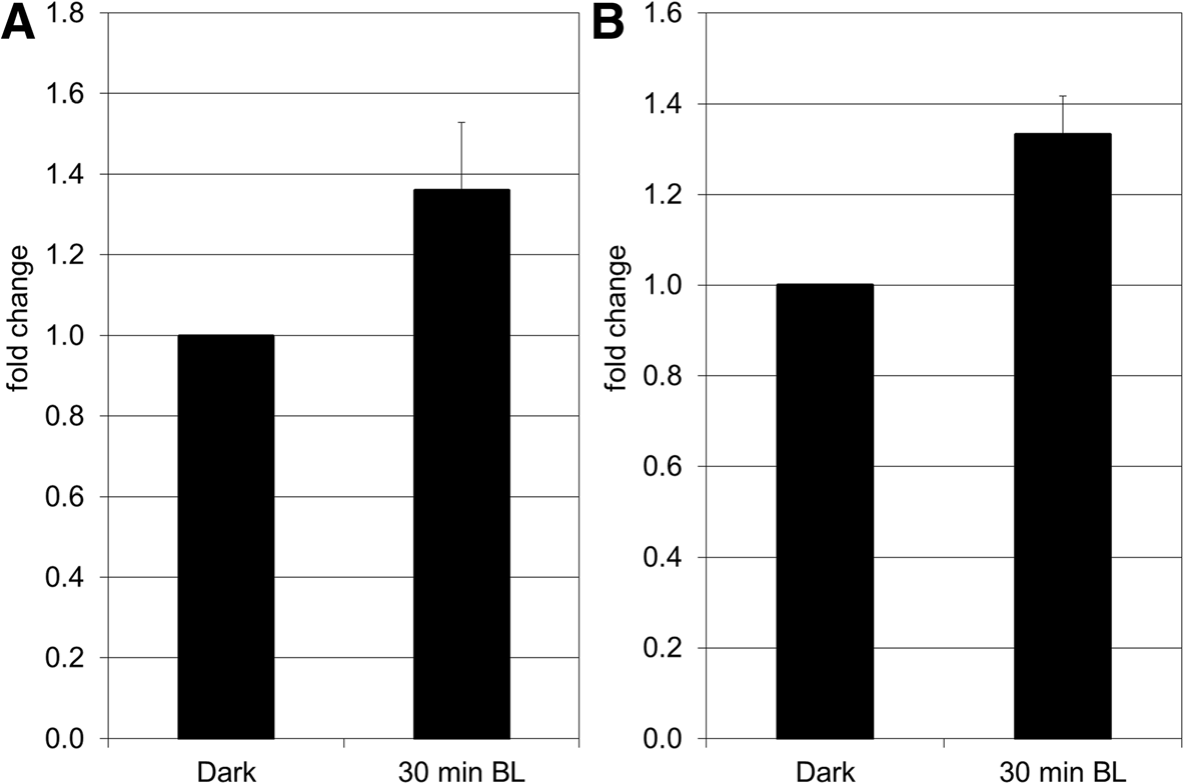
Analysis by qPCR of the expression of two genes encoding proteins involved in cell wall modification: xyloglucan endotransglucosylase-hydrolase/XTH (**a**) and pectin acetylesterase (**b**). The data represent the average fold change of 3 independent biological replicates ± SEM. Normalization was done using the *pp2ase* gene as housekeeping gene. Fold change was calculated compared to the value obtained for the dark control sample. The non-parametric Mann-Whitney *U* test (Statistica 12) was used to determine the significance of the results

In our study, seven tomato EST encoding proteins involved in cell wall modification were found to be up-regulated in the etiolated hypocotyl of tomato seedlings exposed for 30 min to BL: pectin acetylesterase, pectinesterase, xyloglucan endotransglucosylase-hydrolase 1 (XTH), or endoglucanase. This suggested that de-etiolation induced a strong modification of the cell wall structure and/or composition. Plant cell walls consist of a complex network of cellulose microfibrils embedded in a matrix of hemicelluloses (mainly xyloglucans), pectins, and glycoproteins [29]. During cell maturation, cell walls lose the ability to expand [30]. Growth cessation is accompanied by cell wall tightening [31]. Various modifications of cell wall structure during maturation have been proposed, including changes in hemicellulose. For example, the maturation of pea tissues is characterized by an increase in the total amount of xyloglucan [32]. Xyloglucan endotransglucosylase/hydrolases (XTH) are enzymes capable of modifying xyloglucan during cell expansion. They comprise a subgroup of the glycoside hydrolase family 16. XTH proteins characterized to date have endotransglycosylase (XET) or hydrolase (XEH) activities towards xyloglucans, or both. Their phylogenic study indicates that they are organized into three groups: I/II, III-a, and III-b. Only members of the III-a group are strict XEH [33]. Transgenic tomatoes with altered levels of XTH gene showed higher XET activity, lower hemicellulose depolymerization and reduced fruit softening during ripening. This suggests that XET could have a role in maintaining the structural integrity of the cell wall [34], [35]. Thus, whereas some XTH members are critical in promoting cell wall expansion, others are required for wall strengthening in cells that have completed the expansion process [36]. The analysis by qPCR of the XTH identified by SSH screening confirmed that it is up-regulated by BL (Clone 12; Fig. 5a). Based on the aforementioned literature, we can assume its role in cell wall strengthening during de-etiolation. Pectins, comprising another important cell wall component, are synthesized in the *cis*-Golgi, methyl-esterified in the medial-Golgi, substituted in the *trans*-Golgi, and then secreted into the cell wall. Zhao and co-authors [37] reported that de-esterification of methyl-esterified pectin may also be associated with growth cessation in both grasses and dicotyledons and may contribute to wall tightening by strengthening pectin–calcium networks. Pectin acetylation is another modification of pectins which probably occurs between the Golgi and the cell wall during pectin exocytosis. Its occurrence and function are poorly understood. The degree of O-acetylation of pectin changes during growth and differentiation of plant tissues, but also in response to environmental conditions. Pectin acetylesterases trigger the deacetylation of pectin. The overexpression of the black cottonwood (*Populus trichocarpa*) *PAE1* gene in tobacco has been shown to impair the cellular elongation of floral organs. Thus, it appears that pectin acetylesterases function as an important regulator of pectin acetylation status to affect the physiochemical properties of the cell wall’s polysaccharides and consequently to affect cell extensibility [38]. The confirmation by qPCR that pectin acetylesterase (B-E2; Fig. 5b) is up-regulated by BL supports the hypothesis that they are actors of the inhibition of cell expansion which occurs during de-etiolation.

Based on our data and the analysis of the literature, we can hypothesize that exposure to BL rapidly induces changes in cell wall properties, namely extensibility. It would be thus interesting to validate this hypothesis through physico-chemical measurement of the cell wall of tomato seedlings’ hypocotyl during BL-induced de-etiolation.

### Role of vacuolar H^+^-ATPase during de-etiolation

**Fig. 6:**
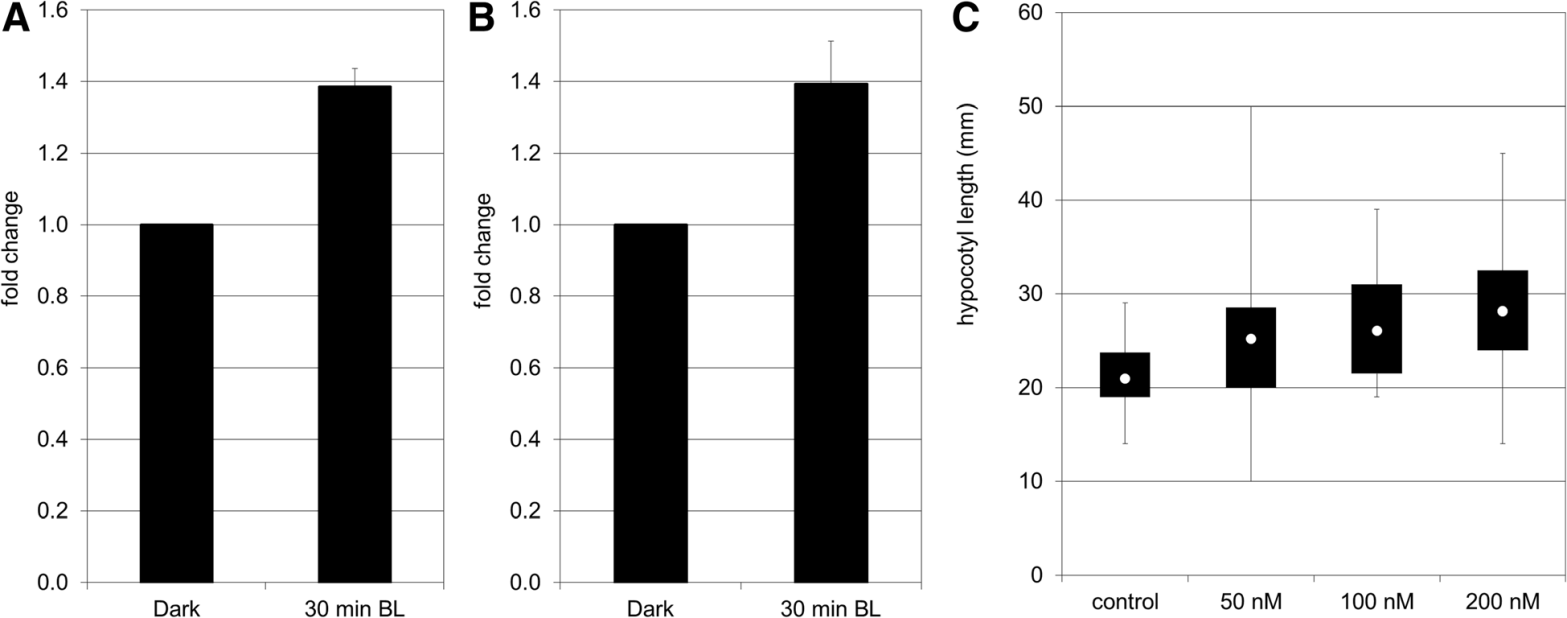
Involvement of V-H^+^-ATPase during de-etiolation of tomato seedlings. **a** Analysis by qPCR of V- ATPase subunit c2 (B-D5) during de-etiolation. The data represent the average fold change of 3 independent biological replicates ± SEM. Normalization was done using the *pp2ase* gene as housekeeping gene. Fold change was calculated compared to the value obtained for the dark control sample. The non-parametric Mann-Whitney *U* test (Statistica 12) was used to determine the significance of the results. **b** Analysis by qPCR of VHA-A1 subunit during de-etiolation. The data represent the average fold change of 3 independent biological replicates ± SEM. Normalization was done using the *pp2ase* gene as housekeeping gene. Fold change was calculated compared to the value obtained for the dark control sample. The non-parametric Mann-Whitney *U* test (Statistica 12) was used to determine the significance of the results. **c** Effect of bafilomycin A1 on hypocotyl growth of tomato seedlings grown in BL . Germinated seeds were grown either in darkness or under constant BL on Murashige and Skoog medium containing varying concentrations of BafA1. After 5 days of growth, the length of hypocotyl was measured with a ruler to the nearest millimeter. The data are presented as boxes and whiskers. The whiskers represent the range of the data; the white dot within the box indicates the median value, while the boxes’ lower and upper boundaries indicate the first and third quartiles, respectively. An average of 45 plantlets coming from independent replicates was measured. The non-parametric Kruskal-Wallis Anova with multiple comparison of mean rank was used for statistical significance of the data (Software: Statistica 12); a: statistically different from the control condition with *p*-value ≤ 0.01

In tomato, three ESTs encoding vacuolar H + -ATPase (V-ATPase: 9, B-D5, E169) subunits were found to be up-regulated during PHOT1-mediated inhibition of hypocotyl growth. This was confirmed by qPCR for the V-type H^+^-ATPase subunit c2 (B-D5; Fig. 6a). Considering the role of V-ATPase during de-etiolationis important if one considers that hypocotyl growth in darkness does not require the division of cortical or epidermal cells and cells elongate along an acropetal spatial and temporal gradient [39]. Cell expansion is achieved by: i) increase in cell ploidy via endoreduplication, and ii) osmotic water uptake into the vacuole, creating the turgor pressure necessary for the irreversible extension of the cell wall caused by the synthesis, incorporation, and cross-linking of new cell wall components. The cell expansion is restricted by cell wall extensibility [40], [41]. V-ATPases are potentially involved in creating or regulating turgor pressure. They represent a major fraction of the total tonoplast proteins. V-ATPases also are present in the *trans*-Golgi network (TGN), where they are essential for its proper function [42]. Whereas inhibition of the tonoplast-localized V-ATPase does not affect cell expansion, inhibition of that which is TGN-localized is sufficient to restrict cell expansion [43]. Moreover, the *det3* mutant, a possible negative regulator of photomorphogenesis affected in V-ATPase function, was originally proposed to be impaired in vacuolar solute uptake resulting in adequate turgor pressure for cell expansion [3]. Recent evidence has shown that a cell wall defect in the mutant is responsible for its reduced hypocotyl cell expansion [43]. Together, these data indicate that V-ATPase plays a role in cell wall integrity/synthesis through its function in the TGN-mediated secretory pathway, thereby participating in the restriction of cell expansion.

In eukaryotes, V-ATPase consists of at least 12 distinct subunits organized in two large subcomplexes: the cytosolic V1 and membrane Vo subcomplexes. The cytosolic V1 complex is constituted of subunits A through H and catalyzes the hydrolysis of ATP which is associated with the pumping of protons into a compartment via the membrane-bound Vo complex. The Vo complex includes three integral proteins, named subunits a, c, c”, and one hydrophilic subunit d [44]. In tomato, two isoforms of the subunits A (A1 and A2) were isolated. Whereas VHA-A2 isoform was found to be specifically expressed in roots, VHA-A1 isoform was ubiquitously expressed in all tissues and up-regulated by salinity stress [45]. The analysis of expression of the *VHA-A1* isoform in the elongating zone of the tomato hypocotyl during BL-induced de-etioation revealed the accumulation of *VHA-A1* transcripts during the time-course of the experiment (Fig. 6b). When tomato seedlings were grown in BL on a medium containing varying concentrations of bafilomycin A1, a specific inhibitor of V-ATPases, the length of hypocotyl increased with increased concentration of bafilomycin A1 (Fig. 6c). These results indicated that in tomato, like in barley, BL induces accumulation of V-ATPase as well as its activation [12]. Both events appear to be required to trigger the restriction of cell expansion occurring during de-etiolation. To conclude, we found a strong evidence that V-ATPases play a role during BL-mediated inhibition of hypocotyl growth. It nevertheless would be interesting to verify if V-ATPase participates in elaborating the turgor pressure required for cell expansion or if it contributes to cell wall integrity.

## Conclusion

BL-induced de-etiolation is a sequential process depending first on PHOT1 during the first 30–40 min of exposure to BL, with CRY1 being later responsible for the establishment of the steady-state growth rate. Whereas CRY1-mediated de-etiolation has been characterized at the molecular level [46], no information had been available concerning the PHOT1-mediated phase of de-etiolation.

Our analysis contributes to the understanding of PHOT1-mediated de-etiolation in plants and more particularly in important crop species. Using a subtracted cDNA library, we were able to identify 152 genes quickly up-regulated by BL. Their annotation revealed deep changes in chromatin modelling, transcription, and translation, but also in cellular processes and signaling such as cell wall integrity/synthesis, cytoskeleton, and trafficking/secretion. By using a high-throughput RNAseq method we could obtain more precise information concerning genes differentially expressed during PHOT1-mediated de-etiolation. We are currently developing such analysis including also a tomato mutant depleted of PHOT1 generated in our laboratory by artificial microRNA. Nevertheless, the current study already opens the doors toward processes upon which to focus our attention, notably chromatin modelling and the potential role of histone 2B, as well as the involvement of V-ATPase in either generating appropriate turgor pressure or participating in cell wall integrity/synthesis. It is also noteworthy that an array of sequences encodes for protein of unknown function and represents a pool of proteins with novel functions in PHOT1-mediated de-etiolation.

## Availability of data and material

The dataset supporting the conclusions of this article is included within the article and its Additional files 1 and 2.

## Additional files

Additional file 1: Table S1. Gene ontology and functional annotation of the expressed sequence tags (ESTs) up-regulated by 30 min of exposure to blue light. (XLSX 68 kb) Additional file 2: Nucleic acid sequences of the 168 ESTs found to be putatively up-regulated 30 min after exposure to BL. (DOCX 45 kb)

## References

[1] Quail PH (2002). Photosensory perception and signalling in plant cells: new paradigms?. Curr Opin Cell Biol.

[2] Kami C, Lorrain S, Hornitschek P, Fankhauser C (2010). Light-regulated plant growth and development. Curr Top Dev Biol.

[3] Schumacher K, Vafeados D, McCarthy M, Sze H, Wilkins T, Chory J (1999). The *Arabidopsis det3* mutant reveals a central role for the vacuolar H^+^-ATPase in plant growth and development. Genes Dev.

[4] Cosgrove DJ (1981). Rapid suppression of growth by blue light: occurrence, time course, and general characteristics. Plant Physiol.

[5] Cashmore AR, Jarillo JA, Wu Y-J, Liu D (1999). Cryptochromes: blue light receptors for plants and animals. Science.

[6] Lin C (2000). Plant blue-light receptors. Trends Plant Sci.

[7] Parks BM, Cho MH, Spalding EP (1998). Two genetically separable phases of growth inhibition induced by blue light in Arabidopsis seedlings. Plant Physiol.

[8] Folta KM, Spalding EP (2001). Unexpected roles for cryptochrome 2 and phototropin revealed by high-resolution analysis of blue light-mediated hypocotyl growth inhibition. Plant J.

[9] Kinoshita T, Emi T, Tominaga M, Sakamoto K, Shigenaga A, Doi M, Shimazaki K-I (2003). Blue-light and phosphorylation-dependent binding of a 14-3-3 protein to phototropins in stomatal guard cells of broad bean. Plant Physiol.

[10] Folta KM, Leig EJ, Durham T, Spalding EP (2003). Primary inhibition of hypocotyl growth and phototropism depend differently on phototropin-mediated increases in cytoplasmic calcium induced by blue light. Plant Physiol.

[11] Shinkle JR, Jones RL (1988). Inhibition of stem elongation in *Cucumis* seedlings by blue light requires calcium. Plant Physiol.

[12] Klychnikov OI, Li KW, Lill H, de Boer AH (2007). The V-ATPase from etiolated barley (*Hordeum vulgare* L.) shoots is activated by blue light and interacts with 14-3-3 proteins. J Exp Bot.

[13] Bergougnoux V (2014). The history of tomato: from domestication to biopharming. Biotechnol Adv.

[14] Bergougnoux V, Zalabák D, Jandová M, Novák O, Wiese-Klinkenberg A, Fellner M (2012). Effect of blue light on endogenous isopentenyladenine and endoreduplication during photomorphogenesis and de-etiolation of tomato (*Solanum lycopersicum* L.) seedlings. PLoS One.

[15] Diatchenko L, Lau YF, Campbell AP, Chenchik A, Moqadam F, Huang B, Lukyanov S, Lukyanov K, Gurskaya N, Sverdlov ED, Siebert PD (1996). Suppression subtractive hybridization: a method for generating differentially regulated or tissue-specific cDNA probes and libraries. Proc Natl Acad Sci U.S.A..

[16] Gulyani V, Khurana P (2011). Identification and expression profiling of drought-regulated genes in mulberry (*Morus* sp.) by suppression subtractive hybridization of susceptible and tolerant cultivars. Tree Genet Genomes.

[17] Guo W-L, Chen R-G, Gong Z-H, Yin Y-X, Li D-W (2013). Suppression Subtractive Hybridization Analysis of Genes Regulated by Application of Exogenous Abscisic Acid in Pepper Plant (*Capsicum annuum* L.) Leaves under Chilling Stress. PLoS One.

[18] Zhou GF, Liu YZ, Sheng O, Wei QJ, Yang CQ, Peng SA (2015). Transcription profiles of boron-deficiency-responsive genes in citrus rootstock root by suppression subtractive hybridization and cDNA microarray. Front Plant Sci.

[19] Bergougnoux V, Hlaváčková V, Plotzová R, Novák O, Fellner M (2009). The *7B-1* mutation in tomato (*Solanum lycopersic*um L.) confers a blue light-specific lower sensitivity to coronatine, a toxin produced by *Pseudomonas syringae* pv. *tomato*. J Exp Bot.

[20] Miao H, Qin Y, da Silva JA T, Ye Z, Hu G (2013). Identification of differentially expressed genes in pistils from self-incompatible *Citrus reticulata* by suppression subtractive hybridization. Mol Biol Rep.

[21] Conesa A, Götz S, García-Gómez JM, Terol J, Talón M, Robles M (2005). Blast2GO: a universal tool for annotation, visualization and analysis in functional genomics research. Bioinformatics.

[22] Lohse M, Nagel A, Herter T, May P, Schroda M, Zrenner R, Tohge T, Fernie AR, Stitt M, Usadel B (2014). Mercator: a fast and simple web server genome scale functional annotation of plant sequence data. Plant Cell Environ.

[23] Klie S, Nikoloski Z (2012). The choice between MapMan and gene ontology for automated gene function prediction in plant science. Front Genet.

[24] Dekkers BJW, Willems L, Bassel GW, van Bolderen-Veldkamp RP, Ligterink W, Hilhorst HWM, Bentsink L (2012). Identification of reference genes for RT-qPCR expression analysis in Arabidopsis and tomato seeds. Plant Cell Physiol.

[25] Pfaffl MW (2001). A new mathematical model for relative quantification in real-time RT-PCR. Nucleic Acids Res.

[26] Fisher AJ, Franklin KA (2011). Chromatin remodeling in plant light signalling. Physiol Plantarum.

[27] Tessadori F, Schulkes RK, van Dreil R, Fransz P (2007). Light-regulated large-scale reorganization of chromatin during the floral transition in Arabidopsis. Plant J.

[28] Benvenuto G, Formiggini F, Laflamme P, Malakhov M, Bowler C (2002). The photomorphogenesis regulator DET1 binds the amino-terminal tail of histone H2B in a nucleosome context. Curr Biol.

[29] Carpita NC, Gibeaut DM (1993). Structural models of primary cell walls in flowering plants: consistency of molecular structure with the physical properties of the walls during growth. Plant J.

[30] Van Volkenburgh E, Schmidt MG, Cleland RE (1985). Loss of capacity for acid-induced wall loosening as the principal cause of the cessation of cell enlargement in light-grown bean leaves. Planta.

[31] Kutschera U (1996). Cessation of cell elongation in rye coleoptiles is accompanied by a loss of cell-wall plasticity. J Exp Bot.

[32] Pauly M, Qin Q, Greene H, Albersheim P, Darvill A, York WS (2001). Changes in the structure of xyloglucan during cell elongation. Planta.

[33] Baumann MJ, Eklöf JM, Michel G, Kallas ÅM, Teeri TT, Czjzek M, Ill HB (2007). Structural evidence for the evolution of xyloglucanase activity from xyloglucan *endo*-transglycosylases: biological implications for cell wall metabolism. Plant Cell.

[34] Miedes E, Herbers K, Sonnewald U, Lorences EP (2010). Overexpression of a cell wall enzyme reduces xyloglucan depolymerization and softening of transgenic tomato fruits. J Agric Food Chem.

[35] Miedes E, Zarra I, Hoson T, Herbers K, Sonnewald U, Lorences EP (2011). Xyloglucan endotransglucosylase and cell wall extensibility. J Plant Physiol.

[36] Nishikubo N, Takahashi J, Roos AA, Derba-Maceluch M, Piens K, Brumer H, Teeri TT, Stålbrand H, Mellerowicz EJ (2011). Xyloglucan *endo*-transglycosylase-mediated xyloglucan rearrangements in developing wood of hybrid aspen. Plant Physiol.

[37] Zhao Q, Yuan S, Wang X, Zhang Y, Zhu H, Lu C (2008). Restoration of mature etiolated cucumber hypocotyl cell wall susceptibility to expansion by pretreatment with fungal pectinases and EGTA in vitro. Plant Physiol.

[38] Gou J-Y, Miller LM, Hou G, Yu X-H, Chen X-Y, Liu C-J (2012). Acetylesterase-mediated deacetylation of pectin impairs cell elongation, pollen germination, and plant reproduction. Plant Cell.

[39] Gendreau E, Traas J, Desnos T, Grandjean O, Caboche M, Höfte H (1997). Cellular basis of hypocotyl growth in Arabidopsis thaliana. Plant Physiol.

[40] Cosgrove DJ (2005). Growth of the plant cell wall. Nat Rev Mol Cell Bio.

[41] Perrot-Rechenmann C (2010). Cellular responses to auxin: division versus expansion. Cold Spring Harbor Perspect Biol.

[42] Dettmer J, Hong-Hermesdorf A, Stierhof Y-D, Schumacher K (2006). H^+^-ATPase activity is required for endocytic and secretory trafficking in *Arabidopsis*. Plant Cell.

[43] Brüx A, Liu T-Y, Krebs M, Stierhof Y-D, Lohmann JU, Miersch O, Wasternack C, Schumacher K (2008). Reduced V-ATPase activity in the *trans*-Golgi network causes oxylipin-dependent hypocotyl growth inhibition in *Arabidopsis*. Plant Cell.

[44] Padmanaban S, Lin X, Perera I, Kawamura Y, Sze H (2004). Differential expression of vacuolar H^+^-ATPase subunit c genes in tissues active in membrane trafficking and their roles in plant growth as revealed by RNAi. Plant Physiol.

[45] Bageshwar UK, Taneja-Bageshwar S, Moharram H, Binzel ML (2005). Two isoforms of the A subunit of the vacuolar H^+^-ATPase in *Lycopersicum esculentum*: highly similar proteins but divergent patterns of tissue localization. Planta.

[46] Folta KM, Pontin MA, Karlin-Neumann G, Bottini R, Spalding EP (2003). Genomic and physiological studies of early cryptochrome 1 action demonstrate roles for auxin and gibberellin in the control of hypocotyl growth by blue light. Plant J.

