# Supplementary material for: A subtracted cDNA library identifies genes up-regulated during PHOT1-mediated early step of de-etiolation in tomato (Solanum lycopersicum L.)
Source: BMC Genomics. 2016 Apr 18;17:291. doi: 10.1186/s12864-016-2613-6 (PMC4835860; doi:10.1186/s12864-016-2613-6)
Supplement: Additional file 2: — Nucleic acid sequences of the 168 ESTs found to be putatively up-regulated 30 min after exposure to BL. (DOCX 45 kb) [file 12864_2016_2613_MOESM2_ESM.docx]

>12

ACCTCGCTTTATCAGTGCAATAGTTATAAACAGTGTATTTTTGACGAACCCAACGAAGTCTCCTATACTGTAATGCATCTAAATCTTGGAAGGCCTTTTGATCCCACCATTTCATGCCTTTAGTGTTACAAACTTGGACTTCTTGTGGCGTGGCAGCTTCACATCCATCCACGTGGAACGATGTGTATGACGCGGTGAATGGGGCGTTGGCCCAATTGGTTTTCTCAAGCCCACCTCTTGTGGCCCAATCATCTGCGTCCCATAGACTCGAGTATATCTTCATGGGCTGATTGAATGGAAATTTCACACCAAGATCTTTCGAATTTTTGAATGCTCTAATTGGAACGTCGTCCACAAAGATCACAATGAGGTATGTATTCCAAAGAACAGAATAAGAATGGTAGCCCTTGGTTGGATCAAACCAAAGATATATTCTCT

>13

ACCAGTCCACTTCTTCCTTAGTGTTCACTCCCTCGATCTGAATCAACGAAGTGTTGGGATACTGGTTCGATTTCGACCTTTTGTATCCAAGAACAGTCCCTCTAACGTAGAGCCTAATGCGTTCTCCTTGGCGTCCCTTCACCATTTTCGC

>15

ATGGATGCCGCCACTCACTTCCCGATGGTCTCATGAGGGCTACTGATGTTATGATTGCTGGAAAGGGTGCCCTTGTTGCTGGTTATGGAGATGTCGGCAAGGGATGTGCTGCTGCCATGAAACAAGCTGGTGCACGCGTTATTGTGACTGAGATTGATCCAATCTGTGCTCTCCAGGCTACCATGGAAGGTCTCCAGGTTCTTCCTCTTGAGGATGTTGTTTCTGAGGTTGATATCTTTGTGACCACCACTGGTAACAAGGACATCATCATGGTTGACCACATGAGGAAGATGAAGAACAATGCCATCGTCTGCAACATTGGTCACTTTGACAATGAAATCGACATGCATGGTCTTGAGACCTTCCCTGGTGTGAAGAGGATCACAATCAAGCCTCAAACCGACAGATGGGTTTTCCCAGACACCAACAGTGGCATCATTGTGTTGGCCGAGGGTCGTCTCATGAACTTGGGATGTGCCACAGGACACCCCAGTTTTGTGATGTCTTGCTCTTTCACTAACCAAGTCATTGCCCAACTCGAGTTGTGGAATGAGAAGAGCAGTGGTAAATACGAGAAGAAGGTATACGTTTTGCCAAAGCACCTTGACGAGAAGGTTGCTGCCCTTCATCTTGGAAAGCTCGGAGCCAAACTTACCAAACTTACCAAGGATCAAGCCGACTACATTAGCGT

>21

ACAATGAAGTGGAACTCTCTGCTCTTGGGATGGCTATTTCGACAGTGGTTACTATTGCCGAAATTCTCAAGAACAATGGATTTGCTGTTGAGAAGAAGATCAGGACACTCACCGTGGACATGAGGGATGAACCAGGAGCCCGACCGATACCAAAAGCAAAGATTGAAATAGTGCTGGGCAAGACTGAGAAGTTTGATGAGTTAATGGCTGCAGAAGCCGAGCAAAACGGGGACAATGAGGAGCAGCAGAACTGATTATTTCCAAATCTGTTCTCTTCCTGACTATTGAAGTTATTCCAATCTATACTGCATTCAGCTGTTGGGTTCTCTATGGCCTTTAATTTGATCTTTTGGAAAGTTTTTTTTTAAAAAAAAAGCAGCATGGTTTACTTTAAAATTCATCATTTGTTGGATGTGTATGCCTTTTTTTTTTTTTAACCTTGCTTATCTAAGGTGGTGTTGAATGTAAACATCTTGCTGCTATCTTCACTGTAAGTGATGTGGTAATAGTTTATGCCTGGTAGTTAAAAAATTGAACAATTGGATATGTGGGTTGACATTTCTGTCATTACCTAATTAAGCATCATTAGT

>27

GGGTCGTGTGATCAGAGCACAACGTAAGGGAGCAGGCTCCGTCTTCAAATCTCACACTCATCACCGCAAGGGACCTGCAAGGTTCCGTTCACTTGATTTCGGTGAACGTAATGGTTATCTCAAAGGTGTTGTTACAGAAATTATTCATGATCCAGGTAGGGGTGCACCATTGGCAAGGATGACATTCCGTCATCCTTTCCGTTACAAGCATCAGAAGGAGTTGTTCGTTGCTGCAGAAGGGATGTATACTGGTCAGTTTATTTACTGTGGGAAAAAAGCTAATCTAATGGTTGGTAATGTGCTTGCACTCAGATCTATCCCTGAAGGAGCTGTTGTTTGTAATGTTGAGCATAAAGTTGGTGACCGTGGTGTTTTTGCTAGATGCTCTGGTGATTATGCTATTGTTATCAGTCATAACCCTGATAACGGAACAACTAGGATTAAGCTTCCATCTGGATCCAAGAAGATTGTGCCTAGTGGATGTCGAGCCATGATTGGTCAGGTTGCTGGTGGAGGAAGAACTGAGAAACCAATGCTTAAAGCTGGTAACGCATATCACAAATACCGTGTTAAGAGGAACTGCTGGCCTAAGGTTCGTGGTGTTGCTATGAATCCTGTGGAGCATCCTCATGGTGGTGGTAACCATCAACATATTGGTCATGCCAGT

>3

ACTTAGAACATGATGCTTGTTGAAGCTTGAGTGATGAGCTGAAACCTATATTCCTTCTGACAAGGTCAAATACCAATAGGTTCTCTTCCATTTGATGTCCTCCTATAACAATCGAAGGTTCCCATGTTTGGTCTTGTCCCACAAATGCTAAACACATAACATCCTTGCTAACTTGT

>32

ACTATGCTATGTTGGCAAAGGTTGGAGTTCGCCACTACAACGGAAACAACGTAGACTTGGGGACTGCCTGTGGTAAATACTTCAGAGTTTGTTGCCTCAGTATTATTGACCCAGGTGATTCTGATATTATTAAAAGCTTGCCTTCTGACCAGTGAAAGAGGCATTTGATTTGATGCTGTTTTGGATCCTATTCAGAAGTTGCCCGATTGTTATTAAGAAAAAGTAGCTACAATTGATGAGTTTGACAACTTCATTTATCACTTGTTGCAAGAAAGTTTTGATGTTGAATCTGATTTTAACAATTTTCTTGAGTTATCTTTCCATCTACTTTGTAAAGATAACTCAAGAAAAT

>33

ACCCTAATTGGAACCATGGCACCATTGTCATTGCTATACTCAACAGTAACTTGGGTCTTGCCATCAGGCCTCAACCAGGCGCAGGTGCCATTCTTGCGGACCTCTGTAAGACGGGCACCAAGTTTAGTTGCAAGCACGCGACTGAGAGGCATTAATTCAGGGGTCTCATCTGTTGCATAGCCAAACATGTGGCCCTGGTCACCAGCACCAATCTCCTCGGGGCGTTTGGTCAGATGGCCGTGGACACCTTGAGCAATATCAGGACTTTGCTGCTCAATGTAAACAAGGACCTTGCAGTTGTCAGCATCAAGACCAACATCATCAGAAGCAAATCCAATATTACGGCATGTGTCACGCACAATCTTCTCATAGTCTACAATAGCCTTGGTTGTGATCTCACCAAAGACCATGACCAAGTTGGTCTTGGTGCAAGTTTCACATGCAACTTTGCTCTCGGGATCTTGCTCAAGGCAGGCATCAAGAACTGCATCAGAGATCTGATCACAGAGCTTGTCTGGGTGACCCTCGTTCACAGACTCGGAGGTGAATAAGAAAGTTTCCATTCTCAAGAAACAGGAAAGTGAAGAACCCAACGGCGAACAAAGAGAAAAAGAGGATTATAAATGTCGAAAAGGGGCAATCTTTTTACCCCTTGAGAGAAATAACCTCTTTGTATTTTTGGTTCGCA

>35

CAAGGCTCGCTTCGCGGGCCGATCCGGGCGGAAGACATTGTCAGGTGGGGAGTTTGGCTGGGGCGGCACATCTGTTAAAAGATAACGCAGGTGTCCTAAGATGAGCTCAACGAGAACAGAAATCTCGTGTGGAACAGAAGGGTAAAAACTCGTTTGATTCTGATTTCCAGT

>38

GGTAGAGCCAAGTCATTTCCCCATGCTTGCTGACTGGAAGAGGGAAAACACAATGGAGGATATACTGATGCAGTTGAAAAAGGAAATGACGTCTCCCCAAAATCGAAAACTAGCTCAGCCTTCTGATGGCAATGAAGAGGGCCGGGTGGATCAAAAAGGCCTAGTTGTGAAATGCTGCATTATGTAAGTTTGATAAAATACTGAAAGATAATAATGTTTGTATATAACCTTAAAAAAATACGTAGACGATTCCATAGTATCGTAGCATGTATAACTATGTGTATGTTTTGTATTTCTTATTGAATCCTCCTTAAATTATGGGATGGATTGATGTTTAATAACCAATTATCCAACACTCTGAAAAAAAAAAAAAAAAAAAAAAAAAAAAGCTTGT

>4

ACTTGGGGACCATATTCTGCATCTTGTGCTTTTATTGCTCTCTTGGTTTACAGATCTTTGGTGGGATTGTCAACGCTGGAAACCCCAATTTAGCCCAAACTGAGCTTTCTGATAATGACTATTTGCTTTTTAACTTCAATGACTATCCAAGTGGCATGGCCACAGTTTTCAACATATTGGTGATGGGAAATTGGCAAGTGTGGATGCAGAGCTACAAGGAACTAACGGGGACTGCCTGGACTTATGTCTACTTCGTCGGCTTCTATCTTATCTCAGTTTTGTGGCTGTTGAATTTGATTGTAGCATTTGTCTTGGAAGCTTTCCAAGCAGAAATGGATCTAGAGGCTGCAGCAAACTGTGCGGATGGTGATGACAAGGAATCAAGAAGTGAGCGGAGACGTAATGTTGGCACTAAGACTCGGAGCCAGCGAGTAGATTTCCTCCTGCATCACATGCTGAGTTCTGAACTAACAGAATGCTCCCATGACGATCCATAAGTTTACCCCTCCTTTTCTTCATGCTGCCTTATGCAGTCCTGTGAACATCAGAAGTTGAGTTATTTTGGTGGTGAAGCCAAATTGGCAATGT

>40

GTCCAGGAAGCAAGGCAAGCTGCTGAGAAAGCTATTAATGTGCCAAACGTGGACGAAAGAGCAAAACGAGCTGTTACTTCTGCCAACAAGTCCGCCACTGCAGCAAGAGTAGCAGCTGTGAAAGCTGTCCAAAACCAGATGCATCAGAATGGAGACAGTTGCCATTCACCGTTATCAGTTGTGTAAACCCCTCAGTTGGCTGAGTCTATGGTTTTACACTCTCTCATCGGTCTTGTTTTTAGATACAGAGACTGAGACAACCAGGGACCAACTTGGAACCACAACAGGCAACGAGCTAATTGAGGAGCAAGTTTATCGTGTCCCTTCCATTAGGCCTTTACTAATATGCACATATAGTGCGCCCTCACATAGGAGCTAAGACCGCAACCACAACCAAAAAAATAAAAGACTGCAGATGTAAGTAGGTTGTTCGTCGTTTCTTTCTGTTGTTGCTGTCCAGTAAGTGTAGATAGTTTCCTGTTTTCTGTGTTTTGCCATAAGAGAATGGCCAAGTTTCTAATCTTGTATCTCAAAGGAAGATCACCTCTGCTGTGTTATGTTGTATAAAGTAATAGTAACTAAACATATGCTGTATTAACAAATGACTATAAATTTTGTTTCATCAGCAAAAAAAAAAAAAAAAAAAAAAAAAAAAGCTTGTACGATATTTTTGGCTTTCCTGGTCTTTTTACCCTTGAGAGATTTTGATTGACTTGTATTTGAGAGGTATATGAAGATTTTTTGCTACTTCGAT

>47

ACTGGCATGACCAATATGTTGATGGTTACCACCACCATGAGGATGCTCCACAGGATTCATAGCAACACCACGAACCTCAGGCCAGCAGTTCCTCTTAACACGGTATTTGTGATATGCGTTACCAGCTTTAAGCATTGGTTTCTCAGTTCTTCCTCCACCAGCAACCTGACCAATCATGGCTCGACATCCACTAGGCACAATCTTCTTGGATCCAGATGGAAGCTTAATCCTAGTTGTTCCGTTATCAGGGTTATGACTGATAACAATAGCATAATCACCAGAGCATCTAGCAAAAACACCACGGTCACCAACTTTATGCTCAACATTACAAACAACAGCTCCTTCAGGGATAGATCTGAGTGCAAGCACATTACCAACCATTAGATTAGCTTTTTTCTCACAGTAAATAAACTGACCAGTATACATCCCTTCTGCAGCAACGAACAACTCCTTCTGATGCTTGTAACGGAAAGGATGACGGAATGTCATCCTTGCCAATGGTGCACCCCTACCTGGATCATGAATAATTTCTGTAACAACACCTTTGAGATAACCATTACGTTCACCGAAATCAAGTGAACGGAACCTTGCAGGTCCCTTGCGGTGATGAGTGTGAGATTTGAAGACGGAGCCTGCTCCCTTACGTTGTGCTCTGATCACACGACCC

>48

ACCGCTCTTAGCCATGTCAATAATCTCATCGTTGGTGTTACCAAGGGATACAGATACAAGATGCGTTTTGTATATGCTCACTTTCCGATCAATGCCTCCATCACCGGAGGTAACAAGTCCATTGAGATCCGTAACTTCCTTGGCGAGAAAAAGGTTAGGAAAGTTGACATGCTTGATGGAGTAACAGTTGTTCGATCTGAGAAGGTTAAGGACGAGCTTGTGTTGGATGGAAATGACATTGAGCTTGTTTCTCGCTCTGCTGCCCTCATTAACCAAAAATGCCATGTGAAGAACAAGGATATCCGAAAGTTTCTTGATGGTATCTATGTCAGCGAGAAGGGTAAAATTGTCGAAGAAGAATGAGTATTTTAGAAGTAGGCATGTTGGTGATGGACCATATCCCAAATCCTCGTTTCACCATTTCTTTCAAGTTTTTTTGGAGACCATATATTACAGACTTGTTTGAATTTCAGTTTTCTAGTTCTTGTGTTGAAATTTTGTTGACAAAGTGGCCTTGGTGGTTCTTTCAATTACACCAAATGTTTCTGTTCTCGTTAAAAAAAAAAAAAAAAAAAAAAAAAAAAAAAAAAAAAAAAAAAAAAAAAAAAAA

>52

CGTCATCTAATTAGTGACGCGCATGAATGGATTAACGAGATTCCCACTGTCCCTGTCTACTATCCAGCGAAACCACAGTCAAGGGAACGGGCTTGGCAGAATCAGCGGGGAAAGAAGACCCTGTTGAGCTTGACTCTAGTCCGACTTTGTGAAATGACTTGAGAGGTGTAGTATAAGTGGGAGCCGAAAGGCGAAAGTGAAATACCACTACTTTTAACGTTATTTTACCTATTCCGTGAATCGGAAGCGGGGCACTGCCCCTCTTTTTGGACCCAAGGCTCGCTTCGCGGGCCGATCCGGGCGGAAGACATTGTCAGGTGGGGAGTTTGGCTGGGGCGGCACATCTGTTAAAAGATAACGCAGGTGTCCTAAGATGAGCTCAACGAGAACGGAAATCTCGTGTGGAACAGAAGGGTAAAAGCTCGTTTGATTCTGATTTCCAGT

>54

GCAATGGAAGCCTCAGTTTCTCTTCAGAAATACTCGGGTTCAAGCGCCATTTCTAGTGCTGACCTTTTTGGTCAAGATGATGGGGCAGCTTATGAAATAAGTGCCGGCGATTTAATAAATAGGATCTCTTTCCAGGCACAACAGGATATGTCGTCTATCAAAAATATTGCTGGAGAAACTGGGAAGAAGCTGTCCTCCTTAGCATCCAATTTGATATCTGATCTTCAAGATAGAATCCTGTGAGGAGAGACTTTTGTGTGTTATAAATGAAATGAAGATGACAGTATCTAAATATTGCTTGTAGAGGTGAAAAAAGT

>55

ACAGATAATTAAATGGACCAGTGGGGAGGTCGCTTATGGCTACGCCAGTCAAAGCTTTTATGTAGTTTGTATCATCAATATGCATCCACATTACATATTTACCTGTCTTCTCATTGTAAATTACTTTTGGCCTCTCCAGT

>56

ACTGGAAATCAGAATCAAACGAGCTTTTACCCTTCTGTTCCACACGAGATTTCTGTTCTCGTTGAGCTCATCTTAGGACTCCTGCGTTATCTTTTAACAGATGTGCCGCCCCAGCCAAACTCCCCACCTGACAATGTCTTCCGCCCGGATCGGCCCGCGAAGCGAGCCTTGGGTCCAAAAAGAGGGGCAGTGCCCCGCTTCCGATTCACGGAATAAGTAAAATAACGTTAAAAGTAGTGGTATTTCACTTTCGCCTTTCGGCTCCCACTTATACTACACCTCTCAAGTCATTTCACAAAGTCGGACTAGAGTCAAGCTCAACAGGGTCTTCTTTCCCCGCTGATTCTGCCAAGCCCGTTCCCTTGGCTGTGGTTTCGCTGGATAGTAGACAGGGACAGTGGGAATCTCGTTAATCCATTCATGCGCGTCACTAATTAGATGACGAGGCATTTGGCTACCTTAAGAGAGTCATAGTTACTCCCGCCGTTTACCCGCGCTTGGTTGAATTTCTTCACTTTGACATTCAGAGCACTGGGCAGAAATCACATTGCGTAAACATCCGTTGGGACCATCGCAATGCTTTGTTTTAATTAAAC

>65

TAGCTGCTAAGGACTGGACAAATGTTGCCAAGAAAGGCTCTATTGATCTTATGGAACTTGAACTGAAGAAGATGTATGAACATGTTCAAAGCATCCATGACGAGATGTTTTATCTACGTGAAAGGGAGGAAGAAATGCAAGAACTTAACAGATCAACCAACTCCAAAATGGCCTGGATGACTGGACTGTCAATCTGTGTATGCTTATCTGTAGCAGGATTGCAATTGTGGCATTTGAAAACCTTTTTCGAAAAGAAGAAGCTAATCTAATGTTATTCTCATATTTTCACTACCCTCACGTCCGAGTAGAATCTTTTATAGACAACACAATAGTTAGAAGGAAAGTTGTGTTACATGTACCTCGGCCGCGACCACGCT

>68

GGACTTTGGGATGGGCCGGCCGGTCCGCCCTAGGTGTGCACCGGTCGTCTCGTCCCTTCTGTCGGCGATGCGCTCCTGGCCTTAATTGGCCGGGTCGTGCCTCCGGCGCTGTTACTTTGAAGAAATTAGAGTGCTCAAAGCAAGCCTACGCTCTGTATACATTAGCATGGGATAACATTATAGGATTTCGGTCCTATTACGTTGGCCTTCGGGATCGGAGTAATGCAAGGAAAAATGGGAGAACATCAACAAGT

>71

CTTATGTTGAGCCCAATAAGATCAAGAAACTTACTGGGGTTAAAGCTAAAGAGCTTCTAATGTGGCTAACACTTAATGAAATTTCTGTTGATGAGCCGTCTACTGGGAAAATCCATTTCAAGACCCCAACTGGTCTTGCTAGAACTTTCCCTGTTTCAGCCTTTGAACTTGATCTCCCTAAAAAGGAAGTCAAAGAAGAAGCCAAAGAAGTGAATGCTGCTGCTGTTGAAGTGAAAGAGGTTTAAAGAAAAAAAAAAGTTTTATTTTGATTAAAAAGTTGTTGTTTTACTACTACTTAAGTTGAGGATGATGATTATCTTGATGT

>79

ACCCTGGACGACAAAATTGCTGAGACCACCATCACGCTTTAGTGTTACATTTATCTTCTCTTCAACAGTCAACGTGACGGGATCAGTTAGTGGAGCAGCAGCTGGTTTGGCCTGACCAACGCTTGGCCTCACATCCTCGACAATTACCTCACCCTCAGCTTTTAGGGATTCCAGAAATTGGTTGGTCTTCTGTGTTTTACCCAGTTGCATACCAAGACCTTTCGGTGGAGCAGTAGCAGATGCAGCTGGACGACCCTTGGATTTGGTGGAAAATGTGTCCACATCAGGGGGTAGAGCAAATCCACCAGAACCATTGCCTCCTATGTTGGATATGCCTGAGTCGCTGCCAAAGCCAGTATCAATTCTTCCAGAACTCATGGATTGCAGTGACATGAAACCTCCTTTGTCACCTCTATTCCTCTCAATCTTGCTTTTGTCAATTTCACTGGCTTTACGCTTCATGACATCCTTAGTTTCATTTATCTTGTTCTGTAAGACTAACTTGTGTAATCTCTCCTCGTGACTCTCCATTTCACAGT

>8

CCCAACGGATGTTTACGCAATGTGATTTCTGCCCAGTGCTCTGAATGTCAAAGTGAAGAAATTCAACCAAGCGCGGGTAAACGGCGGGAGTAACTATGACTCTCTTAAGGTAGCCAAATGCCTCGTCATCTAATTAGTGACGCGCATGAATGGATTAACGAGATTCCCACTGTCCCTGTCTACTATCCAGCGAAACCACAGCCAAGGGAACGGGCTTGGCAGAATCAGCGGGGAAAGAAGACCCTGTTGAGCTTGACTCTAGTCCGACTTTGTGAAATGACTTGAGAGGTGTAGTATAAGTGGGAGCCGAAAGGCGACAGTGAAATACCACTACTTTTAACGTTATTTTACTTATTCCGTGAATCGGAAGCGGGGCACTGCCCCTCTTTTTGGACCCAAGGCTCGCTTCGCGGGCCGATCCGGGCGGAAGACATTGTCAGGTGGGGAGTTTGGCTGGGGCGGCACATCTGTTAAAAGATAACGCAGGTGTCCTAAGATGAGCTCAACGAGAACAGAAATCTCGTGTGGAACAGAAGGGTAAAAGCTCGTTTGATTCTGATTTCCAGT

>80

AGATTGGCTTACAACTATGACTATCTTGGGATGATACTGCTCTTGCTGGGGTTGAGAGAACTATTCAGAACAAACCGAAGGCAGTAGAAGATAAAACTCTTCTCACTTAAATAAGGTTAATCCGTCATAAAAGTGTCTAGAAAACTTAATGTCAGTGAGAGAGCAAAAACTATTTGAGGATTATTATTTATCAATTGAGGTAGTCTTCTTTGTTTCTACTTGCTATGTTATAAATCAATATTCATAAAAATTACTATTTTTCTATAGAAAAAAAAAAAAAAAAAAAAAAAAAAAAGCTGT

>82

ACTTGGGGACCATATTCTGCATCTTGTGCTTTTATTGCTCTCTTGGTTTACAGATCTTTGGTGGGATTGTCAACGCTGGAAACCCCAATTTAGCCCAAACTGAGCTTTCTGATAATGACTATTTGCTTTTTAACTTCAATGACTATCCAAGTGGCATGGCCACAGTTTTCAACATATTGGTGATGGGAAATTGGCAAGTGTGGATGCAGAGCTACAAGGAACTAACGGGGACTGCCTGGACTTATGTCTACTTCGTCGGCTTCTATCTTATCTCAGTTTTGTGGCTGTTGAATTTGATTGTAGCATTTGTCTTGGAAGCTTTCCAAGCAGAAATGGATCTAGAGGCTGCAGCAAACTGTGCGGATGGTGATGACAAGGAATCAAGAAGTGAGCGGAGACGTAATGTTGGCACTAAGACTCGGAGCCAGCGAGTAGATTTCCTCCTGCATCACATGCTGAGTTCTGAACTAACAGAATGCTCCCATGACGATCCATAAGTTTACCCCTCCTTTTCTTCATGCTGCCTTATGCAGTCCTGTGAACATCAGAAGTTGAGTTATTTTGGTGGTGAAGCCAAATTGGCAATGT

>9

ACTGGGATTAACCCCAAAACAAAGTCATATTACCTATTTGATGGCTATGCTCATCTCTCATCTGGACTTGCTTGTGGTCTTGCTGGTCTTTCTGCTGGAATGGCTATTGGTATTGTTGGAGATGCTGGTGTTGGGGCTAATGCACAACAACCCAAGCTTTTTGTTGGGATGATCCTCATTCTCATTTTCGCTGAAGCCTTGGCTCTTTATGGTCTTATTGTTGGCATTATCTTGTCTTCCCGAGCTGGGCAGTCTAGAGCTGAGTGAAGTTGACTCCATTCTTGCCGCACTGTATGTGAGACTTCAGAAGATCAAGACAGCTGTAGCCTAAAGTCAAAAAGTATCTATTATGTGT

>A-A3

GATGATCCATTGTATATGATCATCCTTTTTCCTATTTCCACTGGTTGGGATATATACCCAAAGCTTCAACTGGTGAACTATCCTCAATGGCCTGGTTAAGGAAATCAAGTTTTAGTTCGTGTGCTACTGTTGTTCTAGGATGCAATGATTCTAGCATGACTTTCATTTTTAGATAACATTGAATGGCCATTATTATTACGAGAGAAAGCATTTTTAAGTTCTTACTATTTCTCAGTCCAAAAAATTCTATATTCACAGACCACAGCCAGCCTTGGAACTTGGGTATCATGTTTTTACTTGGGTTAGTTTTAAATCAATTTAGTGTTTTTTTTTCCAAAAAAAAAAAAAAAAAAAAAAAA

>A-A7

GACAACAACATCATTTATTTAGAGAAAGAAGAAGCCTTTCCTAGATTGATCCGAAGGGTGACCCGTTTGGAATCGAGCCACAAAGGGTGATTATGGCTTAAATCCAAATGACCAATGAACTTAGCTGATGATAATGATTTTGGAATTGAACCTCGAAGATTATTGTATGATAAATCCAGATGTGTGAAGTACCTCGGCCGCGAC

>A-B12

GACACCAGATTCATCAGAATGGCTGCAAAATTTGGTGCGACAATTGTGCCATTTGGGGTTGTAGGAGAAGAGGATATAGCACAGTTAGTTCTCGACTATGACGACCTAAAAAGTATACCTATATTGGGTGATCGGATAAGGAGTGAGAACGAAGAGGCAGCCAGGAGGGGCTTAGCAGTCAGGGCGGACATGGACGGGGAGATTGCCAACCAAATGTTGTATATCCCTGGCCTTTTACCTAAGATACCCGGTCGTTTTTACTTCTTTTTTGGGAAACCAATTCATACAAAGGGAAGGCAAGACCTGGTGAAAGATAGAGAAAAAGCAAGAGAATTATACTTGCAGGTAAAATCTGAAGTTCAAAATAACATGAATTATTTGCTTAAGAAAAGAGAGGAGGATCCTTACCGGAACTTCATCGATCGAACCATGTATAGAGCATTTTCTGCCACTTCTGGTGATGTCCCAACATTTGATTTTTAGATGACCAGTTAGAAGTCCGATTATTATTTTTTTTGGGTAAAGGCTCTTACCCATTGTTTAATTCTATGTTGTAATTTTTTTAAAAAAATATTTATGTTCCAATATGAGCAAGCTTTCCTTCTAAAAAAAAAAAAAAAAAAAAAAAAAAAAAAAAAAA

>A-B3

CCGAGGTACCTCAGACACCTTCTCCGCGACACAGAGAATCAAGAAGGAGATCAAGAAAAACGAAATGGGAGAGAACGGATGTTGCTGCTGATATATCAAGAAGTAGTAGTAACATTGAACAGTTATTCTGGAAAAATAGGAAACCTTATAGACGAGTAGCGCGTAAACGATCACATTTTGAGAGCTTGTCTCCTTTTATTTTTGTGCAAAATAGTTGATCTGTGTAAATACTTTTTGATCGTAAGGTCTCCGACAAATGCAATAAAGCATACTCCGATCCCTTAGTAACTTAAGCTCCTTTTCACACCTGCCC

>A-B4

GCGCCGAGGTACATGATAATCAAACATTAATGAACTTCATTGTCAATCATAACAAAGTAACCAAATACTGAAATAAGACATAGCTATAGCCAATAGGCCAAGGTGTTCCTCAGTTTCCACCCTTGAAAGGAAAGGCTCTTATGATAACTTGGTGGTATAAGGCAGCAAGTGCAGCTCCAATGAATGGTCCCACCCAGAAAATCCAATGATCATCCCATGCATGTTCTTTGTTGTAGACAATGGCAGCGCCTAGGCTTCTAGCAGGGTTAATGCCGGTGCCTGTAATAGGGATTGTAGCCAAATGAACCAAGAACACTGCAAATCCAATTGGGAGAGGAGCCAGAATAGGGACATGGGAGTCTCTAGCATTTCTCTTGGCATCAGTAGCAGAGAAGACAGTGTAGACAAGAACAAAAGTGCCAATAATCTCAGCACCAAGGCCATCTCCCTTGGTGTAACCATGGGCAACAACATTGGCACCTCCACCCTTAGTCTCAAACAAAGATGGCTGGAACCCTTTGACAACACCAGCACCACAGATTGCACCAAGGCACTGCATCACAATGTAGAACACTGCCCTGGTTAAGGACAATTTCCTTGCCAGAAATAAACCAAATGTCACAGCAGGGTTAATGTGTCCACCTGATATGCCAGCAGTGCAGTAAACAAGGGCAAAAATCATACCCCCAAAAGCCCAAGCAATGCCTTGAACACCAACAGTACCTGCC

>A-B6

GAGGTGGCTCAGCAGAAACCCTAGTTCGAAAGGTAAGACAAGGTAGAACAACAACAATTGGCCGCCGCCTCTGCTTAACAAGAAGGTCGTGAAGAAGAGAGTGAAGAGGTTCATTAGACCTCAGAGTGACCGAAGAATCACTGTCAAGGAAAGCTGGCGCAGACCCAAGGGTATTGATTCTAGAGTGAGGAGAAAGTTCAAGGGATGTGTCTTGATGCCCAATATTGGATACGGGTCAGACAAGAAGACTCGCCACTATCTTCCCAATGGCTTCAAGAAGTTCGTCGTGCATAATGCTAGCGAGCTTGAGATCCTAATGATGCACAACAGAACTTACTGTGCAGAAATCGCACACAATGTTTCCACTAGGAAGAGGAAAGAGATTGTCGAGCGAGATGCCCAACTTGATGTTGTCATAACAAACAAGCTTGCTAGGTTGCACAGCCAGGAGGATGAATGAGCTATTTTGGCTTTAGATGAGATCTCTATATTGTTTTAGTGTTTGAACCTGTTCCCTTGTGTTTTGTTTAATTAATACTTCTGTTTTCCCGAAAGTGGATTTTGACACATCCTATTGCTATCAAATGCTTAAATTTTCAGATTTTAGTACCTGC

>A-C3

TGGATTATCTGACAGAATGCCAGGTGTTATTGATGTGTTGATAAACAATGGAAGACATATAGACGCTGTTAATCTAGCTTTTGCATTTGAGCTGACAGAGCAGTTTCCACCTGTTTCTCTATTGAAATCCTACTTGAATGAAGCTAGCAAAGCATCTACACCTTCCAATTCTGGAAATGCATCACCTACTGTGCAGAATGATGTCAATGAGAAAGAGTTGTCTGCACTAAAGGCTGTATTAAAATGCATTGAAGACCATAAGCTTGAGGAGCAATACCTTGTGGATCCCCTTCAGAAAAGGGTTCATCAGCTGGAGAAAGCAAAGTCCGACAAGAAAAAGGCAACTGAAGTTACAAAACCTCAATCCAAAAGACCTCGTCCCAATGGTGTCGGAAATGGCCCCCGAGTAAATAATGTCGTCACTGAGAAGAACTTCTATCCCAGAATGACTGATAGGTATCCGCAACCTGTTTATGACGGACCATATGCTTACCCTGGACCGACCAACACTCATGTCCCATCTTTCATAGGTGCTCCTGCATACAACTTCTCTCCAGGCCACGACTTTTTCGGAAATGGCTACCATTACCAGGCTCCTTACCTGCATTGATCAGGAAACTTTGATGATTATCCCTTTAACTATTAGTGTTTAACCTTGGAGTTTGCTTAATTTGCTCGTGTGGGCGAATTTAATACTGAATGTAGCCGACCTGTCTATTTCTAATCTGGTGTAAAAGCTGATATTTTGATCTTTGAATATATGCAC

>A-D3

TCCAGAAAACAATAACAAAGAGAATAGAAGTATCAATTTTGATGCCTATAGCATCATATAATAATAAATGAACCCATCAAACAAAGATCCATAACAAATTATTTGTTCTTCTTGTTTCTTCAATATTGATCCTCAGTCAATTGGTATTGCTGGAGGATCTGAGTGCAGGCAGCAAGAGGTTCGTTGGAGAGGAAACCCATGTTGTTAGCGAAACGAACATCAGAGGTCATACCATTGTTGCGGGTGAGAGCAACACGGGCACGATCACGACCAGCATTGGGGATGTTACATGATTTATCACCGCTCTCGACGAGCACAACATCACAGACATCATCACCACAGTCACGTTTAACTAAGATGTTGTATTCACCCTGTGAGTTTGTCACTCCATCGAATTGTGTATGTGATTTTGTTGGTCACCCTGTTTTTGCAACTCAACTTTAACCTTAGGATCCAGCAAGGATAACTTAGTAGCAGGGGTCTCAAATCCACATCGGCAAGTATCACAATACCTGCCC

>A-E11

TTGGCAGATGCTCCTTGATTATGATGACATGATGAAGGTTCCATATCTCAAGGCTCTCATAGAGGAGTTAACTGGTGAGGTGGAGAAGTTAAGATATGACACTGAAGGAGAGGTTTCAAACCAAGATGTGCATCTTCCAATCATTCTTCCGAAAGTTCCTGGCCGTTTTTACTTCTACTTTGGCAAGCCAATTGAAACAGCAGGGAGGAAGGAGGAACTTAAAAGCAAGGAGAAAGCACATGAATTATACTTGGAAGTGAAGTCTGAGGTTGAGAGATGTATTGATTACCTGAAGGAGAAAAGAGAGAGTGATTCGTATAGGAATATAATGGCCCGCCTGCCTTACCAGGCTAGCCATGGCTTTGATTCTGAAGTTCCCACCTTTGATCTATAGTATTATGCAAAAAAAAAAAAAAAAAAAAAAAAAA

>A-E2

AGGTACTTACTTATGGTCCTCAAGTTCACCCAATTTTTTCAGAACAACAGAAAGCAATTGAGCTTCACTAAAGGCTGGAGTTGGTGAAGGTGGCCGAAGCTCCTCATTACTGTTGGCATCAAAAGTGAATTCTTGAACAGCTTGGTCTTGACCAATTGATGGATCTGGAAATTTCTTAGTTATGCAGCATAGCAGTGAACGGAAAAGCATCACCACAGTCGTGAAGAAAGCCATAAGAAATCCTAATATCTGAGCATTAAGACCACCTGCAGGCTTTTGAGAATCTGCTGGTGGAAGTATCCCTTTGGCAATGGGGGCTTTATGAAAGGATGTTTGCTTCTTTAACACAGAATCAACCGCCTTGTCAACCATGGGAACATATTCATCATATCCTAATAAGTTTGCAGTGTAGCCAGACATTCCTATACCCTTAGCCTCTTCACGGACAGGAGTAAGCCGAAGATGCGAATAATTCCTTACAGCTTTAGGGGAACCAATATCTTCTGCCTCAGATCCCGATTCTGCCGTGGAGGTATCACTACCTTTTGACTGCATGGGGAAGCGAGGCTTAGCATAAACTACCTTTCCTTCGCTGTTTACAACTTTAACAACCTGCCTGGCACGCCGTGCTTCACCAATCATCTTTAATATTTCCGGATTTTGCCATGGCCCTTTATCTGAGCGAAGGCAACCTCCTTGATCTGCACATGTACCTGCCCG

>A-E4

GAGGTCGGCCGAGGTACCCAAGTGCCGTTGACAAATTCAAGAAGCAGTTTGCATATCTTGAAGAGCATTATGGCAAAGGAGGCGCTGCTGCTCCACCTGAGAGGCAGCACTCATCATCTCTACCTAGGGCTTGTGTATTATACTCAGACAATTCAGTGCAAAATCCACTTGAAGTTGCAAATGATCTTTCTAAGTGCTCCATCAAAGAAGATGAGAAACCACAAGCGGACCGGAGTTCAATGATCCCCATGACAAGGCTGCCTCTCCAAGTTCCTCAGAATGTTCAAGGTGGTGCTGCAAGACCTGGGAGGGTGGTCAGCTCAGTGTTGCGCTACAACAACTGTGGAGCAGCAGCTACAGCAGCAGAAGTCATTGAACAGCGAAGAATTGCACGGAACCCTGGCGGTCCAACTCAATATCCCATTTCCAATACTTCATACCCTAGAAGACATCCCAGCTGTAAAAATGAAAGGGGTGAAGATAGTACCTGCCCGGG

>A-E5

AGGTACGAATACGAACCGTGAAAGCGTGGCCTAACGATCCTTTTCAGACCTTCGGAAtTCGAAGCTAGAGGTGTCAGAAAAGTTACCACAGGGATAACTGGCTTGTGGCAGCCAAGCGTTCATAGCGACGTTGCTTTTTGATCCTTCGATGTCGGCTCTTCCTATCATTGTGAAGCAGAATTCACCAAGTGTTGGATTGTTCACCCACCAATAGGGAACGTGAGCTGGGTTTAGACCGTCGTGAGACAGGTTAGTTTTACCCTACTGATGACAGTGTCGCAATAGTAATTCAACCTAGTACCT

>A-F11

GGCCGAGGTGGGTAAAACATCTCGAGAGAGAGAGAGAGAGAAGAAAAAAGGAACGAAGGAAGATGAGTTTGAGGTGGTTAGAAGCTGTTCTTCCACTAGGAATCATAGCTGGTTTTCTTTGCGTAATGGGTAATGCTCAGTATTTCATCCACAAAGCTGCTCATGGCAGGCCGAAGCACATTGGGAATGACATGTGGGATGTAGCAATGGAAAGGAGGGACAAGAAAATGATGGAGATGCTTGCTTCTCCTTCTTCTTCTTCGTCTTAGTCGTTGTGTGGAAACCTGCGTTTGGGAATAAAATGGTCTTTTGAGGCTCGAGATCATGTGCAAAATACTTCAATATCGTCTTTCTTCCTTCTCCTGTTGCTGGTTTTCCATAGCCTATGAGCTTTATAACAATGAGACATTTGTGAAATGCTTCTTTGTTGAATCTGGTGCAGTTGTTGGTTTCTGTTTAAACTTTTGTGGAATTTTCACTAGTATTCATCGACTCTCTACTTGTAAACAGACTGGAATTTCTCTGTACCTGCCC

>A-F6

GACTCCGTCACCGGCGACATGGGTATCTCTCGTGATTCTATGCACAAGAGACGTGCCACTGGAGGAAAGCAGAAGACTTGGAGGAAGAAGAGAAAGTATGAGCTTGGCAGACAGCCCGCAAACACAAAGCTGGTGCCAAATGCTAAGACTGTTAGAAGGATAAGAGTCCGAGGAGGTAATGTGAAGTGGCGTGCTTTGAGGTTGGATACTGGGAATTTCTCTTGGGGTAGCGAGGCTGTTACAAGGAAGACTCGTCTGTTGGATGTGGTGTATAACGCCTCTAACAATGAGTTGGTTAGGACACAAACCCTGGTGAAGAGTGCAATTATTCAGGTTGACGCAGCTCCATTTAAGCAGTGGTATCTCCAGCACTATGGAGTTGAAATCGGTCGCAAGAAGAAGACTGCTGCTTCTTCCAAAAAGGAAGGAGAGGAGGCTGAGGCTGTAGCAGAGGAGAAAAAGAGTAACCATGTCCAAAGAAAGCTGGAAAAGAGGCAACAGGATCGCAAGATTGACCCACATGTCGAGGAGCAATTTGCTAGTGGGCGTCTATTGGCTGCAATCTCATCGCGACCTGGCCAGTGTGGTCGTGCTGATGGTTACATATTGGAAGGAAAGGAACTTGAATTTTACATGAAGAAACTTCAGAAGAAGAAAGGGAAGGGTGCCGGTGCTGCTGCTTAGAGTTGCAGGCTCCAATTTTTAAATTATCAGAACAAATTAGATTTTGGTTTTTCTTCCCTATTCAACAGTCTCACTTCATTTAAAGGGAATGATAAACTTCTTGTATCAGAGTTAGATTCAAGTTTGTTTTGCATATGCTTAACTGAGAATTTTGAGAACCACCTACTTTATGAAAAAAAAGAATTTTGACAAAAAAAAAAAAAAAAAAA

>A-G3

TCAACCTCTGTAAACCGGAGTAGTCATCTTCAATCAGCAATCATGGGGAAGACACGTGGAATGGGAGCTGGACGCAAGCTGAAGTCCCACCGCAGAAGACAAAGGTGGGCTGACAAGTCCTACAAGAAGTCCCATCTTGGTAACGAATGGAAGAAGCCATTTGCTGGATCTTCCCATGCTAAAGGCATTGTGCTCGAGAAGATAGGTATTGAGGCTAAACAGCCAAATTCTGCTATTCGTAAATGTGCTAGGGTTCAACTCATCAAGAATGGGAAAAAGATTGCTGCTTTTGTCCCTAATGATGGTTGCTTGAACTACATCGAAGAAAACGATGAAGTGCTGATTGCTGGATTTGGTCGTAAAGGTCACGCCGTGGGAGATATTCCCGGAGTTCGTTTCAAGGTGGTGAAGGTTTCTGGTGTCTCTCTTCTAGCTCTCTTCAAGGAGAAGAAGGAGAAGCCAAGATCTTAATTTATCTCCTCTCTTTCTTTTTTTTTGCAAAAATACCCTCGAGCCTGCAATGGACTTTTAGCTTGTATTCAAGTTATTATTTTGTCAACTTCTATT

>A-H7

TGAGTGGTGTTACTTGCTGTTTGAGATTCCCTGGTCAGCTGAACTTCCAGACCTGAGGAAATTGGCTGCGAATTTAATTTCCCTTCCCACGTCTTCACTTTCTTCATGGTGGGATTTGCCCCACTAACCTCTCGTGGATCACAGCAATACATATCGCTAACAGTGCCAGAGCTTACTCAACAAATGTGGGATGCCAAGAACATGATGTGCGCGGCAGATCCCCGTCATGGACGTTACCTGACAGCTTCTGCCATGTTTAGGGGTAAGATGAGCACAAAGGAGGTAGATGAACAGATGATCAATGTGCAGAACAAGAACTCGTCCTACTTTGTTGAATGGATCCCTAACAATGTCAAGTCTAGTGTGTGTGATATCCCACCAACTGGGCTGAATATGGCATCCACGTTTGTTGGAAATTCAACCTCCATTCAGGAGATGTTTAGAAGGGTGAGTGAGCAGTTTACTGCCATGTTCAGGCGCAAGGCTTTCTTGCATTGGTACC

>B-A2

AACATTATCCGGCACATCAAGATGAGTCACTTTTGCCAACCGAAGAAATTCTTCCACTATTGATGGCAAGCATACCATTAATGGGCTCAAAGGATGCCTCAGGATATCCTCTATATGCTTTGATATACGTGATCTGATGCGAGGAATAGCAAGCATTGATCTCATGCGAAAGCAAAGTACCTCGGCCGCGACCACGC

>B-A4

ATGAGAAGCAATCAATGATCAAAGAGCTAAACTTCCATATTGTTGGCCCTTTGAGAAAAACGTATATGAGCAGTCTTGGCAATAAGAAGGGTTGAACTATTTTGATATGGGAGCAGGTGGAAGTGAACCAACAGCAGAGGAAAACGTTCAGATGGCATCAGAAATGTCAAATCAGGAGCTCATCAGTGCTGGAAACAAGACAATGGATGAAACTGATCAAGCCATTGAACGCTCCAAACAGGTTGTTCACCAAACAATTGAAGTGGGAACACAAACTGCTGCTACCTCGAAAGGCCAAACTGATCAGATGGGTCGTGTTGTCAATGAGCTCGACACAATTCACTTCTCCATCAAAAAAGCATCCCAGCTTGTCAAGGAAATTGGACGGCAGGTTGCCACAGATAAATGTATCATGCTTTTCCTCTTCCTCATTGTCTGTGGTGTAGTTGCCATAATTGTTGTGAAGATTGTGAATCCTCACAACAAGGACATAAGGGATATCCCCGGACTGGCACCTCCAGCACCTGCAAGGAGATTGCTATATCTAAGGCCCGGACAAGATTTCATGTAAATCTTCAAGGACGGATCCATCCAATTTTCGATATGGTACCTGCCC

>B-A6

CCTCTTTGGAGAGAAGGCTGTTACAGTTTTTGGAATCAGGAACCCTGAAGATATCCCATGGGGTGAAGCTGGTGCTGACTTCGTTGTTGAATCAACCGGTGTCTTCACTGACAAGGACAAGGCTGCTGCTCACTTGAAGGGTGGTGCCGAGAAGGTTGTGATCTCTGCTCCTAGCAAAGATGCTCCCATGTTTGTTGTGGGTGTCAACGAGAATGAATACAAGCCAGAGCTGGACACTGTCTCCAATGCTAGTTGCACAACGAACTGCCTTGCACCTTTGGCTAAGGTTATCAATGATAGGTTTGGCATTGTTGAGGGTCTCATGACCACTGTCCACGCCATGACTGCCACCCAGAAAACTGTTGATGGTCCATCCATGAAGGACTGGAGAGGCGGAAGAGCTGCTTCATTCAACATCATCCCTAGCAGCACTGGTGCAGCCAAGGCTGTTGGAAAAGTGCTCCCACAACTTAACGGCAAATTGACTGGAATGGCCTTCAGAGTACCTCG

>B-A8

GAGGTACATATTGCCTTGATATCAGCACCCGAAAATTCATCCTTGGGTCATAACAAATTCTTCTAAATTGACACCATCAGCCAAAGTCATCCGTGCTGTGTGTATCTGGAAAACGCGCCTCCTTGTTTTGATATCAGGAAGAGGGAACTCAATCTTCCCGTCTATCCTACCAGGCCGAAGCAGGGCCGGGTCCAGACTCTCAATTTTATTCGTTGCAAGAATCACCTTAACATCTCCTCTGGAATCAAAACCATCTAACTGGTTCAGCAGGCTCCAACATAGTCCTCTGAATTTCTCGTTCACCACCTGAGTGTGCATCATACCTTTTTGTACCTGCCC

>B-B12

AGGTACTGGAAATCAGAATCAAACGAGCTTTTACCCTTCTGTTCCACACGAGATTTCTGTTCTCGTTGAGCTCATCTTAGGACACCTGCGTTATCTTTTAACAGATGTGCCGCCCCAGCCAAACTCCCCACCTGACAATGTCTTCCGCCCGGATCGGCCCGCGAAGCGAGCCTTGGGTCCAAAAAGAGGGGCAGTGCCCCGCTTCCGATTCACGGAATAAGTAAAATAACGTTAAAAGTAGTGGTATTTCACTTTCGCCTTTCGGCTCCCACTTATACTACACCTCTCAAGTCATTTCACAAAGTCGGACTAGAGTCAAGCTCAACAGGGTCTTCTTTCCCCGCTGATTCTGCCAAGCCCGTTCCCTTGGCTGTGGTTTCGCTGGATAGTAGACAGGGACAGTGGGAATCTCGTTAATCCATTCATGCGCGTCACTAATTAGATGACGACCTGCCCG

>B-B2

GGGCCGAGGTACTAACAATTATCCTTCAGAATCCAAAAAAAACAAAAGTTGAAGGGAAANTGGAAAAACACTTTTAAAAACCAAAACAACACAGAAACACAATTCATAACATTCCCAAAAGTAAAATTAAAAAAACATTTCATTTCATTTAGTCTTTTTCCTTCTCCTCTTCCTCTTCAGCCTTTAGGGTGGTATCCTAGGTAATTTCTCCTTAATTTTGTCCAAAAATCCCTTCTTCTCCTTCCCCTCCGCCTCATGCTCCACCGCAGCCGGCGGTGGTGGTGGTGGCGCCGCCACTTCCTCCGTCTTCTTATGCCCGCCACCTGGCAATTTCTCCTTAATTTTGTCTAGAAATCCTTTTTTCTCCTCTGTTTCCTCGTATTTCTCAACTGGAACTGAGGTATCCTCTGCTTTCGATTCTTCCTTGTGATCACCAGATATTTTTTCCTTAATCTTATCCTTCAATCCCCTCTTTTTCTTCTTCTTGATCTTCTGTCCATCCTCTCCAATTTCTTCCTCCTCATCACTGGAGCTACTAGAGCTGCTATTTGATCGATGAAGTTTCTTTTCCTCCTCCTTGGGTTCCACTTCTTCACTTACGTTAACTTTTTCAGACAACTCAGATGAAATTGCCTCTTCTTCATGAGCATGACTTGGTTTTTCCTCTTCTTTTTTTCCAATGAAATCAAACAAACCACGATCAGTAGTCTTCCACGTTGGTACCTGCCCG

>B-B3

TAGCGTGGGTTCCGGCCGAGGTGGAATATTCTATTCTTGTAGCAGCCACCGCTTCGGATCCTGCTCCTCTACAATTTTTGGCCCCATATTCTGGGTGTGCCATGGGGGAATATTTCCGCGATAATGGAACGCACCCATTAATAATCTATGATGATCTTAGTAAACAGGCGGTAGCATATCGACAAATGTCATTATTGTTACGCCGACCACCAGGTCGTGAGGCTTTCCCAGGGGATGTTTTCTATTTACATTCCCGTCTCTTAGAAAGAGCGGCTAAACGATCGGACCAGACAGGCGCAGGTAGCTTGACCGCCTTACCCGTCATTGAAACACAAGCTGGAGACATATCGGCCTATATTCCCACCAATGTGATCTCCATTACTGATGGACAAATCTGTTTGGAAACAGAGCTCTTTTATCGCGGAATTAGACCTGCTATTAACGCCGGCTTATCTGTCAGTCGCGTCGGGTCTGCCGCTCAGTTGAAAACTATGAAACAAGTCTGCGGTAGTTTAAAACTGGAATTGGCACAATATCGCGAAGTGGCCGCCTTTGCTCAATTTGGCTCAGACCTTGATGCTGCGACTCAGGCATTACTCAATAGAGGTGCAAG

>B-B7

GCCGAGGTACCCTTATTGGCAGCAGCCTTGTCTTTCACCTCCTTGAGGAAGGAGAGACCATAGAGTGTCGATGTTGACAGTAGGGCAATAGAATTTGTTACGAAGCTTGTGGAAATAACGCATACCTACTTTACCGAAGTAACCAGGATGGTACTTGTCGAAAAGGATCCTGTGATGGTGCATACCTCCGGCGTTACCTTCTTCCTCCTGGATGCTTCCTGTGCTTTCCGGATACGGACCATGTCCGGCGCTGGAACGTGGACCACGCTTCTTCCTGTTCTTCTTGAATCTGGTAGTCATTTTACCTGCCC

>B-C1

GCCGAGGTACATCATTTTAACTCTCTTGTTTCTATGACATGAAAAAAAAAGAAGCAACAACACATCAACTATGACACACATTCCAATGAACTAATAACATCACAGTGTTCCGTCATGCATAAAACTTAAACTCAATCAATAAAAAGACCTATGGAAGGTGGGGATTGCGAAGTCCTACCAATCTCACATCATCGACCACAGGACCACACAAGGAACCCGAATTATCACTCTTCATATGATAATACGTGCTCAAAAACCTCACTCTTGTGCGTGGTGAAATTGCGGTGAATCTAAGCCTAGCCCTTTTGAATCCACCTTTACCTTTAGATTCATAAGGAAACTGCATAGTAATTTTTCCAGCAAATGCCTCCACAAGCATTGATCCTTCACAAGAGTTGCTAGCACCTGCCCGG

>B-C10

TCATCAGTTAGGGTTAAAACTTAACCTGGTCCTCACGACGGTCTAATCCCAGCTCACGTTCCCTATTGGTGGGTGAACAATCCAACACTTGGTGAATTCTGCTTCACAATGATAGGAAGAGCCGACATCGAAGGATCGAAAAGCAACGTCGCTATGAACGCTTGGCTGCCACAAGCCAGTTATCCCTGTGGTAACTTTTCTGACACCTCTAGCTTCGACCTCGGCC

>B-C2

GCCGAGGTACAGAAACTTAGATCACATTACTTCATCTTTTACAGTAATGCAGCATATGCTAACCACTTGTTCTAGGAATAGGTAAAAAATCTACATCAGAAATTATACCTCTCAAATCATAACAGTTATCTTTCAAGCATTTGATGAAGAAAAGGGGAATTTTACTGACAAATCTACACAGGCTGAAACTAGGTTCTGGAAGAGCTACTATACACATTCCCCATTTCCCATTGCGACGTCCTTAATGCACTTTCTTGTTCTTCTCTGTGTTACTGAGTATACCCAATGAGTTCAAAAACTAAAGATCCAAACTTGGTGGACTCCGTTTCTCAAATTAAGAGTGGTCGCACTTGATAAACATGTCCCTTTATTTCTTCATTGGAAATGAGCTATCTTGGTAATACAGGACGAATCAAACTCCCTCCACCCTTAACAGAGGTCTCGGGTTCAAGCCTAGGAATGGAACAATTCCCATCAAATGATTCATACACAAATCTAGATAAATTAGCCCAATGGGTTACCTGCCC

>B-C7

GGCCGAGGTCGCGAAAAAGGCGACAAGACCTCGATCTACAACAAGAACAACAACAAAATCAGCAGCAGCAAAACGAGCAACAAGATGGGAAGTTATCCTGCTACAATCATCAAGTAGTGGGACTACGCCAACTAGTCATCCTTCAACTTCCAGCTAACCCCTGGATGTCGACGGAATAATAATACCCAAGTATCGGGAGGCGGATCCCAGTGTGGACGTTTCCANTCGGTTAACAAACTTCTGGTGCGGCAGGCTGCTGCTTTGTATAGAAGTACCTGCCCGG

>B-D1

CTCCAGTCTCCAGACTCCACGGAAGCTGATCCTTTCCGATACTATAACTGAAATTTCCTTGATCGAAACAGACAACTTTCCCCCATGGAAAGCATTTTTCCTTTGTCAGGTTCATCTATAGCAAAGCCATCACTCGAAGGCATTTGGAGCGAGTTCCAAAGAATCCAAGAAAGGGAAAGTGGTAACAATTTGGTCGAGATCATCTGTGAAGTTCTCAAGAAAGACCTGGCTCAAGACACTGTCAATGGTTGGGGAGACTGTCCGGTTGAGATGTCCGAGGCTGTTTTGGACATGGAAAGGCAGATATTCAAGGATTTGATTGTCGAAACGATTCAAGACCTTGCTGTGATTGGCTTCAAAACTACCCTCTTAACCGCTTCGCGTAGGAAGTTAGTTTTCTAAAGCCTCATTAATCCATTTTCATGTCCAAGCTTCCTTGTTTTCGTGTTCTTTTTAATTTGTTTTACCACTTTTTAGGTTTTGGTTACTATTGATTCTTGACTACCTAGGAAGGGTACTTATGTGTTGTAATAGTAGTCACTTCCCAAGATTGTAAAAAAAGGGAAAAGTGTTGTTAGACTGAACTCTCAAGTTTGAAAATCTCTTTTTCTTTTCTGTGAATGCAAAAAAGTTTTGAGTTAGTTGGCAAAAAAAAAAAAA

>B-D10

CCGAGGTCTTCACTCACTCCCAATCAAGGAGTTTCAAATCATTGATACTCTTATCGGAACATCTCTCAAGGATGAGGTGATGAAAATCATGCCAGTTCAGAAGCAGACCCGTGCCGGACAGAGAACCAGGTTCAAGGCGTTTGTTTGTTGTAGGTGACGGTAATGGCCACGTTGGTCTGGGTGTTAAGTGCTCAAAGGAAGTAGCTACTGCTATTCGTGGAGGAATTATATTGGCTAAGCTATCAGTGATTCCAGTGAGGAGAGGTTACTGGGGTAACAAGATTGGGAAGCCACACACTGTGCCTTGCAAGGTTACAGGGAAATGTGGGTCTGTTACTGTGAGGATGGTGCCTGCTCCTCGTGGTGCTGGTATTGTTGCTGCTCGTGTCCCTAAGAAGGTTCTTCAGTTTGCTGGTATTGAAGATGTCTTCACATCTTCTCGTGGATCCACCAAAACCCTTGGAAACTTCGTTAAGGCGACATTTGATTGTTTAATGAAGACCTACGGGTTCCTGACCCCAGATTTCTGGAAAGAGACTCGCTTCACCAAATCTCCATTCCAAGAGTACCTGCCCGG

>B-D5

GGCCGAGGTGGCTTGTGGTCTCGCTGGCCTTTCCGCTGGAATGGCTATTGGAATTGTTGGTGATGCTGGTGTTAGAGCAAATGCACAACAACCAAAACTCTTTGTTGGTATGATCTTGATTCTTATTTTTGCTGAGGCGTTGGCTTTGTATGGACTGATTGTCGGCATCATCCTTTCTTCCCGTGCTGGCCAATCTAGAGCAGAATAGATGAAAATAATGTATTTTCAGGTTTATGATGGAATCTTTTTCATCATTGTGCTTGCAGCTTGATAACCGATGCTAGTCGTTAGGTGCATCTTCTCTTTTCCTTTTTCTTCTCCTTTTCCTTTTCCTTCACACATTGTGTGTTCCCCCGGTGTTTGTAGATTTGGAAGCTGCCTTTTCCCTGTATGTGTGACCAAGAGAAATGTTCTCTCGATTTTACCTTTACTGTATTTTCCAATAATGAGTCGCATCTTGTCGCTTGAATGCTGATACGATTGTATTATTATTGGTGGAATGTAATGGTACCTGCCCGGG

>B-D7

CTGATTAAGAAGGCTGTTGCCATTAGGAAGCATTTGGAGAGGAACAGGAAGGATAAGGATTCTAAGTTCCGTTTGATTTTGGTGGAGAGCAGGATTCATCGCCTTGCTCGTTATTACAAGAAAACAAAAAAGCTCCCACCTGTCTGGAAATACGAATCTACCACTGCTAGCACACTTGTGGCATAGGCTGAGACGTGAGCTGGAGTAGCTTTGGCTGATCGCAATATGTAGTTTTCTTGTGTCATGAACTGTTTGCTATATCCAATTTTGTTTGATTTAATCATGCTGCTCAATGGAAAATAGTTTTCTGGATAGTATTTGCTCCTATTTTTACCAAAGTGTTAAGCATAGATGCTTTTATTTAGATATTCAAAATGAATGACTTGTTTCTCAAAGCTCATGGTGGTAATCTGTAATTTGGATTGCTGAAAATTGTGGTTAAATGCCTTCATCATTCTATGTTCATGGCAGTGAAGTACCT

>B-D9

GGCCGAGGTACAAATGATCAGTCAAATCCGACAGATATGAGTAAGCCGTTGGCAGATCAATCATTTGTGTCATCAATCCTTGCCTCACTTCCAGGTGTTGATCCAAACGATCCTTCTGTCAAAGATTTGCTTGCTTCCATGCAAGGGCAGTCCGAGAAGAAGGATGAGGACAATGATAAGGAACAGAAAGAGGACAAGAAGTAAAGATGCGTACCTGCCCGG

>B-E11

ATAATCAAACATTAACTGAACTTCATTTGTTCAATCATAGCAAAGTAACCAAATACTGAAATAAGACATAGCTATAGCCAATAGGCCAAGGTGCTCCTCAGTTCTCCACTCTTGAATGGAAAGGCTCTTATGATAACTTGGTGGTATAAGGCAGCAAGTGCAGCTCCAATGAATGGTCCCACCCAGAAAATCCAATGATCATCCCATGCATGTTCTTTGTTGTAGACAATGGCAGCGCCTAGGCTTCTAGCAGGGTTAATGCCGGTGCCTGTAATAGGGATTGTAGCCAAATGAACCAAGAACACTGCAAATCCAATTGGGAGAGGAGCCAGAATAGGGACATGGGAGTCTCTAGCATTTCTCTTGGCATCAGTAGCAGAGAAGACAGTGTAGACAAGAACAAAAGTGCCAATAATCTCAGCACCAAGGCCATCTCCCTTGGTGTAACCATGGGCAACAACATTGGCACCTCCACCCTTAGTCTCAAACAAAGATGGCTGGAACCCTTTGACAACACCAGCACCACAGATTGCACCAAGGCACTGCATCACAATGTAGAACACTGCCCTGGTTAAGGACAATTTCCTTGCCAGAAATAAACCAAATGTCACAGCAGGGTTAATGTGTCCACCTGATATGCCAGCAGTGCAGTAAACAAGGGCAAAAATCATACCCCCAAAAGCCCAAGCAATGCCTTGAA

>B-E2

CGAGGTACAGTCATGCCATAAGCCGTGAGGATCAGCTGTATGAGGAGCTAAGCTTTCCTGAAGTTGCCAGGAATCATAGGCAGCATTCAGTAGAAAAAGAGGAGTCCTGATGTTGTTGATCAAATTCTGAGGAAAGAAACACGAGGTTGCATCAAGGTGGTTGGTGCAAGTTCTTGGTAGCGTATTCTGAAGACCCTGTGTCTTAACCACACCCCCAAAGAAATCTCTGATGGCATGTCCTCCAGATACATCATTTGCGTCCATAAATAATCCAGCATCACTCAGGCACTTCACTTTAGTATGTGGGAGCGAATTGCTGAAATCATCACAATGCATTATTGAAGCTAGACCACCAGCAGAACATCCAGAGAGAAGAGCCTGCTTGGCGTATCGCATTCCTTTTGACCTTAACTCCTCCATTGCAGCTTGATATATGCGCTTGCCTCTATATTGCAACCCTGCAGCCTTATCTTCACTATCCCCGGTAAATGAGGCACCATCACAGTAGCGAACTTTTACTCTATTCCAGTTATAAAAATCTGGATTTTCTTCAGCCCTGTTACTCAAAATCCCAACAAAAGGAATCTGTTTTTCCATGTAATTTGATGATCCTCGTCTTGTTTTTTT

>B-E4

GAGGTACTGGATTGGCACCATTCAGGGGTTTTCTCCAGGAAAGAATGGCTTTGAAGGAGGAAGGAGCTGAACTTGGTCCTGCAGTGTTATTTTTTGGATGCAGGAACCGCCAAATGGACTACATCTATCAGGATGAGTTAGATAACTTCCTTTGAGGCCGGTGCACTATCTAATCTAGTTGTTGCCTTCTCACGTGAAGGACCTAACAAAGAATATGTTCAACATAAAATGACACAGAAGGCGGACGACATCTGGAACATGATTTCTCAGGGAGGTTATGTTTATGTCTGTGGTGATGCTAAGGGCATGGCCAGGGATGTCCATCGGACCCTTCACACTATTGCTCAGGATCAGGGATCACTTGATAGCTCCAAGGCCGAGAGCTTTGTGAAGAAATTTGCAAACGACCGGAAGATATCTGCGTGATGTGTGGTAATTGTCTCATGGCAAGCCTATTGTGATTAGAGAAATATTTAGGAAGGCGAGGACTTGCAAAGAACAATCAGCCTTCCAATAGGGTGGGTAAAACAAGTGTACCTGCCCG

>B-E8

GGCCGAGGTGATATCTGGTGACATATAGCAGCCATGAACCAAGTTAACCCTTCGTGACACTTCACAGGGTTTGTGCTAAATATTTTTTGATGTGAGAACTGGAAAAGTGATGAGAGATTTCAAGGGAAGTGCTGATGAATTTGCAGTTGGAGGAACTGGAGGTGTTACTGGTGTGTCCTGGCCAGTCTTTAGGTGGAGTGGTGGTAAAGAGGATAAGTATTTTGCAAGAATAGGAAAGAATGTCATCTCTGTTTATGAAACAGAGACTTTCTCACTTATTGACAAGAAATCTATCAAGGTTGAAAATGTTATGGATTTTAGCTGGTCACCAACAGATCCAATTCTTTCACTCTTTGTTCCTGAATGTGGAAATCAACCTGCCAGGGTAAGTCTTGTGCAAATCCCAAGTAAAGAGGAGTTGAGGCAAAAGAATCTCTTCAGTGTGAGTGATTGCAAAATGTATTGGCAAAGCAATGGAGACTACCTTGCTGTCAAAGTTGACCGGTACCTGCCCG

>B-E9

GGCCGAGGTCTTTCTCTTTAGGGTTATTTGGGGTTTGCTATGGCGTCTTCAACAGCTCAAATTCATGCTCTTGGAGCTACATATTTCGCTAATTCATCTTCTTCCACTAGAAAACCTTTAAAGTCTGTGTTTTTGGGCCAGAAACTGAACAATAGAACCCTAGCTTTTGGATTGAGGCAGAAGAAGAGCCGGGGGAATAACGGTGGTTATGCACCGATGCGTGTGGTGGCGGAGAAAGGTGGTGGAAATTGACTTGGGGACTACTAATTCTGCTGTGGCTGCTATGGAAGGAGGAAAGCCTACCATAGTGACGAATGCTGAAGGACAGAGGACAACTCCTTCAGTATTCGTCACACCTGCCCGGGCGG

>B-F5

GGGCCGAGGTACCAACTTAGTTTCAAAGTTGGAGATGCAAGTAATGCTTGTGAAGGTTCTATGATTGTTGAGGCCTTTGCTGGAAGGGATACACTTAAAGTCCCATATGAGTCTATGGGAAAAGGTGGATATAAGCGTGCTATCCTCCGTTTCAAGGCCACTGCTAGCCGGACTAGGATCATGTTCCTTAGCACCTACTATCACACGAGGAGTGATGATTTTGTTTCCCTGTGTGGCCCTGTGGTCGATGATGTGACTCTCTTGAGTGTAAGGACCCATCGTCGAGTCCTCTAAGACATGTATTTTGATGAAATACGGGGGATTTTATGAGTTAAATTTGGTTGTATTAATGAAGCAAAGAAGGTGGGAGCTCTATTTTATCTTACTTTTGGTTTGTTTACTACTAGTGGCATTATCTTATGCATGTGTACCTGCCCGGGCGG

>B-F8

GTTTAGGGTGACAGTTACCTTCCTCCAACTACTGAAATATCTTCTTTTTTTCCAAGAATTCTCTGTTTTCTTGATTCTGTTTGTAGCCATGGCAGAATTGGAAGCTAAGAAAGTAGAAATTGTGGACCCTGCCCCTGCACAAGAACCAGTTGAAGCTCCTAAAGAAGTGGTGGCTGATGAGAAAGCCATAGTTGAACCAGCTCCGCCTCCTCCTGCAGAAGAAAAAGAAAAACCCGATGACTCGAAAGCACTAGTTGTTGTCGAAAATAAAGCAGAAGAAGCTGCTGATGAGAAAAAAGAGGGATCTATTGATAGAGATGCTGTGCTTGCACGCGTTGCAACTGAGAAGAGGCTATCACTCATCAAAGCATGGGAAGAAAGTGAGAAATCAAAAGCCGAAAACAAAGCTCAGAAGAAGGTGTCTGCAATTGGTGCATGGGAGAACAGCAAGAAAGCAAACCTAGAGTCTGAGCTCAAAAAGATGGAGGAACAGTTGGAGAAAAAGAAGGCAATATATACTGAGAAAATGAAAAACAAAATTGCTCTACTCCACAAGGAAGCAGAAGAAAAGAGAGCGATGATTGAAGCTAAACGTGGAGAAGATCTTCTCAAGGCAGAGGAGCTTGCAGCAAAATACCGCGCCACTGGAACTGCTCCAAAGAAAATCCTTGGAATATTTTGAAGCAGCAA

>B-G10

CCGAGGTACAATTATCTTAAGCCAACACAAAGGTCTGGTTCATTAAGATAACTGACCAACCTTAAGAGGGCTTCTTGCTCACATTCAATGCACCAACCATTGTGTTATTAGACTTCGTTGAAGAGACAAGATATGTGAAAATTTTCAAAAACTAAGCAGCATTGGATGCATTATTATGCAAAAATGGAGGAAGATGATGCTCCAAAACAGCCAATAGTTCTCTCCCTTGCTTTACTTCTTCAAGCACTACACCGTCGAGCCTTTTTGCTGCTTCCACAAGCTGGCCCTTAAGTCTTTGTGATAACAAACAACCCCCTGCCTTGACACATTCCCTATACAGTGGCAGGTATGCTATTGCTATCCTCAATGGCCCCGGGATGTCCCTCAAACACACGGGAGATTCCCCCCATTTCATAATACGCAACAGGTTACAGTAGTGTTCTTGACCTGCCCGG

>B-G3

GGCCGAGGTACGACGTGAGATGGTATCCAGCTCAGGTGAAAGATTGAACCACTGAGGAAGTTGGAAACCAAACCAGAAAGAAGGGCAACAACCACAGCACTATTGTATGTGGTT-GGCTCTCCTTGAACCGAGTTGTTCCTTGAATCGATATATGTCTCATTAAGAAATGGACCACCAACGAGAGCCCCAGTTGCAACGTTAGGATTTGGTTCAGTTGAGTCTAGATACTTCCAACCTTCTTTGCAATTAGTATTGGCGTCTGTAGGAATGGAAGCCCCTCTGTGGTGAACATACTGCGGATATTTGTCCCCATAGCCTACGAGATAACTCATCTTTGCTGGATTGTCACCCAATACATAATTGGCCTGGGACATGGCGAACTTTCTGAGATCAGATGGTGTATACTCATTACCATCACAAGTCATTTTATCAGTCTTGGAAGTGAGCATATAATCACTGTACCTGCCCGGG

>B-G5

CCGAGGTACATCATTGCACAGTTGCTTGGATCCACTGCTGCTTGCGCTCTCCTTGAATTCGCCACCGGCGGCATGAGCACGGGATCATTTGCATTGTCAGCCGGTGTATCAGTATGGAACGCGTTCGTATTCGAGATTGTGATGACTTTCGGTCTCGTTTACACTGTTTACGCAACCGCAGTTGACCCAAAGAAGGGAGATTTGGGTGTGATTGCACCAATTGCAATTGGTTTCATTGTTGGTGCCAACATTCTTGCTGGTGGTGCCTTTACTGGAGCTTCAATGAACCCAGCTGTGTCATTTGGCCCATCTTTGGTTAGCTGGACCTGGACTCACCAATGGGTTTACTGGGCTGGACCACTTATTGGTGGTGGGCTTGCTGGATTCATCTATGAATTCATCTTCATTAGCCACAACTCATGAGCAAATCCCAAGTGGAGATTTTTAAGTAGTGCTGAATTTTTATCAACTCGTTGTTTAATTTTCATTTTCTCTTTGTAAACTTCTTTGTTTGTTTGTCTATGTTTAATGGGCACTGTTGAATTGTACCTGCCC

>B-H10

GAGGTACATGTCAAAATTGCTAACCAATGAAATCCCTCCACGTCAAATTTGTTCGTAAGATATTTTAAGGTTATGAAGCCTGACTTGCAGAGTGAATGGTAAAGGAGGACATAAAAAGCTTTAGTCCAAATACCGGATAGAACATACTCATTGCTGAAGAACCATACGCAAACTTCCATGAGGAAGAGCCAATGGGTAATCAACTCAGTTAGACGGTCTCTGGCATGCAACAAACAATTGCCAACTGATAAAAGCAATCAGCAATGAGCTGAAAAGCTCAAAGCCAACTACTTTTGGAAGGTGCCACCCCAAGCCTCGCCTCAGATAACTGGTTATGGTTGGCATTGTTGCCACAATCCCAGCACATGCATATATGATAGGTATCAAAGTCTTGGGTTTGTTTTTCCGAAGCCACATAAGAGAACCCAATGCTGCTAGTGATATACTGAAAACCAAATTTGCATTTGCTTCTAGGCCTTGCAAGATGTAGTCCCATGTAAACTCCAATCCTCTAGCACCAAACCAAAGAGGTACCTGCCCGG

>B-H12

GGCCGAGGTACAAATCTCTACCCTTACACTCCACGAAAGAATATAAAAAAAAGGAGGCAATGGTGGCCAAATTCTCTAAGCAGATCCAAATGAGGACCCCTTCACCATAAATAACCAAAAGATAAATTATACATCGTCAAATATCAAAATATGTTTTGAACTCTTTAGAACAAGAGCCAGCTCATCACAGCCCATCCAGCTACCAAACTTCCCAGCATCTTCAATTTCTCTGCTCCACTCACATCGTTAAGAGATGGGCTGGGAGCTGAATCGGAGTCCATGCTAGGTCCAGGAGCTTCGGAAGGAGGTGCAGGTGGGCTCATCATATCGGGAGAAGGTGCTGGTGATGCATTGTGTTTCTTTCCCTTCTTTCCCTTTACTTTTCCTTTGCTAGGAGCCGGAGCTGGAGTTGTCTCCGAAGCAACAGGCGCAGCAACTGGAGCTGCAGCCGGTGGGAGTAGGAAACAGCTGGCGGTGTAGCTACCTCTGGGGCTGGAGCTGGTGGAGATTGTACCTGCCCGG

>B-H3

GCCGAGGTACAGCCATCGCCATTGGAATTGGAATGAATTGGGAAACTTTCGCTGGAGTGACATCCCTCAAGAAGGTTAATGGGCCAGTGCTGCCACAAAGAACCCACAACACAATGCAAGAACAATGCTTTGGAAGTTCGGAGAATCCCTCAATACCCAGAATGGCCATCTCTCGGTAAATAACAGCATATGGTGCTTTGTATGGACTATCAGGAGATCCAATGTCAAAAGCATTCCAATACATCCAGAATGTGAGGGGCGCAATAATGCAACCCATAGCTGTTCCCACCAACTGACTCACAAACATCGATTTAGCTGATGAAAGTGTAAGATACCCCGTTTTGAAGTCTTGCATTAAATCAGCTGCAGTGGAGACTATGGACATCATAACTCCACATGCTGCCAATCCTGCAACGACCCCACCATTGCTTCCAACCAGTGACGCAATAATAAAGGAGGACCAATCTTACCATAAGTTGAAGCCAAGCTCCAGTCTGTAAGTCCCGTACCTGCCCGG

>B-H8

GGCCGAGGTAGCGTCTCTCTGTGGACTATGGAAAGAAATCAAAACTTGGTTTCACCATTTATCCATCACCACAGGTCTCAACCTCTGTGGTGGAACCTTACAACAGTGTCCTGTCAACCCACTCCCTTCTTGAGCACACTGATGTTGCAATTCTTCTTGACAATGAGGCTATCTATGACATCTGCAGGCGCTCATTGGACATTGAGCGTCCCACATACACCAATCTTAACCGTCTTATTTCACAGGTTATCTCTTCACTTACTGCTTCTTTGAGGTTTGATGGAGCCCTGAATGTTGATGTCAATGAATTCCAGACCAACCTTGTTCCCTACCCCAGAATTCATTTTATGCTTTCATCCTATGCTCCTGTCATTTCAGCTGAGAAAGCCTACCATGAGCAGCTGTCAGTTGCAGAGATCACCAACAGTGCTTTTGAGCCATCTTCCATGATGGTCAAGTGTGATCCTCGCCACGGCAAAGTATATGGCTTGCTGCCTTATGTTCCGTGGTGATGTTGTGCCAAAGGATGTGAATGCTGCTGTGGCTACCATCAAGACTAAGCGCACCATCCAATTTGTTGACTGGTGCGCTTAGTCTTGATACCTGCCCGG

>C-A3

GGGCAGGTACACAGAACCTAAAAAATTTTAAGAGCTAGTAAATTTGGTAACAGCCTTAGTTCCTCCAGAAACAGCATGTTTAGCTAATTCACCAGGAAGAACAAGACGAACGGCAGTTTGAATTTCACGAGAAGTGATGGTAGGCTTTTTGTTGTAACGAGCAAGACGAGAGGATTCCTGTGCAAGTTTCTCGAAGATATCGTTGATGAAACTGTTCATTATACCCATAGCTTTGCTAGAAATACCGATATCTGGATGTACTTCCCCATTAGGAAATATGCTATCAAAGTGTAGGGATAGGATCTACTTTGGCTGAGATTTTTGCTTTGGATCAAAATTATTTGTTGAACTTATCAGCAATACAAGTTTTGTTTTGCTTTTATTCCCAACACATTGATCATGTACCTCGGC

>C-B10

TGTCTACCTATTGCAAGAAGACAAACAAAAATGCAGGAGCTAAACGCAGCAGATGTAAATCAAATCCAATTACCATCTAGAGAAATTCTCTGCCATACATGTTCCTCCCTAAAATTAAATATCGAGAAAATGAGGAAACCCAAAGGAGGCAGAGATGACTAAGAGAGTAAAAACTCGACTAGTATGTTGCCACTATTTACACGAATGGAGGGGGAAATGCCATACATTATCTTGCAAGATTTTCAAGCAGCAGGAGGTCTGAGCTCATGATCTTGAGATGTGTCCCGCGAAGATGGTTGTAGATACTGCCACATTGGCACCTGCCCG------------------------------------------------------------------------------------------------------------------------------------------------------------------------------------------------------------------------------------------------------------------------------------------------------------------------------------------------------

>C-B2

GCCGAGGTACCGGCCGGAGAAACGGGAGGTGAATCTCTATTCTGACTCCCGGAAGGCTTTACGATCATGATACTTCTCGTCACCTTCACCGCGTCCTCACTTGCTTCCTCAGTAAAGGATCTCGGTGTTGAAACTTCTGATTCCTTGCCGGAATTTGAACGCTGACTAAAAGTAGAATACTTCCGGAGCTTCCCGAGGCCACTATCTGGCCGGGGACCGGCAACGGTGTCGTCCCAGAGCTCGTCAAGTAAGCTCATGGTGCAGGTTAGCGAATCGGCTCACGTTAAGGGGAGAAGAAGAGAAAAGGGAAATTATACGTCCTGCAAGGATAGAACTTTCTCGGCTACCTGCCC

>C-C5

GTCGCGGCCGAGGTACAAATGTCATGTGAAATTCAGCTCAAAACAAAAAGAATCTCTTCCGCGGTGTAGATTACTCTTTGTTCTAAGCAGAACAGCACCCAGCTTTCTTTACTTCAGAAACATCATCCTTGCCTCCAACATTTATCGTCTGCCCCTTGGGAACTGCTGCCGGGACCTGCCCGGG

>C-C7

TACAGTTCTTTATCCCATTGATTTCAGCATTTCTTTGAGCATCAGAAACAGCAGAAGCATTCATTTCAACGCCAACAACCATACCAACTCGATTTGCCAGAGTAAGACCAATTGTTCCAGTTCCACAGCAAACGTCAAAAAGTAAGGTATCAGGACCCAAGCTGGCCCAGTCCCCTGCAAGAGAGTATAACTTCTCTGCAGCAAGGGTGTTCACCTGAAAGAAGGCCGTCGGCGAAATACAAAACTTAAGTCCATTAATAAAATCGTGAATTTTTGCTTCAGCAACTGCATTATCTGCATGCAGTTCAGAATAGCTTTCCTCTCTGGAAAATGGAAGTACCTGCCCGGGC

>C-C8

CCGAGGTTGTGGTTGGACATATTCTTCATTCTTTTATCCTTGTGCCCTATCATGGATGGAGAATTGGCCACAAAACTCCATCATCAAAATCATGGAAATGTTGAAGCCGATGAATCTTGGGTGCCGATGCCAGAAAAGCTATACAAGGAGTTGGATTATGCAACAAAGCTTTTAAGATTCAAGATACCTTTTCCCTTGATAGCATATCCATTGTATTTGATGAGGAGAAGTCCAGGAAAAAAAGGTTCTCATTTTAATCCATACAGTGATTTGTTTCAAGAAAATGAGAGAAAATTTATTGTTACATCAACATTGTGTTGGACACTCATGGTTGCTCTCCTCTTCTATCTTTCTACCGTCGTTGGCTTTCTCCAACTGCTTAAGCTTTACGGAATTCCCTATATGATTTTTGTGATGTGGTTGGATTCTGTCACATATTTGCATCACCATGGTCATGAAGAAAAGCTGCCTTGGTACCTGCCCG

>C-C9

CTGCCCTTGTTACTGGTGGTTCCTAAAGGCATAGGGTATGGGATAGTAGAGGAACTAGCAAGTCATGGTGCATCAGTTTATACATGTTAACGTAATCAAAAGGAGCTTAATGAGTGTTTAATTCAATGGAGAAACAAAGGTTTTAAAGTTGAAGCTTCTGTTTGTGACTTATCATCAAGATCTGAAAGAGAGGAGTTCATCAAGACTGTTGCTAATCATTTTGATGGAAAACTCAATATTTTGGTTAATAATGCTGGTATTGTCATATACAAAGAAGCTAAAGATTACACTATGGAGGATTACTCTCTAATTATGAGTATCAACTTTGAAGCTGCTTACCACTTATCTGTACCTCGGCC

>C-D5

GGCCGAGGTACGTCAAGATTTTATGTGAACAATAAATGATAAAACCTCTTGGACAAAATTCCTTCAAGACTGGCTTAGTTCAACCAGAAGAATGGCATGACACTGTATGTGGTTTTTATTTAAAACGGGCAAAATATGCCTTCCAAATATAAAATGAGAAATGGACGACTCCTGACCACTAGCAAAAGTATTTTAATATTCCAAAGAAATCTTCACGCAGTTGCTTCCTTGAGATAAGCAATGAGATCCGCCCGCTCCTGTGGCTTCTTCAACCCAGGGAAAACCATTTTTGTCCCAGGAATATACTTCTTCGGGTTGAGCAAGTAGTCATATAATGTGTTCTCTCCCCAGTTCACAGCCATATTTTTGTTTGCATTGGAGTACCTGCCCG

>C-E10

GGGCAGGTACCCAGGTTATTCACCAGATCAGCCTTTGTCATACCAATACCACTATCAATGATAGTGAGAGTATTATTGGCCTTGTCTGGAATAATATGGATGAAGAGCTCTGGTTGACCATCTAGCTTGCTCTTGTCGGTTAAACTCTCAAAGCGGATCTTGTCTAGAGCATCGGAAGAATTGCTGATGAGTTCACGGAGAAAGATCTCCTTGTTGCTGTAGAAAGTGTTGATGATAAGACTGAGAAGCTGATTGATCTCAGCCTGGAAAGCAAACGTCTCTACGTCCGACATTTTTTTGTAGGATCTAACGAGCTGAAGAAAACTGTAAGGCGAAATGGAACACAAAGGGACACCTCGGC

>C-E8

ATTATGGAGACGATTTAACAAGGGATAATGATCAAGAGGATGAAGGCATTGCCCCCTTCTCTGGGAAACTGAACAAGTCAAAGCAGGGACAAGCCCGTGCTAGTGTGTTTTCTACAGTTTTTGATACAATTGGTGATGAAGAGTCCGAGGAAGATAAAGAGCCTGTCGTTGCTGGTGGTATGGGGAAGACTATTGGCAATTCATTTAGTGTGGCGCTTCTTGATGAAGAAGAGGAGGCAGATACTTCTGTTTCTAAGTTTGAGACAGAGACAGTTGAAGAGGATGATGCGCCAGAACTTATTTTTGTAGATAAAAAGAAGTCATCCAAAAAAAAAAAAAAAAAAAAAAAAAAAA

>C-F4

GGCCGAGGTACATTGCAATGCAAAAACAACTTGACTCTTCAAAAGACACAACACCAAGCAGCCATTCTAAAAAAAAGATAGGCTTTGGTGGCTCAACTTCATAACATCATTAACAAGCAAATAAACCTAGTATAGATAGAGTATACGGTCCCTCGTTAAGCTATAAGTTGCTGAGCATTACTAAAATGTTTCAAAGATCACAAAAACTTGATGGCACGGCCACAAGCTCTACACGACCGGTCCATTCCTCTCCTGGCTTCAAGGTGATGGGTTTTTCTATTGCTGCTCCATCAACACAAAGCATCTGTTTATACTCATCATCACCAAAATCCACCATTGCTTTGGCTTTCTTCTCCCATGGATTCCACACCACAGTATCTACCTGCC

>C-G1

GGCCGAGGTACTTTCAACCAAACTTGAAACCACCAGATGGAAGTTGAATATCATTTCCACCAAAATTGAACTCATTTTGTGCCCCATCACCAGCCGGCACTGTTTCATCCTCCTCCTCCAACCAGTATGTCTCCAGTATTTTAACAGCCTTCTCATATATTTCATTGTTATCATGACTCTGAAGGTTTTCAATCTTTTCCAATCCCTCAGCATCATCAATCAGCTGAGCATAGTAATTAATGCCTTCAGTGTTGGCTTTTTCTGCTTCCCCAACCTTCAATATGTTTTCTAATCCTTCTAGACAGACCGTGACAATCCTTGGGTCAGGGCACACAAGCAAATCACACAGAGGCTTTATGCAGTTTTGACTCACCAAAAACTTGATCTGCTCACGACTCCCACCGGAAGTAGCATTTGAAATAGCCCAGGCAGCCTCTTTTTTGATGTCAAACTCTGCAGTTTGAAGCAAATTGACCAGAGGACTAATTAATCCAGCCTCAATCACAGCCTGTATCTGTTCCTTGTTCCCAGCAGTAATATTTGATATAGTCCAACAGGCCTCTTTCTTGATGCTCTTTTTGTGACTGTGAGATAACAAGCTCAGCAGGCAAGCAAAGGGCGCCATGTTCAATAATGCACTGAGTCTGGAGGTCATCTCCTGTAACAATATTTCCTACTGTACCTGCCC

>C-G5

GGCCGAGGTACCGTATTAATAAGATCTCTAGGTGAAGGAAGGATAGCTCCAGCATGATAGTAATCAATCCCTAAACCACCTCCAATATTCAGGTAATCAATTTCAAATCCTTGAGATCGGATTTCGTCAATGTAGTTCACCATCAAGGCAGCTGCATCTCGAAAAATGTCTACCTTGGTGATAGTTGACCCAAGATGACAATGAGCTCCAACAAGTTTCAATTCCTGAGGATGTGCCTTGACAGCATCTAGAAACCATTGCAGCTTCTCATTTCTGATGCCAAATTTGGAGCTCTTATTCCCAGTGGCAACATAAGGATGAACCTGGGGATCTACATCTGGATTAATCCTGAGTAGCACGTTAACTTTCTTCCCAGCCAGTCTTGCAGCTGCTACAATGTTATCCAAGTCAAATTCACTGTCGATGTTTACAAACACACCTGCCC

>C-G7

GTGGTCGCGGCCGAGGTACCTCATCAGCCTAATTAATTAGTTTTACTCCCTATTATATTGCACTTGGTTGAGTGACATCTATCTTTATATGAGCAAGAATCAGTTTAATTTTTGTTGGATATTGCAAGTTCTTATTAGTAGTCAAAGACCATTCTGCAGTTTGCACTATTCCGTATCTAATCAAAAAAGGGATTTTTTGCGTTGAAAAAAATCCCTTTTTT

>C-G9

GGCCGAGGTACACAATACACACATCAACATGTTCAATGTTCACGATAAGATAGAAGTTTAATCCGACACCAAGCTTAGTAAAAAACAGAAGCGACAAATTTAACATAAAAGCCTTTAGCTTTTTGCCAGTTAAGGAGTTCCGCACATTGTAAAATTAGTCTTCTGCATTCTTCACAACAGTAACAGGGCACGTAGCATTATTCACCACATAGTTGCTTACACTGCCCATAATAACCCTCTTGATCTTTCCAAGCCCTCTGTTCCCTATGACCAGACAGCTCAAGGGAGTTTTATCAATTGCTTCACATAACTTCTCTCGAGCATCTCCCCAGAAAATTTTCATGACTACTGTGATCTCCATTCTGCCTAGCTGCAAAGGGTTATCAATTTGCAAATGTTTCAGGGTCGGGGTTAACCCCATACCTCTTCATTGTGTGGCCATCACATATCTCACCTGCCC

>C-H4

GCCGAGGTTGATAAAATGCGTGGACGTGACCACACAGTATCTGCAACTCATGGAGACATGGATCAGAACACAAGAGATATCATCATGAGGGAGTTCCGATCTGGCTCATCTCGTGTGCTTATCACAACCGATCTCCTTGCTCGTGGTATTGATGTCCAACAAGTCTCCCTTGTTATTAACTATGACCTGCCAACTCAACCAGAAAACTACCTGCATCGTATTGGTCGTAGTGGACGATTTGGAAGGAAGGGTGTTGCTATCAACTTTGTCACCAAAGGATGATGAAAGGATGCTTTTTGACATACAGAAGTTTTACAACGTCGTAATTGAGGAGCTCCCAGCCANATGTTGCTGATCTCCTCTAAGGCGATTTTTGGTATATTTATTCTTAGATTTAGTATCAGTTTGTACCTGCCC

>C-H6

TTTTTTTTTTTTCCGCAAAAAGTATCACCATTACCTAAAAACTACAATGCAGGAATAATAAAATGGAGTTTTCGGGGATAAAACTTAAAATGGAGTTTTTGGGGATAAAACTAACTGACGAAACCAAAAACATGAGGATTGAGGAGCGAAAACAGCTTAGTCGACCTCCTCGATCTTAGGACCAGCACCGCTGCCACCGCTAGGAGCAGGCCCATCATCACCCATGTCAGCACCCATGTCACCGCCAGCACCTTGGTACATCTTGGCAATAATTGGATTGCAGATGCTCTCCAACTCCTTCATCTTGTCTTCAAATTCATCAGACTCAGCAAGCTGGTTGGCATCGAGCCACTGGATGGCCTGCTCAATGGCCTCCTCAATCTTCTTCCTGTCTGCCTCAGGCAGCTTGGATGCAATCTTCTCATCCTTGATGGTGTTCCTCATGTTGTATGCATAGTTCTCCAAAGCATTCTTTGCCTCTACCTTCTTCTTGTGGTCTTCGTCCTCAGACTTGTACTTCTCTGCTTCTTGAACCATCTTCTCAATCTCCTCCTTGGAGAGTCTACCCTTGTCGTTGGTGATGGTGATCTTGTTCTTTTGTCCAGTAGTCTTGTCCTCAGCAGAAACATTCAAGATACCATTGGCATCAATGTCAAAGCACACGGTGATCTGAGGGACTCCCCTGGGAGCAGGGGGAATACCAGAGAGCTCAAATTTGCCAAGCAAGTTGTTGTCCCTGGTCCTAGTTCTCTCACCTTCATAGACCTGGATCAACACACCAGGCTGATTGTCTGAGTAGGTTGAGAAGACCTGCTCTTTCTTTGTTGGGATAGTGGTGTTTCTGGGGATCAACACAGTCATGACACCTCCAGCAAGTTTCCAGTCCAAGGAGGAAAGGAGGGGTAAACATCCAACAACAACAGGTCTTGCACCTTCTCGTTACCTTCACCGCTCAAAATTGCAGCTTGTACCTGCC

>D-A1

GCGGCCGAGGTACCTTCTGATTCTCCAGTTTCATCTCCACCAGCTCCACCAACCGTTGAGACTCCGGCTTCTGCTCCTACCGCCGGTGGTGCTCTTGCTCCGTCCATCGGTGCTACACCAAGCGGTTCTCCAACTTCCTCTCCAAATGCTGCATCCTTGAACAGAGTCGCTGTTGCTGGATCTGCCGTCGTAGCTATCTTCGCTGCGTCTTTGCTGTTTTAGATCTGAGGAGATATTTGCATTTGGATTTGCTGGAGAGAGGGATGTTTATGATTTTAGGATTTTTATATTTTTAGTTCATCTTACTCGTTGATGTTTATTATTCGTTTTGCTACTTTTTCTTTTACCTTTGGGTGATGGTGACGATTACGTACCTGCCCGGGCG

>D-A10

CCGGGCAGGTACAAGACAACTGACTTTGTGCTAAAGGCACTTTTAGAAGTTTCAAAGTAGTGAAATATAAGATACCATCTTAATCTTATTTCAGAACCATCACAGCTTATTAGATGACAGTTGGTTGCCATATTTCAGATTAACTTCAGGAATTTCAGTGGGTAACCTCCACCAGACCACAGAGTCTGTAACCCCAGTTCTTCTCCGATGCATGATCACAAATGCCATCAGCAGCATGAAGACAGCAGCTAGCATAGGATATAGGACGTTACCATTTCCCCTCTTCCAACTCTTCAAGACGGCATGTGCACAAATCTTACCATCAAAACTACCCACGGAATTATTTATCTTCATGATCTCAACCCCATTCAGAATGGCATCTACAGCATGTGGAAGACTCGTATTCGATGGCCCAACACTCAAAGTCAAAACACCGGAACTATCTCCATCAACGACAAAATCAGCATAGAAAGGAGAAGCCAGCAACCTATTTGTTACTTCAGTAAGGTCCAAGTTTTCGTAGGCTAAATTGTTGTTCACATAAACATTGAAAAATAGCATACCACGCGCAATACTAGCTATATCACAGAAGTGCATCCTAACCAAGTACCTCGGCCG

>D-A2

CGAGGTACTCATTCCAATTACCAGACTCATAGAGCCCGGTATTGTTATTTATTGTCACTACCTCCCCCGTGTCAGGATTGGGTAATTTGCGCGCCTGCTGCCTTCCTTGGATGTGGTAGCCGTTTCTCAGGCTCCCTCTCCGGAATCGAACCCTAATTCTCCGTCACCCGTCACCACCATGGTAGGCCACTATCCTACCATCGAAAGTTGATAGGGCAGAAATTTGAATGATGCGTCGCCGGCACGATGGCCGTGCGATCCGTCGAGTTATCATGAATCATCGCAGCAACGGGCAGAGCCCGCGTCGACCTTTTATCTAATAAATGCATCCCTTCCAGAAAGTCGGGGTTTGTTGCACGTATTAGCTCTAGAATTACTACGGTTATCCGAGTAGTAGATACCATCAAACAAACTATAACTGATTTAATGAGCCATTCGCAGTTTCACAGTCTGAATTTGTTCATACTTACACACCTGCCC---------------------------------------------------------------------------------------------------------------------------------------------------------------------

>D-A8

GGCCGAGGTGCCTAAACCGGTTGAATTTGAGAGACTACCAGTCCCAGCACCATCTACCAATCAAAAGACTGTTCCAGCAGCAGTAGCTCCCAGCCAAAAGGGTTCTGATAACTACCTCGAATTTGATTTCTTCTAGTAATTATCAGCCCGACTGGTAATTTTTATGAAGTGGTGGACCAGCAAATCCTGAATTCAGGGATAATCTTATATGTGCAGGGTCCTGTTGTCACTGTCCATAACCTTATTATTTGTGAATCTGTACCTGCCCG----------------------------------------------------------------------------------------------------------------------------------------------------------------------------------------------------------------------------------------------------------------------------------------------------------------------------------------------------------------------------------------

>D-B10

CCGAGGTACTTGTATTCTTGACTCCTTACTGTATCTTTTTCCCCTTCCTCCAAAGGATCACTTTGATAAACGCGCTCATAACAAACTGGTGACAAACATTGTAGAGCACAATTCTCCTTGTCTACTGCTGTAGCAATGCACTTACTGACCCCATAATCCACTTACTCAATATCTGCATAACATTGGAGTTTCTTCTCTCTGATCTCTGCATCAGAGATTGGACGACGAGACTTAGCAAGTACCTGCCCG

>D-B5

GGCCGAGGTCGCCGCCGCCGACGGAGAGAGAAGAAAACGAAAAATGAAGACTATTCTCTCATCGGAAACAATGGACATCCCAGACGGGATAACAATCAAAGTGAAAGCAAAGCTAATTGAAATCGAAGGGCCAAGAGGAAAGCTTACCAGAAACTTTAAGCATCTAAATCTCGATTTTCAGCTCATTAAGGATGAAGAAACTGGGAAGAAGAAGCTCAAAATTGATGCCTGGTTTAGTTCTCGTAAAGCTACTGCTACTATTCGTACCTGCCCG

>D-B6

GGACCGATTCACTCTGCGTAGATTGACTGAACGCAGAAAACCTTCCACTGGCAGGCGATCGTGTTTTTCACAGGATTTTTCGTTACTCATGTCAGCATTCTCACTTCTGATATCTCCAGGTCTTGTCACCAAAAACCTTCCCCGATTGACAGAACGTTCCGCTACTGACACTTGAAAAAGCAGCTTTCAAGGTCTCGTCGCTTCGGTGAATCACTTGAGCCCTGATACATTTTCGGTGCCATGGAGCTAGACCAGTGAGCTATTACGCTTTCTTCAAAGGATGGCTGCTTCCAAGCCCACCTCCTGGTTGTCATCGCTCGATCACTTCCTTTTCCACTAAGTGATTGCACCTCGGC

>D-B8

CGGCCGAGGTACTGAGGGGAAGAGACGCTCACAGTGGAACAACGATACGGCGGCATGATTGTTTTGTTTTTAATACTTTTATTTTGTTTAGGTAGTGTGTTTTTATTTTTGTTGGGGGATATTTTGCTGGAAAGTTGACCTAAATGTGTTTGAATAATATTTGAATTATGGTTGGGGTGGTGTCATATGATATTGTACCTGCCCGGGCGG

>D-C1

TATATCTTTGTGATATTACAAATAACACTGTGTTAAATGCCAAGTGTTTTCTGGATAATTTTGAGTCTTTCCTCTCTGTCATGTCACACTTCCAATTTTTCTTCATTTCCTTTGTTGTTTAACCAAATTCTCAAGTGTAATGCTGTATTTGTTTTGGTTATAGTATACGTACCTCGGCCGCGACC

>D-C11

CCGAGGTACCTATCCACAGGCCATCTTACTGCCAAGAGTGATGTATACAGCTTCGGGGTAGTTCTGTTAGAAATTCTATCCGGTAAGAAAGCAATAGACAAGAATCGACCGATGGGGGAACATAGTCTTGTTGATTGGGCAAAACCTTATTTGACCAGTAAACGTAGAGTTTTCCGTGTTCTAGATGCCCGTCTTGAAGGACAATATTCACTCAGTCATGCCTTAAAGGTTGCTATCCTGTCTCTTCAGTGCATATCGATGGACCCCAAGTCAAGGCCAACAATGGACGAAGTAGTAACAGCTCTAGAACAGCTTCAGCAGTCCAAGGATGTAGCAAAAAATGATAAGAAAGTCCGGCAGGTAAATCAGCATAGTCGATCAAGTTTTGCGTTCAAAAAGTCCTGCAAAAGCAGCACGGAAGAGACTCCCGCGAAATCCAAGTACCTGCCC

>D-C2

AGCGGCCGCCCGGGCAGGTGGCGAAATGCGTGGTGCAAGGGTTAAAGGAATTGGGAGTGAGAAATTGTTCCATGTTTGCAATTAGCTATGGGGGATTTGTTGGGTATCGAATGGCGGAGATGAATCCGCAAATGGTGGAGAAAGTAGTGATATTGAGTAGTGGAGTGGGTTGCTCGAAAGATCAGAAGGAAGAGCAGTTGAAAAAGATTGGAAGAGATCCGGTTGAATTGCTCATACCGGAGAAACCAGAGGATCTGCATGTGCTCGTAAACTTATCAATTTACAAGTACCTCGGCCGC

>D-C3

CGCGGCCGAGGTACAAAGGTTGTGCTATTTTTAGAGCCTTTAGTTCCTGCAATTCCTTGTGAAGCCTTCTGTTTTCATCTGTCAGTGTCTCGCAGCATTTTTTTAGGAATTCACAGTCTACCTCTGTTTGCTTCAGTTTTGTTCTGGCTCTTCTGTTCTGAAACCAAACTTCAACTTGGCGTGGCCTTAAATTGAGTTCTCTGGCTAAATCCTGTTTTTGTTTAGGATTGAGAGTGCTGTGTATCTTGAAACTTTCCTCTAAAAGTGCAGATTGTGGTTTAGTGAGTCTAAGTTTCTTTCTAGCGTTAGATCCATCATCATCTTCGTCACTAATAACTCTGGAGGAAAGTCTTTCTACTTCAGTAGTTGTCTCTTCACTACCAACATCTCTCTCCCTTTTGACACTAGCATTTGAGTAGGAAGAGGCAGCACTATCTTGTCTGTACCTGCCCGGGCGG

>D-C7

CCGAGGTACCTTCACCATCGCCTCCTTCTACGACGACGCACCTCTCAGTTGCTGTTATCCTCAGAATCTGAAGCGTTCTGATAGATCGTCGACGGAGAAGTATAGATCTGAAGGTTTCTAGTATGGATGATCAATCGAGATCTACATCAGCTGCATCGCCGGAAAGTATGGATCTGGTTGGTAGAAAAACAGGGGTTAAGAGGCAAAGAGAAGCGAACCGATGTTCTGGTATGGGTTGCCGGAGGAAAGTCGGATTGATGCCGTTCCGGTGCCGGTGTGGGGAAGTGTTCTGCTCGGAGCATAGGTACCTGCCCG

>D-D11

CCGAGGTACTGTGTGAGAGGATGCTCTTGATTGAACTGGTAATCAAACTGCAGCTTCAATCTACAAGGAAGCTTGAGCAATTCCTAGAGCCTCTTCAACCGACGATGCATTTTGAAGTTTGGCTATGTCAACAGAGGGCTGGCTTCGTGCAAGTTTTGGAAGCAGTTGGAATTCCTCAGGACTCCCACTTTCAAGAAGAACCCCCTTCAGCTTACCAGAGTCGATCCAAAATGTTGCAACCTTTGGATCAAAATTCCCAAATTCAACGGCCTCCCCAACATTGTCACCAAAGAATTGCCACCATACTTTCCTAGAGCTTCCTTCATATTCAAAAACCCTTGAGTAGAAGTATGGTAAATAGTCATACGTGTGCGTGTGTGCAGTTAGCAATGATTTAATACAATGCTGTGCAGATTTACGAGCATGGTCAACGTGCTCCACCCGTGCAATTCGGTTGTATATCTTCAATGGAAATGCGGCAACATCTCCAATGGCAAAGATTCCAGGTATGTTTGTTCGGAACTGTCCGTCAACCTCACCTGCCC

>D-D12

GGTCGCGGCCGAGGTGCTAATTCTTCAACTAAACACTAAATAGGATTCAAGTTTCTGAAGACTTGGGGAATAATAACATTTTAAGCTTCATATTCTTCCCCTCTTAAGTTATCTGCAACATTTGTAATCTGCAGTTGTTCCACAATCCTAATTATCTGTGGAAGTGTTTTCCTTGTCTGTTATGTATTTCTTCACAAGTACCTGCCCGGGCGGCCGCTC

>D-D4

CCGGGCAGGTACAATTCCACAGGTAGAAGCACACTGTCGACGGTGAAAATGCAGAAGGGAGTAGAGTCAATGACTGTGGAAGCGATTCTGGACGAATCGACACCTGTGTCAAGTGTCACCTGGTCACCTTCTGTTGAAACTGCCAGATCATACTTGCTAGCGCCATTAGTAGCTAGAGTTGAAATCGGATCCTTCGTCGTTTTCAAGGTTCCAATAGGAGTGTAACTCGGAATAGCATGGTACCTCGGCCG

>D-D9

CCGAGGTACCAGAAGACTGCAGCTTACGGTCACTTTGGCCGTGATGACCCCGATTTCACCTGGGAAACTGTCAAGGTCCTCAAGCCAAAAGCTTGAGTGTGAGGTCTTGCATTACAATTTTTTGGCCAGTTGAGTTTGTTTCTACCGGCCATTATTCTTGTCGCGGACCAATAAACAAGCTTCATCATATCATACATTGATATCGATATTGATATTTGTTTTCAAGGCTGCATTGGTGCTGGAAGAAGACAACTAAGCGTTTCTATTCCGAGTCACTGAACATTTGTTATTTTCCTATTTCTTTCTTCACCCTTTTCTGCAGTACCTGCCC

>D-E12

CCGAGGTACATTTTATATGGCTACATGGTTTTAAACAACAGCCCTGGATATTTCTGCCATGTTAAGCAAGCATTCAATCTTTCAGGTCCACTTACCGTAGAAACATGAGTTTTGATGTATCCGTGGGGGAGTTATTTGAATGTTGGCTTCTCCTTTCTTATCATCTGTTAGGCTCGTAAAAGGTGTTTGTCACCTGTCAATATCTTGTAACTAGCATGAGGACCCTCAGATGTGTCTCTCACAGTTACACGCCAACCCATGGCTTTCCCAATATCTTGTAGTTCATCCATGATAGCAACAGAATCACGGATGTATGCTCGTCCACCTGGCCTCAATATCCGGTCCATTTCAAGCATGATTGTGGACATGTTGCATCTTTTTTGTTCGATGGAGAAGAGACCATTTGCGTGCAATAAGTCGTAGGTTCTTGGGTAAGTATCAAATGGTTCACACCAGTCATGCTTCACTCCAAGTAGTCCACGGTCAAAAATGACAGGTAAGGTATTTTTACCACTAACAGGGACAACATTCACCTGCCC

>D-E4

GGCCGAGGTACTGCCGCCCAACGCAATGGACAAACGATCCAAACATTCCTGACCAACACTGTAGTTACTTGTTTCCCCCGCATCCTCATGCTCAACTTCAGCACTATGCCAGACAGGTTCATCATCGATATCCAGCAACATTTTCATCAAAATAGCAAACAGCCTACTGATAAACTGTGGCAATTTCCTCATCATTCCAGGAGCCCTTTCCCTAGCCTCAGCCAAGGTAATAACAAATTCAATAGCCAAATGTCTTGTCCCCTCTTCTAAACTCTCAGCCTCGGCTATTTGCAACATTGCACCCACCACATCAACAAGCTGCCTCCTCAAAAATCTAGGCTCAGTACCTGCCC

>D-E5

GGCCGAGGTACGGTGACATTGTTTACCCAGACACGAGGAGTGTTCTACACAAGTTTGAGAAAATCGATAAATGCTTTAGCCAGGATACAGTTGAGGAAATTATAGAAGCTCTGGAAAGAGATGCAGCAGAATCCCATGATGAATGGTGCAATACAGCTCTTAATAAAATAAAAGAGGCATCTCCATTGAGCTTGAAAGTGGCTTTGAAATCAATAAGAGAAGGCAGATTTCAACCCCTTGATCAGTGTTTAGTTCGTGAATATCGCATATCAGTTAATTGGGTGTCCAAACGGATGTCTGATGACTTCTGTGAGGGAGTCCGGGCTAGATTGGTTGACAAGGACTTTGCTCCAAAGTGGGATCCAACACGGCTGGAGGAAGTTACCAACGACATGGTTGACCGCTTCTTTATCCAATTAGATGAATTAGAGCCCGAACTCAATTTGGCCACTGCTATACGAGAGCCTTCTATGTGATTCACGTCATGCTTTGCTCTGCATACGAGGCGTTCTATACTCTGCTGAATCCATCACAGTAATGGGATCCGCAGAAGACATTCCTATCAAGATGTTGGGTAGTTTTCATTCCCTTGGAGCAGATGTAGACGGCTGAACTGCACTACTGTCTGTTTAGGTACCTGCCCG

>D-E6

CGAGGTGGACTTTGGGATGGGCCGGCCGGTCCGCCCTAGGTGTGCACCGGTCGTCTCGTCCCTTCTGTCGGCGATGCGCTCCTGGCCTTAATTGGCCGGGTCGTGCCTCCGGCGCTGTTACTTTGAAGAAATTAGAGTGCTCAAAGCAAGCCTACGCTCTGTATACATTAGCATGGGATAACATTATAGGATTTCGGTCCTATTACGTTGGCCTTCGGGATCGGAGTAATGCAAGGAAAAATGGGAGAACATCAACAAGTACCTGCCC

>D-E7

GCGGCCGAGGTACAACTTATTACTTTGTGATAAGACAGGCTAGTTGACAACTCCATTCACATATTTCTCAGCATTAGAGGTGTTTGTGGAGGTAATCTCGACCTTTGGTGGACCTGAATGCCGTCTTGTAATGGCTGGTGAAGGTGAGTAAGAGAGTCGCTTCTTCACAGAACCAGCAGATCCCTTCGCTGGTGGTGTGCCATTTTCCACGCCTAATGGACTCTGCAAACGCGTTGTTCTTGCTTTTGCTGACTGAGTGGATGCCATATAACTTGGTACCTGCCC

>D-E9

GGGAGCTACGACCTCTGCCTCGACCCCTACCCCTACCATAGCTCCGATTGCGATCATACCCACCATCATCATACTCAGCCGGCATAAATCCATTTCCTGGAAAGCCTCTGGACCTTCCCCTTCCTCGACCACCGCGGCCTCTTCGTCCTCCACTAGGAGATCCTTCTCCATCATAGTCAAAATCTGTTGACACTTTCACCTGGTCTGCTGGCAATGGGGTTGGTACCTGCCCGGGCGGCCG

>D-F2

GCGTGGTCGCGGCCGAGGTACACAAATCATGGCTAAGTCCCTACCTGCCTCACCTCTCCATGGACTCACTCCACCTCATGACACTCCCACCAACAATAGATGAACTAATAGTTAGTTCTTCTGTTCTCTACAAGATTTGACACTATATTTGGCTTCTTATTTCTATAATGTATCAATTTGTACCTGCCCGGGCGGCCGCTCGA

>D-F3

TCACATCCAGTTGATTTAAAGTCTTTCAATTGATTGCAGTTAGCCCCAAGATTTTGCAGGTTTTTCAGCCATGGACGCTCAGAGAGCTTTGTTGGATGAACTGATGGGCTCAGCTCGGAATTTAACTGAAGACGAGAGGAGAGGGTTCAAGGAAGTGAAATGGGATGACAAGGAAGTCTGTGCATTCTATATGGTTCGATTTTGCCCTCACGATCTGTTCGTGAATACTCGTAGTGATTTGGGACCCTGCTCGAAAATTCATGAAGCGAAGTTGAAGGAAAGTTTTGAGAAATCTCCAAGACATGATTCTTATGTTCCCAAGTTTGAAGCAGAACTAGCCCATTTTTGTGAGAAATTGGTGATGGACTTGGATAAGAAAGTGAGGCGTGGTCGAGAACGCCTTGCTCAGGAGGTTGACGTTCCCCCACCTCCTCCAATATCAGCAGAAAAATCTGAGCAGCTGTCTGTCTTGGAGGAGAAGATTAAGAACTTGCTAGAACAAGTGGAGTCTCTTGGTGAAGCTGGAAAAGTTGATGAAGCTGAAGCTCTGATGAGAAAGGTGGAAATGCTTAATGTTGAAAAGACTACCTTGACTCA

>D-F4

TGGTCGCGGCCGAGGTACTCGCTACAGGAATACCTGTAAGCTCCACAAATCGTCTCAGCTCCTCACTTGATTGCGAACACCCACCACCCACATACAAAACAGGCTTCTTCGACTCGGAAATCAGCCTAACAATTTGTTCCAAAAGCATTTCATTAGGCAATTTAGGTAACCTAGACATGTAACCAGGCAACCTCATTGGCTGATCCCAATTAGGTATCACCAATTGTTGCTGAATATCCTTAGGTACCTGCCCGGGCGGCCGCTC

>D-F6

CGCGGCCGAGGTACTGAGATCAGAGAGAAGGAAATATTCAACTTCACAAAATGCAGTGTCCCTTACATGATCCGTTCTAAGATTACCCCCCACAAAACCAGACTTGATTGGCCTGATCAACGAACTTCCTCCTCGAGTAACTCTATTGTTCAAGAACATCCGCCCAGAACAATGAAAATTTTCATGTTTGAACATACCCGGTATCAGTAAGTCAGTTCTCCGCTCAAGGTCCAGAAGGAGTGATTTGCTTCATTTCTTCAACATGATGGAAATTTGTAATATAGGTGACTGCAACATCATAAATCAGTGTTCCGCGAAAATGATGGTAGTGGCATGGCCTCCTGTGGACGAAGGATGACAAGCACACCAAACATTCCCAGCATAGGGAGTACCTGCCCG

>D-F8

CCGAGGTACTTAATCTTTCCATCTGATCCAACATCAACCTCTCGGATCCACTCATCAAACTCCGAAGCCTCGAGCTTTTCACCTATACTGGTGAGGATATGCTTGAGATCCGCAACAACAACATAACCGGTAGAATCCTTATCGAGGACCTTAAAGGCATCACGGAGCTGGCGATCAAAGGGCTCAGGCTTAAGGTGTTTGGACATAAGCTCAAGGAAACGGTTGAAATCGAAGGGCGCATTGAGTTTCTCCTCGGCGATTATGGATTTGAGTTGAGCTTGGGACCTGCCCGGGC

>D-G1

GCGTGGTCGCGGCCGAGGTACTATATAATAAGACGGTTCACCATCCATCTGGTCACCTACTTATTATTCTTTATGCACTTTTTATCATTAAAATAAAAACTATATACCTTGTATACATCGAATATGGTACCTGCCCGGGCGGCCGCT

>D-G12

CGTGGTCGCGGCCGAGGTCTTAATAATCATAGGTTGAACTTGAGATTAATTTAATAGATTTGGTCATTTTTCTCTTTGTTTAGAGGGATGCCGTTCTTATTTTTTGTGTTGCATTTCAACATCTGTGTACCTGCCCGGGCGGCCGCTC

>D-G4

CGGCCGAGGTTTGGATTTTAGGCCAAAATTGATTATCTGTGGAGGAAGTGCTTACCCAAGAGATTGGGATTACAAGAGGTTCAGAGAGGTTGCTGACAAATGTGGGGCTCTTTTGCTTTGTGATATGGCTCACATTAGTGGTCTTGTTGCTGCTCAGGAAGCAGCGAATCCCTTTGAATATTGTGACTTGGTTACTACCACCACACACAAGAGTTTGAGGGGTCCAAGAGCTGGTATGATTTTCTACCGCAAGGGCCCTAAGCCACCAAAGAAGGGCCAGCCTGAAGATGCAGTTTATGACTTTGAAGACAAGATTAACTTTGCTGTTTTCCCCTCTCTCCAGGGTGGTCCTCACAACCACCAAATCGGCGCTCTTGCTGTTGCCCTGAAACAGGCTGCGACTCCTGGATTCAAGGCTTATGCTAAGCAAGTGAAGGCCAATGCAGTTGCTCTCGGTGACTACCTGATGAGCAAAGGATACAAACTTGTAACTGGTGGGACTGAGAACCACCTTGTCCTTTGGGATCTTAGACCTCTTGGTTTGACTGGTAACAAGGTTGAGAAGCTTTGTGACCTTTGCAACATCACTGTTAACAAGAACGCTGTTTTCGGAGACAGCAGTGCTTTGGCCCCAGGAGGTGTTCGTATTGGTACCTGCCCGG

>D-G7

GGCCGAGGTCTAGATTCATAAAACAACTCTCTCTGTCACTGTCGCCGGAGATTATTATTTACCGATCTGACCGGAAGCAGGTCACAGTGAACAATGGCGTTAGCTTTCGATGAATTCGGAAGGCCGTTTATAATACTAAGGGAGCAAGAACAAAAAACCAGATTAAGAGGCCTCGATGCTCAGAAGGCCAACATTTCCGCCGGAAAAGC-TGTTGCACGTATTTTACGTACCTGCCCG

>D-G8

CCGAGGTGCCAAGATTACTGTGATGAATACTTCAAATTAACCTACCCATGCACTCCTGGAGCCAGATATTACGGTCGTGGTGCCTTGCCTATCTACTGGAACTACAATTATGGAGCTATTGGTGAAGCCCTCAAGCTTAATCTCTTGGATCATCCTGAATACATTGAACAAAATGCTACCATGGCTTTCCAGGCTGCCATTTGGAGGTGGATGAACCCAATGAAGAAGGGTCAGCCTTCAGCTCATGATGCCTTTGTTGGCAACTGGAAGCCCACAAAGAATGATACTTTGTCTAAACGAGTTCCTGGTTTCGGTACCTGCCCGG

>D-H8

GGCCGAGGTACAGCAAGAAGACTACTCAAAACCATGAACTCAATCCAACATAGTAGATGCTTATTGGCTAGTCACAGCCATGCCCACTGACACCATGCTAATCTAAACTAGGTCCACATGCGACCAACTTTAAAACAAAGAACTTCAATATACTACCAATTCAAGTACCTGCCC

>E-A1

GAGGTACTTTTTTTGGTGATTGATTATTAGGATTCGCGGAAGCAAACAGAGGGTGTTGTGGAACAGGAACAGTGGAGACAACATCATTATTATGCAACCCAAAATCACCAGGAACATGTAGCAATGCAACACAATATGTGTTTTGGGATAGTGTGCATCCATCTCAAGCAGCTAACCAAGTTCTAGCTGATTCACTCATTATTCAAGGCATCAACCTAATTGGATAATTAATCCAATAAAAATATGTGAATTTTATATGTAAGACCTTTTAAAGTTACTTTCCTCTTTTTACTTTTTAAAATTTATCGTTTTCTTGTTGAGATGCATGAAGATGAAAATGATGTTTGTATTAGTTATTTTTACTGTTATTAATGAGTGTCGACTTGGAAAAAAATGTACCTG

>E-A2

CCGAGGTACAGCTTTAATTGGTATTTCAACTGGATCCTTGGATGGTTAGGAGTTGCTTTTAGCTTGGCCTTTTCGATCGGAGGAATTTGGAGTATGGTCACTAATGGACTTAAACTCAGGTTCTTTAAGCCCAACTAAGAGATAGATCATGAATAAAAAAGCGCGCTTTGGTATGTAAACTTGAATTTGCTAGTAGTAGTTTGTAATGTGTGTTTCTATTCTAGGCTTGAGAAAAGGTGAAGTGGAGAAACAACGCGTTCGTGCGTGTGTGTTGGTTGGTTCGGAGGAAGGAATAGGAATATGTATAAAGCCATAGGTACCTGCCCG

>E-A3

TTGAGTGGGGCTGTAATTGGCAAGCAGGCAGTTCGTCTTTCCTGGGGGCGAAGTCCATCAAACAAGCAGATGAGAAGTGATTCTGGTAATGGGGGCGGCTATTATGGAAGGCAAAATTATGGAGGATATGGATATGGCGCATCACAAAATCAGGATTCTGGCATGTATGCTGCTGGAGCAGCCTCTAATGGCTACGGGAATCAGCAGCCTGTTAGCTGAACTGTAGTCAACCGCTGCTAAATGAGACATTGCCAAAACGCTCTTTCTTTGGGGGTTTTAAATTTAAATTGACTTAAAAGCATATAGTGTTTGATGAGAATTTTGTAGCTGGATTTGATTTATGCTTAGTGAGTCCAAGCTATGTGAAACGTTATACCTAAAAAAAAAAAAAAAAAAAAAAAAAA

>E-A8

AATCATTGCCACTTAAGTGCAGTAACCTAGCACATAGAGACAGACTACCAATAGCTGGCGCTAGCATACAGCAGCAAGGCATGGAAGAGGAGGGAGATTAGGTCCTCCAACATGTGCTCCGTCGACACAAACCAAGCTTGTCCATCGGAGTTAGTCACGTGTCCAAATGGAAAATAATGCATAGTCTAATGTCTTTTCAGAGATCAAGAAGAACAGAACCAGGGAAAAGATAGAAATCAAGCCTCATTATTCAATTGACCCTAATTGTAAGGGAATAGAGCAGCGCCTACTCAGATGCAGGTCTAAATGTCAATACATCTGTCAGTTGGACAATGAATATTATCCTACCAGAAGAACTCGAAAAGCTGGATTCAGCATGGGACTAGTAGCTGAAGGAAAAAAGATCAACATAATCAAGAAGAGAAGTAGGAAAAACTTCTGGTAATTTCTTCTTCTTCTATTCAAAAAGGAAGGATTGGGAGCAGCTAATAGAGTCTGGGAACCAAAACAGCAAGCCCCAGATCATGGCACACTTCATTAATAGGGGTCGGGTAATTACATGCTGTCGAGGAGTAAAGAAG

>E-B7

CCGAGGTACACTGCAATCATTTGGAAACCAAAAGGATCTCCGGCTTTTAGCTTTACTAGGTGTGTTAACATTTGACATAACGGTTACTGCAGCAGCTATCTTAGCTGTCTCACGACCTTTCTTGTCAAGCTGAATCAAACTCCTAGTTGCCAAAATTGCCTGCGGATTCAACTCATGTGAAGCCCTTGCATACATCCGTTTCACTGCCACTGAAGCAGCATCCTTCACATCCTCAGCATTCAAAGGACCCAACAAACAATGCCCCAAGATCCTTATCACAGGCTGCAATATTTCCCACGGAAGTGGAATTCTTGCACCCTTTGGCCTTAATTCATCATATCCATTTTCAATTCTCAAATTCTGCATCGCTTCAGAAACACCCTCCAGATCCCCATTACTAAGTTCAACAGAATTCTCAGAATTTCCATCAGTTTCATCAAATTCTGACACGCAAGGACAATCTTGCCCAGCCCAATCAGCAGCAAATTTACAAAAATCCAACTTAGACCAACTTGGCATATGGGAAATCTGTTTATAGTAACAATCTAGAGCTACCCCAACAATGCAAGCACGTTTAGTTGACTTCACACCTGCCC

>E-C11

CCGAGGTACTGGTGCAGGCTTGGCTGGCCGATGTTCTGTGAACTCGGAAGCTTTCGGAGACTCAGATAAATGAACCTGTGCGAAATCAGTAACCATTTTCTGCCTTTGCTCATCCTCTGTCATTCCCACTTTCTCTTTTGTCATCTGGCCTACTTCCCCTGCTGCCTTAGCAACCTTACTAAAAGCGCCGGCTACCCAAGTGGACCCAGTCAGAACATACCGATTTTTCATAATGGCTGATCCAGCGCTACTAACTGTTTGCTCAGCTGCAGAAAGTGCTGATTTTGCTTTATCAGAAACTTGGAGTTTCTGATCCACCTCTCGAACTTTGTCATTAACAATGGAAGTACCTGCCC

>E-C5

GCCGAGGTCTGGTGTAACCATTGCTCATGGAGGTGTTCTTCCTAACATCAATCCAATTCTTCTGCCTAAGAAATCTGATAAAGTTGGAAAAGAACCTGCTAAATCACCATCTAAGGCTACCAAATCACCACGAAAGGCTTAAATTTGTGAAGAACAACACTCTACCAAAGTTGCATGATTTTAGGATTTTATGAACTTGTGTTTGGTTCAAATAAATCTGTTAATGTATGTAGGTTTGGTATCTAATTTCTGCTTGTTGTGTAGGGGGCTTATAAGCCTTAGGAACAATGTACCTGCCCGGGCGGCC

>E-D1

GGCCGAGGTACCACTACTTCCATTGAGGACAGTCACATTGCCATCTGAATCAACGTCCACGATAACTGAATCACCTTCTTTAATCTCATTTGCAAGCATTTTCTCAGCCATGCTGTCCTCTAAAAGTCTCATAATAGCTCTTCTCAGAGGTCGTGCTCCATAGCTAGGGTTGTATCCCTCGTCAACCACCCTGTCTCTAAACCTCTCTGTCACTTGAAGTTCTATCTCCTTAACTTTCAACCTCTCAAAGACCTCCTTAAGCATGATATCAGCTATCTCCTTAACCTCTAACTTAGTGAGCTGACGGAATACAATCATCTCATCCAATCTGTTCAAAAACTCTGGCCTGAAGTACCTGCCCGGGC

>E-D5

GGCCGAGGTACACACTTGACTTAATACTACAGGTTTGTTCCCAACCAGAGCATTTAACCAGAGATCTCAATGGACTTGACCTCAGGCTTCTTCACCTCTTCCTTTGGAACAGTAACAGTAAGCACTCCATTCTCCATAGACGCCATAACTTGATCCATCTTTGCATTCTCCGGAAGTCTAAATCTCCTCATGAATTTCCCGCTGCTTCGCTCCATGCGATGCCACTTATCATTCTTATCTTCCTTCTCCACGTTCCTCTCTCCGCTGATCTGAAGAACCCTATCCTCCTCGACTTCCACTTTGACTTCCTCCTTCTTAAGCCCTGGAAGATCAACCTTGAACACATGAGCTTCTGGAGACCTGCCCGGG

>E-D7

ATCGCAGTTGTTGCCGGGTTTTGCTTTAGTTTGGCTTTTATGCTTTTTCTCATAGTTACATTACTCTGGGCGTGAAAATTTTCTGCTGTTCATCCCTCCACGTGGGAATCTATCGAGTTCTTCTGCTGAAGGTAATGCTTGTTCAGGAGATGCAATCGAAACCCTCTTGCTGTTCGAGTAAATCAGCTTGTCAATGAGAAAACATTTCCCGTCTGAAATACTGAATTCATGCTTTGCAGTTTGATAAAAAAAAAAAAAAAAAAAAAAAAAAAAAAA

>E-E3

AGGTACTGGATTTCCTTCCAAAGAAGAGGTCACTGTTGAAACAGGGGAGGAAAATGAAAAACCCGTATTCGCAGCTGATTCCGTGCTGTTTGAATATCTTAATGGAGGGTGGAAAGAGCGGGGGAAGGGAGAACTAAAGGTCAATGTTTCTACAACAGGGGAAGGAAAAGGTAGACTTGTTATGAGGACCAAAGGAAATTACAGATTGATCTTGAATGCCAGCCTTTTTCCAGAAATGAAGCTTGCTAATATGGACAAAAGAGGGGTCACTTTCGCTTGCTTGAATAGTGCTGCTGACGGAAAAGGACTTTCTACTATTGCTCTGAAGTTCAAGGATGCCTCCATCGTGGAAGATTTTCGTGCTGCTGTAGTGGAACATAAAGGTACCTGCC

>E-E4

AGGTTCAGAAGATACCTGTTTTCATCCATTGTGCATATGGTCATGGAAGGAGCGTTGCAGTCATGTGTGCACTGTTAGTAGCTCTTGGTCTAGCAGAAGACTGGAAAAATGCTGAGAAATTGATCAAAGAAAAACGGCCATACATACGAATGAACGCTCTGCATCGCAAAGCTTTAGAGGAATGGTCGAAGGATCGTCTCTCTTCCCCAAAAGTTGGAGTTAGTTCCGTGATTCTGTCGAGTGCTAACGACCGTTCCTGATCAATTGACTGCAGTTGCAAGACCAATTAATTATATAGATGATTGGCTGTTTGTCTTCACTTTTGATTACACACTATACTTGATGATTGAGATTTTTGCTTCTTTTTATCA

>E-E5

CGAGGTACACACATTGATTAGTTCTTCAAGGAAAACTGCCAGAATCTACATTTCTTAACGATTCAGCACGGAAAGATAAAGTCGATCCTTTTGAACACTCTGATTGATTCTTATTCCTGAAAGAAGTTCATTGACATTTAACACCAAAACCAAACTTCAAACTGTCCTTCAACGGGGGATAAGATATGAGAATTACTGTCCTTCAACCTTGTTCTGCCACCATTCCATGAATTTAACCTGAGCAGCCATGGTGTCAAGCCTGTATAGCTGCAGCCAGTGATTAGCAACTTCCTGTCCAACCTGCCCG

>E-E8

ACTGGCGATGGAGGAGATGGAGCTGGTTTCGGGGGATTTTGGCCTGTCTTGACAGGGTAAGAAACAACTGTAGCAATACCACACAAACCCTTTGGGTTAGCAATGTTACGCTGCATCCTGAGGTAGCCGTGTTCTCCCCAGGAAGCACCCCATGAGTTCCTCACAATCCAATAATCCATGCCATTTTCGCTACCATATCCCACTGCAACCACACCATGGTCCACTGCTGCGCCACACTTCCCAGTAAAGATGCCCGATTTATAGTGCTGGAAGTCTTTGCCACCAGCTTCAATAGCAACGCTAACAGGCTGACTTGCAATAGCCTTTTTCATTGCGTTTTCATCATTAGCAGGAACATCTTCATACCCGTCAATGGTGACAACCTTGGCATTTTTCCTTGTTAGGTCACATCTTCCATCCTCGCCAGTATATGGGTAATCTTCCTCAGTGTCTAGTCCTCCATTTTTGATAACGAATTCAAAAGCATAGTCCATGAGACCACCGTCACAACCATTGTTAGAAGAAGTATCACAATCTACTAGCTCCTGCTCGGATAAAGAGATCGAGTCTCCAGTCTTTATTTTGTTTACTGCTTCAATGGCAGCAACTGCTGAGAAAGCCCAGCAACTCCCACATTGTCCTTGATCCTTAACACCAACAAGCACACCTTTCTTCACCCAATCAACAGATTCCGGCAACCTATCTCCGACCTTCGGAGCATACCGATCACTTT

>E-E9

GGCCGCCCGGGCAGGTGGCGGCTATGGTGCTGGTGGAGCACCCGGGGGTGGATATGGTGGTGGAGGTGGACATGGAGGCGGTGGTGGTAGTGCGTATGCTGGAGGAGAAGGAGGAGCCTCAGGTGGTGGATATGGTAGTGGAGGAGGAGCTGGTGGTGGTGCAGGAGGAGCACATGGAGGAGCATATGGTGGAGGCGGTGGTAGTGGTGCCGGTGGTGGTGGAGCCTATGCCGGTGGAGAGCATGCTGGTGGTTACGGAGGAGGTGCTGGAGGTGGTGAAGGTGGTGGACACGGTGGTGGCTACGCTCCTTAAGCGGTCATTCTAATCTAAAAGTATCCATCTTCTTACCTACACTATTAAAAAAATAATGTACCTCGGCCGCGACCAC

>E-F12

TATGGAAGAAAGACATGTATATGTGATATTAGATATTGCCTAGTGCTAGTTATATATGAAATGAACTACTAAAGATATATTTTTCTAGGGGATTCTAATAGACTAGAAGTCCTTCCGGCCCCTTGTGACTGTGAATTGAATGACTAAACGGATGAAATCAAGAAATAATTCAACTAACAGTTCGAACCAAGAACAAGAAATGGAAGAATGAAAGTCGTATGGGTTCACAAAGACTCTGTGGCTAAAAAGTAAAAAAGATATATCGAAGTTGTTTTGATGATTCAAGAATCTTATTACTTCAATCCGAAGTTCTTAGTTACTTCGACTGGATGAGTCCTAGTGAGGGAATAATTAAGTCATAATTCATTGGTTGATTGTATCATTAACCATTTCTTTTTTTGGTACCTCGGC

>E-F3

GAGGTACTTCAAGGCGGTCACCGCGACGCTTCCGTCGCGGCGACTTAGCCAACGACACGTGCCCTTGGGGGCCAAAGGCCCCTACTGCGGGTCGGCAAGCGGACGGCGGGCGCATGCGTCGCTTCTAGCCCGGATTCTGACTTAGAGGCGTTCAGTCATAATCCAGCACACGGTAGCTTCGCGCCACTGGCTTTTCAACCAAGCGCGATGGCCAATTGTGTGAATCAACGGTTCCTCTCGTACCTGCCC

>E-F6

TTTTTGGGTGGAGGTTCCTCATTTGGATTATAGAATAGAACCCATTTACATGTATTGAACAATGATTCATAGAGTAGATGGAAATATGTATATTACTAGCCCAGTGATTCACATATATTTGGATGACTGAGTAGCCTATTGTCTGCTGCTCTATTTTAAGATGGTTTACTCTATTAAAAAAAAAAAA

>E-F8

GTGCATCTCTGAAAGATGAATTATAGCCAGGGTCTTTTGCAGAAACACCGAACTCTGAGAATACCACTGGCATGTTGAGATAGTTCTCTGCGTCATCAATGTGTGCTTGCATCCATGTCTTGGTGAATCCAATGTGAGCATCAGAAATCTCTTGTGAAATCCAAGAGTCGGGATATATGTGAACTGAGGCGAAATCAGCTCCAAGAACCTGATGATTTCTAATGAAGTCAGTTCCCACTTGTTGAGCATAGGAGTTTGGGTTGATCTGAGCTTTATTTGGAGTCGAGGGACCATAGAAACCTTCCAGTCCAATCTGCACCAAATGCTTCGGATCTATGGTCTTCACATAAACAGCCATTTCTTCTATCCAAGCATGCAGTTTATCCCCAGAAGGATCTGATTCACATCGCGGCTCATTCATCAGTTCCCAACCAAAGATAGTAGGGTCATCCTTGTATGTAATGTTGGTAAAGGTATTAACTCTATTGAGCACAGCCTTCACATGAGCCTTGTAGTAGCTTTTGAGTGTGGGATGAGGAGGAAGGAAGTCATCGTCAGAAGTCAAATTCAGGCCAGCAGCTTTACCCCATTTTACGTACC

>E-G1

CGAGGTACATGGCTGCACCATTGAGGATGAGGCCTTCATTGGTATGGGGGCCACACTGCTTGATGGTGTTCATGTAGAGAAACATGCCATGGTTGCTGCAGGAGCCCTTGTGAAACAGAACACAAGGATTCCCTCCGGAGAGGTATGGGCAGGCAATCCCGCTAAGTTTCTGAGGAAGCTAACTGATGAAGAGATAGCCTTCATTGCTCAGTCAGCAACCAACTACTGTAACCTTGCTCGTGTCCATGCAGCTGAGAATTCCAAGTCCTTTGACGAAATTGAATTTGAAAAGATGCTTCGTAAGAAGTATGCCAAACGTGATGAGGAATATGATTCTATGATTGGTGTTGTCCGTGAAACACCTCCCGAGCTTGTACCTGC

>E-H12

ACTAGTTAATTAATACTTATAATAATGCATTAACAGAAAACATAGGATTAACATCCACTAATTAATCTCCATGTTTCTGTAGCATAGCAACTCATACATTAAAAGGAAAATACGTTATTTACATATAACATTATTCAGATATCGCTCTGTCACGATCCAAAATCTTTTAGCCTTTCATTTCACTGATGCTTGGATCGGGTATCCCCACTGTAGAGCCAATCATAAACGGTGGGTGCATTAGGGTGAGAAGGTTTGTCAAAGACTTCAGCACCAATTCTCTTGGTTGCACTGTTGCTCCCCGGATTGAACACGCTCCTCCACACATTATCTTTACGCGCCGCCGTTGGCGAGTAAGTGTTTGGGGTCCCCGGGCTTCCCGGTGTCCTTGGGCTCGATAAACTTGAGCCGGAGCCCGGCATAGATAAAGACCTTTGGTAACTTTTACTTGCTCCTTCTCCTCCTTCTCCACCATGATTTTGGATTAGAGTAATGGACTTTCTAAGTTTGCCAAGTCCTTTATCAGGTTTAGGTCCAGCCATAACATCATCCCAAAGCTTTTCAATTAACAC

>E157

CCGGATCAACGATGTTCTTGGTAGATTGGTTCTATGGAATTCTCGCAACCCTCGGTTTATGGCAGAAGGAGGCGAAGATCTTGTTTTTGGGTCTCGATAATGCCGGAAAGACCACCTTGCTCCATATGTTGAAAGATGAGAGATTGGTTCAACATCAGCCAACACAATACCCAACGTCAGAGGAGCTTAGTATTGGAAAGATCAAGTTCAAAGCATTTGACTTGGGTGGTCATCAGATTGCTCGCCGTGTTTGGAAAGATTATTATGCTAAGGTGGATGCTGTTGTATACTTGGTGGATTCCTATGACAAAGAAAGGTTTGCCGAGTCTAAGAAAGAGCTGGATGCTCTCCTCTCTGGCGAGTCCTTGGCCACTGTGCCATTTCTTATATTGGGTAACAAGATAGACATTCCTTATGCTGCCTCTGAAGACGAACTGCGTTACCATTTGGGGCTAACAGGCGTCACTACTGGCAAGGGGAAGGTAAACCTGGCAGAATCCAATGTCCGTCCACTTGAGGTGTTCATGTGCAGCATAGTCCGCAAAATGGGTAATGGGGGAAGGCTTCAGGTGGATGTCTCAGTACCTGCCCGGGGGGCCGC

>E158

CGGCCGAGGTCATGTTGATTCCACCGGTGATTAATGGGCTCGACGAGCCCACCAAAAACTAGCAAAAACGGCGGCAAGGAACCCAGTTCGTAGATTCGCCGTTGTCTCTGTAACTCCATCCATTTTCTCTATCTTTTTCCGGTAGTTACCTTCTCTCCATTTCTGCAAATCCATTACCATTACGCCGGTGTTGAAGTAACATGGGTTTCGTGACCCGAACACTTGAGGTAAATCCGGGTCAGACCAGAAGGAATCGGTGAAGTATTTTGTGAAATTTGCATGGCAGTATTCCGGTGCTCCGACCTGCCCGG

>E162

GCCGAGGTCTTGTTGATCTTCACCCTTGCTGTCTTATTCTTGTGGTCCAATGCGACAACCTTTATTAACAAGAAACGTCCACACATCCCAGAAGTTCAGCTTCCGGAGGAGCCAATACTAGAGATTGCTTCTGCTCTGAGTATTGAAATTAACCGTGCTCTTAGTATGTTGCGAGAAATCGCAACTGGGAAAGATCCGAAAAAGTTCCTTGCTGTCGTTACTGGTTTATGGATCCTCTCAGTTGTCGGCAATTGCTGCAACTTTCTGACATTGTTCTACATATTATTCGTACCTGCCCG

>E169

GGCCGAGGTACCTGTTGCCCACCTCTAGCTAATCCAGTCAGGTCATTCTTGGTCTCTCCTTCAGAAAAGTGCTCAAACTTGGGGAGCTTCACCCCAGCAATATTTTCCTGCCGTGATCGAACTTTCAGAGTAGCAGTCTGGACATTTTCAAGGACAACGTGCTTGATGTTCTCACCAGCAGCATATTTGGCCTCTGTCAGAGCAAATGAGGAATTTTTCATGACATCTCCCATCGATTCCTTTGTTGATACTATTTTCTTTAAAATCTGACGGAACTGCACAGTCAAAGCATCACTTTTCTTTTTTAGCAAAGCATGGCCTCTTGTTGCTCCAACAAGGCGAGCTTTAATAACTCCAAGCATCGTAACTGTTGGGACGACAACCAAACGGTTGGTTTGCCCTGACATTTTTCCAAGCTACTTAGATGTAGATTTCGCCGGAGNAAGGAAAGAGATCACCGGAGCTTCAAACAAACTCAGCAGAAAAAGAGAATTGGGTCAACTACCTGCCCGG

>E172

GAGGTACTCCAAACCAATCTTTTCTGCCTCTCCAACATGGCTGAACATCTCCAAGCATACAGATCTTCATCTTAGGGCGAGGAATGTGTGGTAACTTAACAGATCCACTGAAACGCTTGTCCTCTTGGGGATCATAGTTCTTCAATCCAATCAGGAGCTCAACGGTCTCGCTGAACCTCCTCCTCTTCTCCGCTGAATCATTCTTAATCACAGAGATGGCTTCTACTGAGGGCCTCACTACTGAAGCTTACTCATGGCTCAAAAATCTTCTCCTCCTTCAAACTCACCTGTAAGGAGAAAACCCTCGATTTAGGGTTTGACCTGCC

>E175

GAGCTTTTCTCGCCTTTAATAATTAGAGTTGGATACAGCTATTTTCTTGTATACTGATGTGTTGGCTTGTGTCGATGAGCTTTGTATTTTTGAATAATTCAGAGTATTTAACAAAAAAAAAAAAAAAAAAAAAAAAAAAAGCTTGTACAGTACGGTCATATGCCTTGGCTACCCATTCTCTAACTTCAGTGTCGATAATTGCTGCTGTCTTGCTACCATAAGGCTTTGCGTCAAATGAATCTTCTCTTTGTGGAAACGAAAGGAGACCGACCTTGTCACTGAAACCATATACTGCTACCTGGGCATAAGTCATCCTGGTGACTTTCTCTAAATCATTTTGAGCTCCAGTCGAGATCTTCCCAATCAATACCTGCTCAGCAGCTCGGCCACCGAGAGTCATGCATGTCATATCAAACAGTTGTTCTTTAGTCATTAGAAGGTTCTCACTAGGAACATACTAGCAT

>E176

GTACCTGAAAATCCCACAAACAGGGTGGTGGGGAAAGGCCATCATGATTCGTAATCAACCTCTTTTGTGGGTTTTGTCCATTGGATTCGAGTTTATGGAGCTTACCTTCCGCCACATGTTACCAAACTTCAACGAGTGTTGGTGGGACAGCATTATTCTTGACATATTGATCTGCAATTGGTTCGGCATATGGGCTGGAATGCGCACCGTCCGGTATTTTGACGGGAAAACATACGAGTGGGTTGGCATTAGCAGGCAACCAAATATCATGGGCAAGGTCAAGCGAACTCTTGGACAATTTACGCCAGCTCATTGGGATAAAGATGAATGGCGTCCTCTTCTAGGTCCATGGCGGTTTATTCAAGTCCTCAGTCTTTGTGTCATATTCTTGACTGTGGAGTTAAATACATTCTTTTTGAAGTTCTGTCTTTGGATTCCTCCAAGAAATCCTTTGATAGTGTATAGATTGGTTTTCTGGTGGTTAATTGCGTTACCAACAATCCGTGAATATAACTCGTATCTACAGGACAGAAAACCAGCGAAGAAGGTAGGAGCATTTTGCTGGCTTTCAGTGGCGATCTGCATTGTTGAGCTCCTAATCTGTATCAAGTTTGGACATGGATCATTTCCAAACCCAATGCCCAAATGGGTGGTAATT

>E179

GGCCGAGGTACAAATGATCAGTCAAATCCGACAGATATGAGTAAGCTGTTGGCAGATCAATCATTTGTGTCATCAATCCTTTGCCTCACTTCCAGGTGTTGATCCAAACGATCCTTCTGTCAAAGATTTGCTTGCTTCCATGCAAGGGCAGTCCGAGAAGAAAGGATGAGGACAAATGATAAGGAACAGAAAGGAGGACAAGAAGTAAAGATGCGTACCTGCCCGGGCC

>E181

TCATTAAAAAAGAGAAGTCGTCGTATGGTTTTGTCGACTACTTTGATCGCCGATCAGCAGCCCTTGCTATTGTGACTCTTAATGGAAGAAATTTGTTTGGACAGCCTATAAAAGTTAACTGGGCATATACTAGTGCTCAGAGAGAGGATACCTCAAGCCACTTTAACATTTTTGTGGGTGATCTCAGTCCAGAGGTTACAGATGCCACATTGTATGCATGCTTTTCAGTCTACCCTAGCTGCTCAGATGCAAAGGTTATGTGGGATCAAAAATCAGGCCGTTCAAGGGGATTTGGATTTGTTTCCTTCCGTAATCAGCAGGAGGCGCAAAGTGCAATAAATGAGTTGACTGGGAAGTGGTTAGGAAGCAGACAAATCCGCTGCAACTGGGCAACAAAAGGCGCTGGAGGAATTGATGAAAAGCAGAATTCAGATGCGAAAAGTGTTGTTGAGTTGACAAGTGGAACATCAGATGATGGTCATGACAAGGCC-AATGAAGATGCTCCAGAAAATAGTCCCCAGTATACAACTGTTTATGTTGGCAACCTTTCTCCTGAAGTCACTTTAGTTGACCTCCATCGTCATTTTCATGCTCTTGGAGCTGGCGTTATTGAAGATGTCCGTATTCAACGGGACAAAGGTTTTGGGTTTGTGAGATACAGTACCTGCCC

>E182

CGAGGTACAAATGGACTAGTCAAGAATTTGGTTGCGGGTAATCTCAACCAAGTCTCATCATTAGTCCGCAACATTCTTCTTATGGCCCGACCCGTCTCTAGGAAAAACGTCTTCCCCTCTGAGGCCAATGCCAAACGTGGGAAGCCGTGGCAATTCTCCCGAGAAAGAAAATTAACGTCAAATGAAGAAGATCACAACTTATTCCCTTCGTGGATTAAACGAAAAGATAGAAGGTTATTGCAAGCATCAAATTCGACTAAAGGAGTAATTACAGACGTTGTAGTAGCTCTTGACGGAACTGGTAATTTTACCCGAATTAAAGATGCAATAGACGCAGCACCACAATTGAGTACCTGCCCGGGCG

>E186

CGTGGTCGCGGCCGAGGTTGGCTCTCAAACAACATTGCAAACAAGGGTTGAACACGAGATCCATGATTTTTGCAGCTATATTTTTGTCAATATAGACTTCAAACAATAGTCTAGAGCACTTCGTGCACCTAGCTGAATAGGATCCACTTATCACTATTTTTTATTTCAGTACCTGCCCGGGCGGCCG

>E187

TCGCGGCCGAGGTACCTTGTAGCAGAAAGGGGACTTTCATTTGATGAAAAGTGACAGAAAGCTTTTGCTTCTTCGCTGTTCGCAAATACTATATCTGCATAATTTGCCATGATTTCCCAGTAATCATCATAATGTCTCTCAATGCAGGACACATCTGATGCTGTGATAGCAACTAATGCTCCATTCTTGCGGGCTTCCTTGCAGGCTTTCGAAATTGTTCGGACTGTATCAGGAAGTTCAAACAAGTATCCTTCCACAACCAATATATTTGTTTTAGTAATTGCCTCTGCCAAGCATGGGTCATAGTTTATCCTTGACTAAGGTGGAGTCGCCAACCAAGTAGCTGATGGACTAGCTACAAATGGAAACAAAGCTGAATACCTAAGTATCTTCTTTTTAAATATCATCTGTTGCTGCCTCCTTGTGTATCTGTACCTGCCCGGGC

>E188

TAGGGCGTAGACACCGCCACCGCCGATGAATTCGACATCGAACAGCCACAGAACCACCCTCAGCCTCCTCCAGAACCACACTTCGACATTACCGAGCGTATGCCTCATCTCACTGACCCTAAGGTTGTCAGCGCTATGCGATCAGCCGTTGCTGACGTGTCACAGACCCGGTCAATGCTCAAAACCCTGGGTGAGCGGCCTTTTCACGAGCTTGTTGATACTGCCAGGGTGAAGTTAGCTGAGATCGATGCCGATATGTCCAAGCGTCTTGAGGAGATTGTCTTGTCTCCACGTCCGCCGGAGATGGAGAGGCAGGATTGGAGATTGGACATGGCGATTAAAGAGGATGAGTGTCGAAAGGGTGTGGAGAAGGAGAGAGAGGAGTATAAGGCGTTGATAGCTTTGGATGAATTGCATGAGGCCTATGAGAAGATGTTGAAGGATGCGGAACAGAGGTTGGAGAAGATATATGAGACTGCTGTTGCTGGTGGTGATGTGGAATCAATTGGAGAGTCTTCTGGTGAGAAGAGTTCTGAGTTGAAAGAGGAAGTGAATGAGGAAGTGATCAGGATTTTGCAGGAGGCATCAGGCAAGAGTGTGGAAAGGGTTGATTTGTCAGGCAGGCAGCTGAGGATGCTGCCTGAAGCTTTTGGGAAGATTCACTCCTTAATAGTGCTAAATCTGTCCAACAACCAGCTTAAGGTGGTTCCTGATTCAATTGCGAGCTTGGAACACCTTGAGGAGCTGCACCTTTCTTCAAATATTTTGGAATCGCTGCCAGACTCCATTGGTCTGTTGTGTAATTTGAAGATCTTAGATGTCTCTGGAAACAAACTTGTCGCTTTGCCAGATAGCATTTGTCATTGCAGGTCGTTAGTGGAATTTGATGCAGGCTTCAACAAGCTTTCTTATTTGCCAACAAATATTGGCTATGAACTGGTAAATCTGCAAAGGCTTTCACTTTCTTTTAATAAGCTCCGCTCGTTACCCACTTCCTTTGGTGAGATGAAGTCCCTGCGCCTTCTAGATGTGCACTTTAATGAACTACATGGGCTTCCACTTTCATTTGGGAACTTGACAAATCTTGAGATCGTCAACTTGAGCAACAATTTTAGTGACCTAACTAAGCTTCCTGACACAATTGGTGACTTGATAAATCTCAAGGAACTTGATCTGAGCAACAACCAGATCCATGAACTGCCTGACACAATTAGCCGGCTTGACAACCTAACTGTGCTTAAGTTGGATGAAAATCCTCTTGTGATACCTCCAAAGGAAGTCGTAGTTGAGGGGGTTGAAGCTGTAAAGGCTTATATGATTAAGCGGCGGCTTGACAATATTATTGGCAGGAAGGACCCAGGAAAAATTATGCTGAAGGAGGTGGGCA

>E192

CAAGGCCTATCCACAAGAGAAGGTTGGTGTGTTTGTCCGTCGAGGTAAAGGTGGACCACTTTGCGGTGGTTGAGTATAGTGAGTTGGATCCTTCACTCTCTAGTGCAGTAAACCAGGAAACAGGACGTCTTCGCTTTTGTTGGAGTAATGTCTGCTTACATATGTTTACAGTAGATTTCTTGAATCAAGTGGCAAATGGCCTTGAGAAAGACAGCATTTATCATCTCGCAGAGAAGAAAATCCCCTCAATTCATGGTCAAACAGTGGGATACAAACTGGAACAACTCATATTTGATGCTTTCCCTTATACACCTTCAACAACACTATTTGAGGTTGTTCGCGAAGAAGAATTTGCTCCAGTTAAAAATTCCAACGGGTCAAACTTTGACACTCCTGATAGTGCTAAATTGCTTGTTCTTCGTCTCCACACCCGTTGGGTGGTTGCAGCTGGAGGCTTCTTGACACATTCTGTGCCCTTATATGCCACAGGTGTGGAGGTTTCACCTCTTTGTTCATATGCTGGCGAAAACCTGGAATCTATTTGCCGTGGAAGAACATTCCATGCACCATGTGAAATCTCGTTTTAGTTTCGATTTGATTTCATTAAGTAAAGTCCTGAAAATTTTTTCTCCAATTTATTTTATTTTTTTTCCATTTTCACGAGGGTTGTCTTTTCATACTGTGCTGTAAGCG

>E195

TCGCGGCCGAGGTACTGGAGGAAACGGACAAATGTTTTTCCGACTAATGCATTGGAAGAACGACAAGGGAGCAGTTCATCCATTAATGATGAAGCAGTTCAAAGAGTAAGGGAGATCCATCCATGCATGCAGCGTCTTCATAAATTGGAAAATTTATTGGAAGAAATAAACAAAAAGCCCGCTGAGATCCCAGCAGAGAAGGATCGAATGCTTCATCAGTCACTTAACAGGATCAAGTCTGTAGAAGTTGACCTAGAAAAAACAAAAAGGGTATTGCATGCGACGGTATTGAAGCAACTTGAAATTACAGAGTTATTGGACAACCTAAAAGAGTCGAGCTTTCATAGGCGGAGATTGTGGTGTTGAATATTGTAAGGTTGGAGATTTGAGTACCTGCCC

>E196

GCAGGTCGGCGGAGAAGAAAATGGCATCGACGCAGCAGCACCATTCTGGAGATCAGCAGGGATGACTTACATAACCTATTCAAATCTCTGTGCTAATCTTGTTAGGAATTGCCTGAAGGAACCTTACAAAGCTGAAGCCCTTTCCCGTGAGAGGGTCCATTTCACCATCTCCAAATGGGCTGATGGCAAGCCTCAGAAACCCACCATCCGCTCGGATTCTCCTGAAGAATGAGAACAAGGTTGGATCTCAGGCTTTATTTCTTCTCTCAACTGAATTTTGTACCTCGG

>E198

TCTGTGATGTATTATTTTGGTCCATCTAATATGAGGGGAATTTCATAAATATGAGTGGAAGCATAGAATATTTTATAATCATCATATATATTACTAAAAGCAAAAAAAAAAAAAAAAAAAAAAAAAAA

>F-A1

AGGTACTTAGCGACTTCCTCACTCACTTTCCTTGAGGGCACCCTTGGGTGAAGAATAACCCCACATTACCAACAAGGAGAGTGATGAGATTCAGGGAAGGTCTCATTGCCAGTTTTCTCAGCATGAACCCTAATAGTTCTCTTCATCATAGTATTCTTCCCCATAAGAACAACAGAATCACCTCTCAAACCCTTTCTAATACTCTGTAGCTGATTAGATCCAACATTATCAGCAGCAGCCACAAGCACCTGAGTAAAATCATCCAGAAGTTGACACATTTTGGTGTCATAAGCAATCTTCTTCTCAGCCTTAGTTGCTTTAGGAGCCATTTGAACTGGTTTTTTCAGATGAAAACTAACCCTAAAAGTGAAAAGAAGAGATTTTTAGTACCTGC

>F-A11

GAGGTACTGCGTGATCCCTCGCTTTGGAGGCAACTTCCTCAAAATGAAAAGGACAAGTGAAGAAGGTAGAATCTCTACCAACAGATAGTATACAAAATTTAACATTGGATGATATAAAACATCAAGATCCGCAGCTTTATCAAATGCATTGAAACACATCATAATGCATCTAATCAGGAAGCATGAAAAACATATTGTTGTCACATAACCAACTTCCTGAAGTCTTCTTCTGTCTCCCCCTTGATTCTACACCTGCCC

>F-A4

CCCGGGCAGGTACCTTTATGTTCCACTACAGCAGCACGAAAATCTTCCACGATGGAGGCATCCTTGAACTTCAGAGCAATAGTAGAAAGTCCTTTTCCGTCAGCAGCACTATTCAAGCAAGCGAAAGTGACCCCTCTTTTGTCCATATTAGCAAGCTTCATTTCTGGAAAAAGGCTGGCATTCAAGATCAATCTGTAATTTCCTTTGGTCCTCATAACAAGTCTACCTTTTCCTTCCCCTGTTGTAGAAACATTGACCTTTAGTTCTCCCTTCCCCCGCTCTTTCCACCCTCCATTAAGATATTCAAACAGCACGGAATCAGCTGCGAATACGGGTTTTTCATTTTCCTCCCCTGTTTCAACAGTGACCTCTTCTTTGGAAGGAAATCCAGTACCTC

>F-B7

TGGCTTGCTGCCACGATCCACTGAGATTCCAGCCCTTTGTCCGCTTCCGATTCGTCTGCAAAGGATTCCTACCCGCCGCTCGATGGAAATTGTACCTCGGCCGCGACCACGC

>F-B8

GCCGAGGTACTTGCCATGACGAGGGTCACACTTAACCATCATAGAAGATGGCTCAAAGGCACTGTTAGTGATTTCTGCAACTGAAAGCTGCTCATGGTAGGCCTTCTCAGCAGAAATGACGGGGGCATATGAGGAAAGCATAAAATGGATCCTGGGATAGGGGACAAGATTGGTCTGGAATTCATTCACATCAACATTCAGAGCTCCATCAAACCTCAAAGAAGCTGTCAGAGAAGATATAACCTGTGAGATAAGGCGATTGAGATTGGTGTAGGTAGGGCGCTCAATGTCCAATGAGCGCCTGCAAATGTCATAGATGGCTTCATTGTCCAAAAGGATTGACACATCAGTGTGCTCAAGTAGAGAATGAGTTGACAGGACACTGTTGTAAGGCTCAACAACAGAAGTTGAGACCTGTGGTGAGGGATAAATTGTGAAACCAAGTTTGGATTTCTTTCCATAGTCAACTGAGAGACGCTCCAGCAGAAGTGACCCAAGACCTGAACCGGTACCTGCC

>F-C1

ATATCATCTTACATAGAACTAGTTTAGGATTCACAAACAACTGAGAAATATATAATGGGAAAGAACAGTAGAATATTTAAATTACTAAGCAGTAATAATTTGAAGAAAATGCCTCTGATTGAACATGACTGCTAATTAATTAATTAGCAGAAAAAAAGGAAATCAAGGCACTTATATATATAAAAAAAACAACAAATGATGACTTAATAAATATGAAACATAATTCTTCCAGAGCTATATATATAGCAAATCCCTTAAGTGTTCAGCAATAACCTTCTCATGAAGACACAATTCATCACTTTTCATTTTTTGGATCAAGAAAATGGGTGAGAAGAAGTGCAATCATCATCTCCACCTCGGC
